# Nickel-catalyzed migratory alkyl–alkyl cross-coupling reaction†

**DOI:** 10.1039/d0sc03217d

**Published:** 2020-09-09

**Authors:** Yangyang Li, Yuqiang Li, Long Peng, Dong Wu, Lei Zhu, Guoyin Yin

**Affiliations:** The Institute for Advanced Studies, Wuhan University Wuhan 430072 China yinguoyin@whu.edu.cn; Institute of Biomedical Materials Industry Technology, Hubei Engineering University Hubei 432000 China Lei.zhu@hbeu.edu.cn

## Abstract

The selective cross-coupling of activated electrophiles with unactivated ones has been regarded as a challenging task in cross-electrophile couplings. Herein we describe a migratory cross-coupling strategy, which can overcome this obstacle to access the desired cross-coupling products. Accordingly, a selective migratory cross-coupling of two alkyl electrophiles has been accomplished by nickel catalysis. Remarkably, this alkyl–alkyl cross-coupling reaction provides a platform to prepare 2°–2° carbon–carbon bonds from 1° and 2° carbon coupling partners. Preliminary mechanistic studies suggest that chain-walking occurs at both alkyl halides in this reaction, thus a catalytic cycle with the key step involving two alkylnickel(ii) species is proposed for this transformation.

## Abstract 2

The selective cross-coupling of activated electrophiles with unactivated ones has been regarded as a challenging task in cross-electrophile couplings.

## Graphical abstract

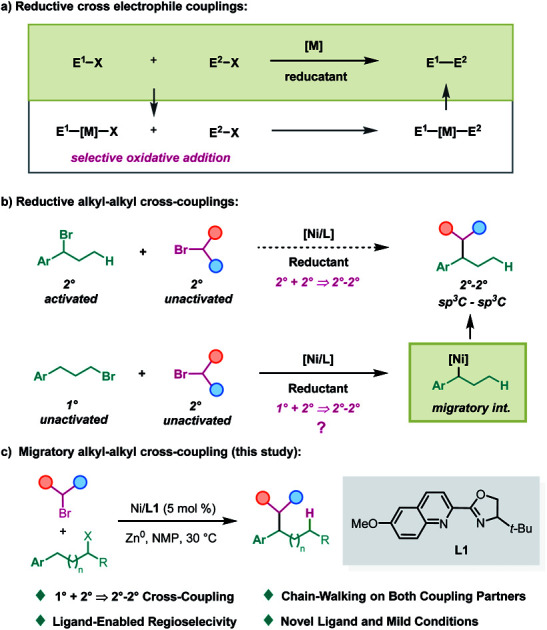

## Introduction

**Scheme 1:**
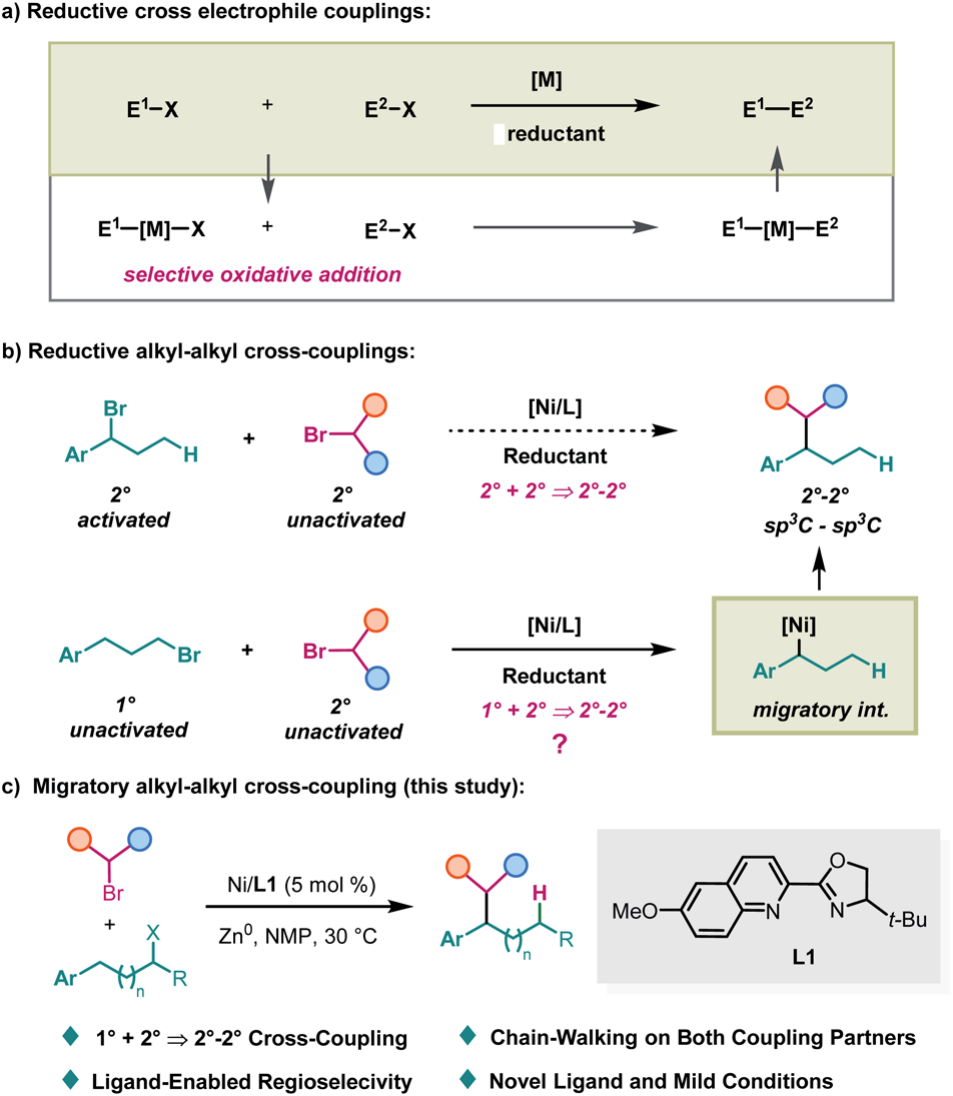
Nickel-catalyzed migratory cross-coupling of alkyl electrophiles.

Selective cross-coupling of two electrophiles has been developed into a general procedure to construct carbon–carbon bonds in recent years, owing to their advantage in avoiding handling of air and moisture-sensitive metal reagents.^1^ Particularly, great achievements have been accomplished in the cross-coupling of two carbon electrophiles with nickel catalysis in the presence of a cheap metal reductant.^2^ Because of the selective oxidative addition involved in these reactions, matching electrophiles are always required to achieve efficient chemoselectivity (Scheme 1a).^3^ To solve this problem, an elegant dual metal cooperative catalysis strategy was introduced by the Weix group,^4^ which particularly enables the cross-coupling of two sp^2^-carbon electrophiles or an sp^2^-carbon electrophile with an sp^3^-carbon electrophile. However, generally speaking, the selective cross-couplings of unactivated electrophiles with activated ones have still been regarded as an invincible task in this arena to date. On the other hand, in contrast, the reductive cross-coupling of two alkyl electrophiles^2*c*^ has been less developed.^5^ The efficient nickel-catalyzed cross-coupling of two alkyl halides was demonstrated by Gong^3*a*,6^ and MacMillan^7^ independently, wherein C(sp^3^)–C(sp^3^) bonds still cannot be constructed at benzylic positions. Herein, we report a migratory strategy to break the above obstacle to access the products of cross-coupling of unactivated electrophiles with activated ones (Scheme 1b), and achieve a benzylic selective alkyl–alkyl cross-coupling reaction under mild conditions (Scheme 1c). Moreover, this strategy also provides a platform to construct 2°–2° carbon–carbon bonds from 1° and 2° carbon partners.

As an extension of our interest in nickel chain-walking,^8,9^ we suspected whether a migratory alkyl–alkyl cross-coupling could be achieved. The challenge with regard to this idea is the chemoselectivity due to the difficulty in differentiating the two alkyl electrophiles in oxidative addition and both coupling partners may undergo β-hydride elimination.

## Results and discussion

To test the viability of a nickel-catalyzed migratory reductive alkyl–alkyl cross-coupling reaction, a primary alkyl bromide **1a** and a secondary one **2a** were chosen as model substrates. After extensive screening of every reaction parameter, we were delighted to find that when the reaction was conducted with 5 mol% NiI_2_ as the precatalyst, a novel PyrOx derivative **L1** as the ligand,^10^ the cheap zinc dust as the stoichiometric reductant, with the addition of LiBr, *N*-methyl pyrrolidone (NMP) as the solvent, at 30 °C for 24 hours, 70% of **3a** was isolated with excellent regioselectivity (27 : 1) (Table 1, entry 1). The employment of a PyrOx type ligand was crucial to the success of this reaction, which was demonstrated by the fact that no more than a trace amount of product was detected with other nitrogen-based ligands, such as 6,6′-dimethyl-2,2′-bipyridine (**L3**), bathocuproine (**L4**) and 1,2-diimine (**L5**) (Table 1, entries 1–5), and no migratory product formation was observed in the control reaction without the ligand (Table 1, entry 6). Notably, when 2,2′-bipyridine or 1,10-phenanthroline was used, the non-migratory 1°–2° cross-coupling product was formed as the major product (please see ESI Table S1 for details†). Different nickel(ii) precatalysts also showed big differences in reactivity, for example NiCl_2_ and NiBr_2_ only afforded very low yields of the desired product (Table 1, entries 7 and 8), the reason is still unclear. Replacing NMP with dimethylacetamide (DMA) resulted in a lower yield, but no product was formed with dimethylformide (DMF) (Table 1, entries 9 and 10). Replacing zinc dust with magnesium dust led to a lower yield and lower regioselectivity (Table 1, entry 11). The additive was also important for this transformation, which was highlighted by replacing LiBr with other salts; it resulted in dramatically decreasing yields and without LiBr there was no desired product formation (Table 1, entries 12–15). It probably accelerates the reduction of Ni(ii) to Ni(0) at the Zn surface.^11^ Finally, no appreciable enantioselectivity was afforded with a single enantiomer of PyrOx ligand.
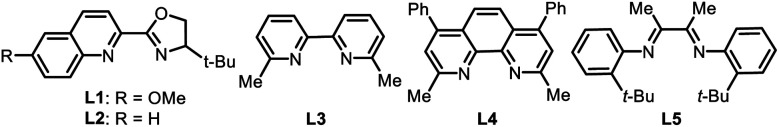


**Table 1:** Reaction development^a^

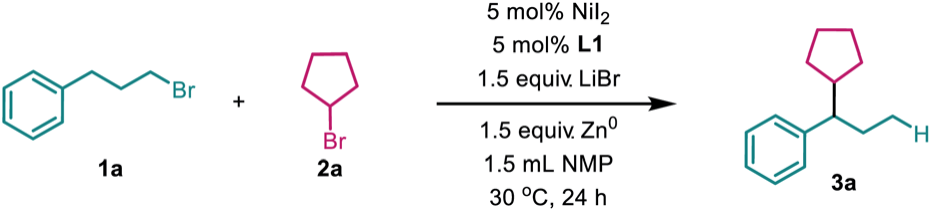			
Entry	Deviation from standard conditions	Yield [%]	*rr* b
1	No	74(70)^c^	27 : 1
2	**L2** instead of **L1**	52	10 : 1
3	**L3** instead of **L1**	4	—
4	**L4** instead of **L1**	Trace	—
5	**L5** instead of **L1**	Trace	—
6	No ligand	0	—
7	NiCl_2_ instead of NiI_2_	Trace	—
8	NiBr_2_ instead of NiI_2_	5	—
9	DMF instead of NMP	Trace	—
10	DMA instead of NMP	50	8 : 1
11	Mn instead of Zn	24	4 : 1
12	*n*-BuN_4_Br instead of LiBr	30	11 : 1
13	NaBr instead of LiBr	Trace	—
14	LiI instead of LiBr	Trace	—
15	No LiBr	Trace	—

a The reactions are conducted on a 0.5 mmol scale; GC yields against naphthalene.

b Regioisomeric ratio (*rr*) refers to the ratio of **3a** with other isomers, which is determined by GC-MS analysis of the reaction mixtures.

c Isolated yield of **3a**.

With the optimal conditions in hand, we next turned our attention towards investigating the generality of this migratory reaction. As shown in Table 2, a series of unactivated primary alkyl bromides with 2- to 7-carbon chains were tested, and the corresponding benzylic alkylation products could be generated in moderate to good yields with good to excellent regioselectivity. The electronic properties of the aryl group did not show an obvious effect on both the efficiency and the regioselectivity. Notably, unactivated alkyl chlorides could also furnish the desired alkylation products in moderate yield, with a good regioisomeric ratio (**3a** and **3f**). Remarkably, 2°–2° carbon–carbon bonds could also be constructed when secondary alkyl bromides were used in this system with moderate yield (**3v**, **3w** and **3x**). It is noteworthy that nickel chain-walking was able to cross the carbon chain with a branch barricade in this reaction (**3y**). A series of functional groups, such as ether, aryl chloride, ester, ketone, free phenol and indole were all quite compatible with this reaction. However, a few limitations were also identified. For example, substrates bearing aniline (**3z**), amide (**3aa**), and cyano (**3ab**) groups led to only trace products, probably due to their strong coordinating ability inhibiting β-H eliminations.

**Table 2:** Scope of primary alkyl halides^a^

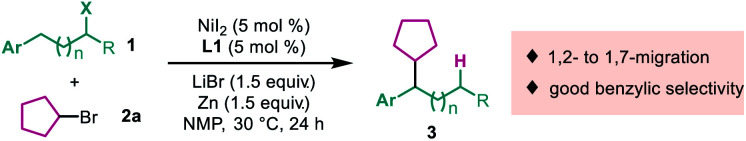
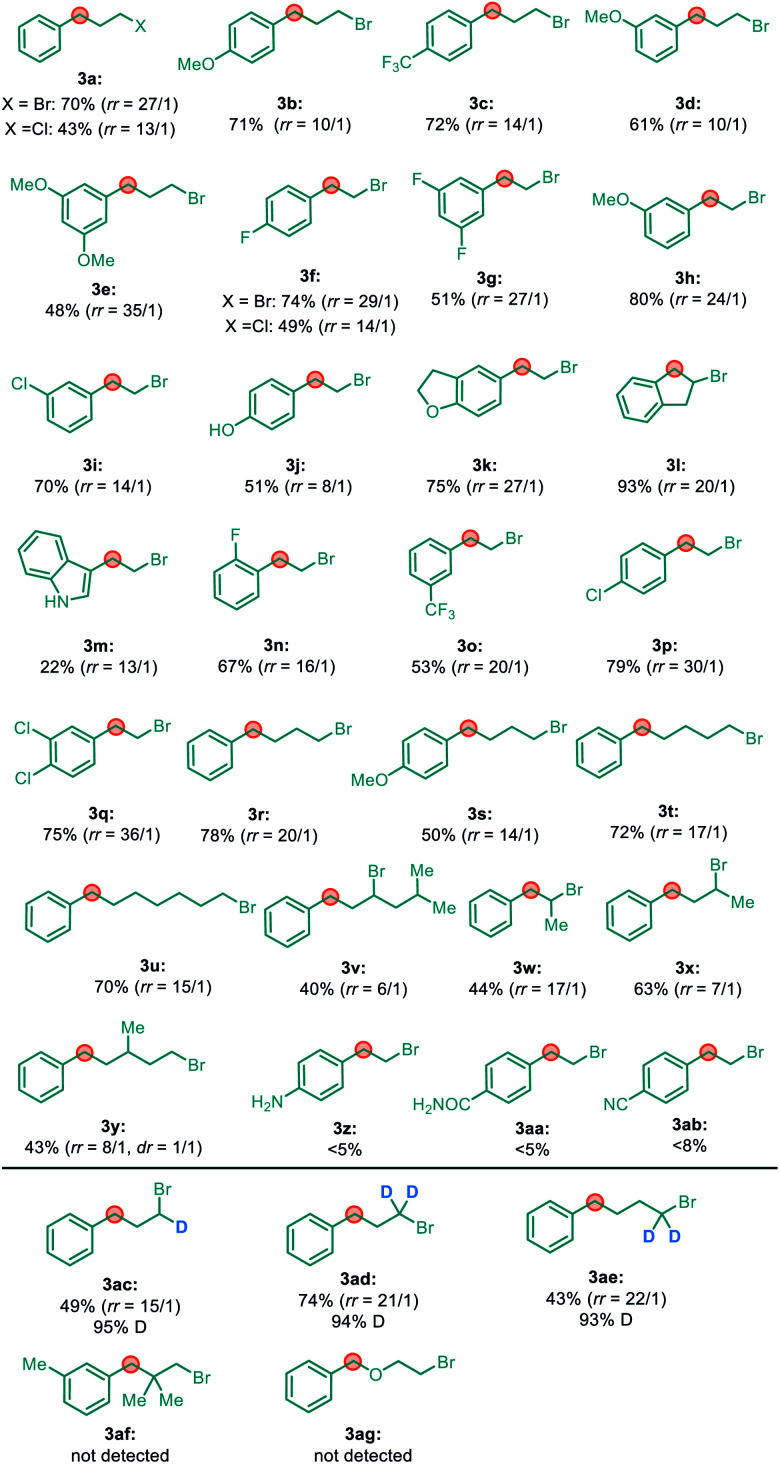

a Isolated yield of the major isomer at 0.5 mmol scale; regioisomeric ratio (*rr*) is determined by GC-MS analysis of the reaction mixture.

Remarkably, α-D substituted alkyl bromides gave rise to terminal, partially D-labeled products with very high deuterium retention (>98%) (**3ac**, **3ad** and **3ae**). In addition, no migratory cross-coupling product was detected in the reaction with alkyl bromide **3af** or **3ag**. These findings strongly suggest that the formation of migratory cross-coupling products does not involve the formation of an alkyl radical and then a 1,*n*-hydrogen atom transfer (HAT) occurred to form a benzylic radical, which were identified as the key steps.^12^

**Scheme 2:**
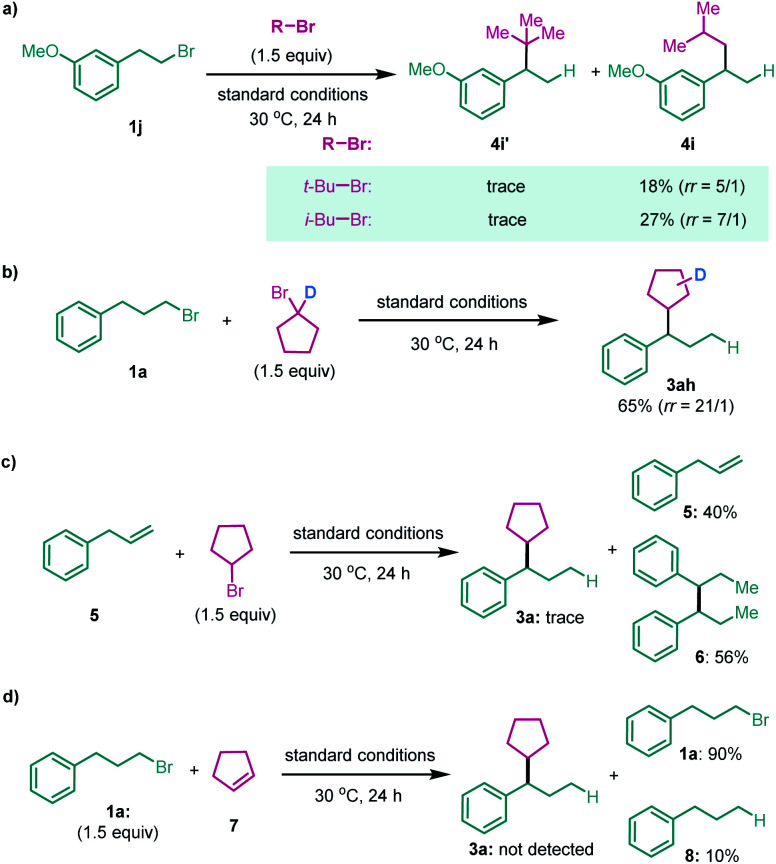
Mechanistic investigations.

The secondary alkyl halide part was examined subsequently (Table 3). Other cyclic alkyl bromides, with 6 to 7-member rings, were all able to furnish the migratory cross-coupling products in synthetically useful yields (**4a–4f**). Heterocyclic alkyl bromides were also tested in this reaction. Surprisingly, *N*-benzyl 4-bromopiperidine selectively yielded the product **4g** in 35% yield, with only the primary alkyl partner migration. A primary alkyl bromide such as *i*-BuBr was examined next, which afforded the cross-coupling product in a relatively low yield and low selectivity with the product formed by single alkyl partner migration being the major product (**4h**). However, we found that both *t*-BuBr and *i*-BuBr selectively gave rise to migration coupling products (Scheme 2a). These results indicate that both 2°–2° and 2°–1° carbon–carbon bonds can be constructed in this system, but the construction of sterically bulkier 2°–3° carbon–carbon bonds is still a challenging task. Finally, a deuterium-labeled cyclopentyl bromide was prepared and tested in the reaction; the cross-coupling product with deuterium migrating to the five-member ring was isolated in 65% yield (Scheme 2b).^13^ This finding suggests that nickel chain-walking occurs in both coupling partners.^14^

**Table 3:** Scope of secondary alkyl halides^a^

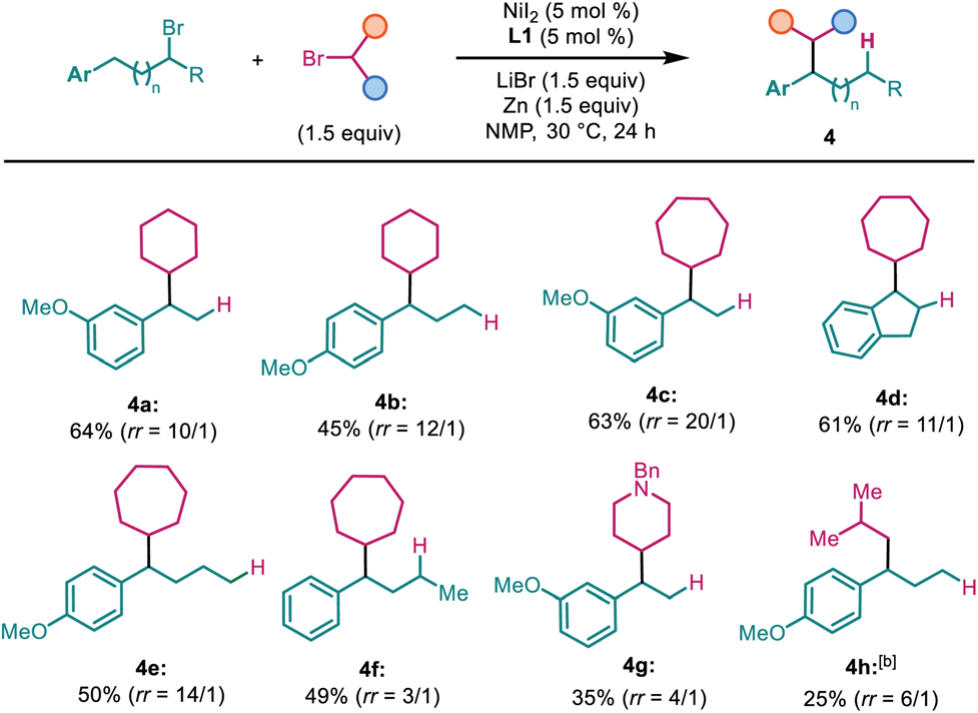

a Isolated yield of the major isomer at 0.5 mmol scale; regioisomeric ratio (*rr*) is determined by GC-MS analysis of the reaction mixture.

b From *i*-Bu-Br.

Recent advances in Ni-catalyzed migratory hydrofunctionalization of alkenes prompted several efforts to address the possibility of involving alkenes as intermediates.^13^ However, replacement of **1a** with allylbenzene **5** led to only trace **3a**, with the reductive homocoupling product **6** formed in 56% yield instead (Scheme 2c). In addition, no desired product but debromination of **1a** was observed when using cyclopentene **7** as the coupling partner (Scheme 2d). These results suggest that the related alkenes less likely serve as intermediates in this reaction. Although more studies still need to devote to drawing a detailed mechanistic profile, we believe that the reaction proceeds through nickel(ii) chain-walking on both coupling partners^15^ and cross-coupling of these two distinct alkyl-Ni(ii) species led to the final product.

## Conclusions

In summary, we have developed an alkyl–alkyl cross-coupling reaction, which can access the products of unactivated electrophiles with activated ones. This reaction constitutes the first example of metal migration occurring at 1° and 2° alkyl carbon coupling partners to construct 2°–2° carbon–carbon bonds. The success of this transformation is attributed to the application of a sterically hindered nitrogen-based ligand. Preliminary mechanistic investigations suggest that chain-walking happens at both coupling partners. Further mechanistic investigations are underway in our laboratory currently.

## Conflicts of interest

There are no conflicts to declare.

## Supplementary Material

SC-011-D0SC03217D-s001
