# Supplementary material for: Nickel-catalyzed migratory alkyl–alkyl cross-coupling reaction
Source: Chem Sci. 2020 Sep 9;11(38):10461–4. doi: 10.1039/d0sc03217d (PMC8162388; doi:10.1039/d0sc03217d)
Supplement: SC-011-D0SC03217D-s001 [file SC-011-D0SC03217D-s001.pdf]

Electronic Supplementary Material (ESI) for Chemical Science.
This journal is © The Royal Society of Chemistry 2020

Electronic Supplementary Information

Nickel-Catalyzed Reductive Migratory Alkyl-Alkyl Cross-Coupling Reaction

Yangyang Li,[a] Yuqiang Li,[a] Long Peng,[a] Dong Wu,[a] Lei Zhu,*[b] and Guoyin Yin*[a]

[a] Institute for Advanced Studies, Wuhan University, Wuhan 430072, China

[b] Institute of Biomedical Materials Industry Technology, Hubei Engineering University, Hubei 432000, China.

*corresponding author,

[b]E-mail: Lei.zhu@hbeu.edu.cn

[a]E-mail: yinguoyin@whu.edu.cn

Contents

1. General Information 3

2. Reaction Optimization 4

3. Synthesis of Substrates 7

   3.1 General Procedure (B) for Synthesis of Alkyl Bromines 7

   3.2 General Procedure (C) for Synthesis of Alkyl Bromines 7

   3.3 Synthesis of Dueterium-Labeled Alkyl Bromides 8

   3.4 Synthesis of (3-Bromopropyl-3-d)benzene 11

   3.5 Synthesis of 1-Bromocyclopentane-1-d 13

4. Synthesis of Ligands 14

5. Analytical Data of Compounds 16

6. References 26

7. NMR Spectra 27

1. General Information

General information: All reactions were run under a dry argon atmosphere fitted on 8 mL vials unless otherwise noted. Thin layer chromatography (TLC) employed glass 0.25 mm silica gel plates. Flash chromatography columns were packed with 200-300 mesh silica gel in petroleum (bp. 60-90 ℃). GC-MS spectra were recorded on a Varian GC-MS 3900-2100T. All new compounds were characterized by ^{1}H NMR, ^{13}C NMR, ^{19}F NMR and HRMS. The known compounds were characterized by ^{1}H NMR, ^{13}C NMR. ^{1}H ^{13}C and ^{19}F NMR data were recorded with Bruker 400 MHz with tetramethylsilane as an internal standard. Data for ^{1}H ^{13}C and ^{19}F NMR are reported as follows: chemical shift (δ ppm), multiplicity (s = singlet, d = doublet, t = triplet, q = quartet, qui = quintet, dd = doublet of doublet, dt = doublet of triplet, dq = doublet of quartet, m = multiplet), integration, and coupling constant (Hz). All chemical shifts (δ) were reported in ppm and coupling constants (*J*) in Hz. All chemical shifts were reported relative to tetramethylsilane (0 ppm for ^{1}H), Chloroform-d (77.16 ppm for ^{13}C), respectively. GC analyses were performed on an Agilent 7890B gas chromatograph with an FID detector using a *J* & W DB-1 column (10 m, 0.1 mm I.D.). High resolution mass spectra (HRMS) were measured with a Waters Micromass GCT instrument.

Materials: NiI_2 (CAS Nu: 13462-90-3) was purchased from sigma-aldrich. Bathocuproine (BC, CAS Nu: 4733-39-5) and anhydrous NMP and LiBr were purchased from Adamas-beta®. *n*-Bu_4NBr (TBAB, CAS Nu:1643-19-2) was purchased from Tokyo Chemical Industry. $LiAlD_4$ (95% D) was purchased from AMEKO. Unless otherwise noted, alkyl acids, and alkyl bromides were obtained from commercial suppliers (Energy Chemical, Adamas-beta®, *J*&K and so on) and used without further purification.

2. Reaction Optimization

Table S1. Preliminary Reaction Optimization [a]

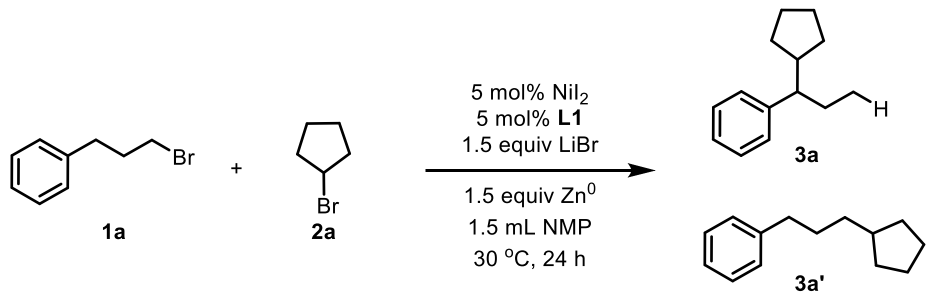

entry	deviation from standard conditions	**3a** Yield [%]	**3a’** Yield [%]	rr [**3a**/**3a’**]
1	no	74(70)[b]	3	27:1
2	L2 instead of L1	52	5	10:1
3	L3 instead of L1	4	Trace	-
4	L4 instead of L1	Trace	Trace	-
5	L5 instead of L1	Trace	Trace	-
6	L6 instead of L1	Trace	67	1:>20
7	L7 instead of L1	Trace	53	1:>20
8	no ligand	0	0	-
9	$NiCl_2$ instead of NiI_2	Trace	Trace	-
10	$NiBr_2$ instead of NiI_2	5	Trace	-
11	DMF instead of NMP	Trace	Trace	-
12	DMA instead of NMP	50	6	8:1
13	THF instead of NMP	Trace	Trace	-
14	MeCN instead of NMP	Trace	Trace	-
15	NMP (2.0 mL)	51	5	10:1
16	Mn instead of Zn	24	6	4:1

17	n-BuN_4Br instead of LiBr	30	3	11:1
18	NaBr instead of LiBr	Trace	Trace	-
19	LiI instead of LiBr	Trace	Trace	-
20	no LiBr	Trace	Trace	-
21	10 mol% (NiI_2/L1)	68	4	15:1
22	2 mol% (NiI_2/L1)	14	3	4:1

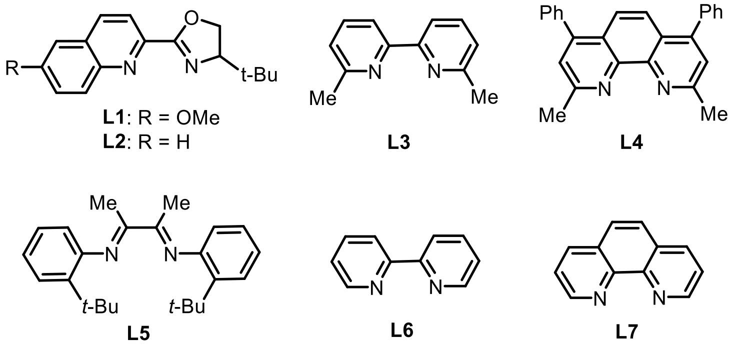

[a] **Standard conditions**: NiI_2 (7.8 mg, 0.05 mmol, 5 mol %), L1 (7.2 mg, 0.05 mmol, 5 mol %), 1a (75 ul, 0.5 mmol, 1.0 equiv), 2a (80 mg, 0.75 mmol, 1.5 equiv), LiBr (65 mg, 0.75 mmol, 1.5 equiv), Zn (49 mg, 0.75 mmol, 1.5 equiv), NMP (1.5 mL). Yields were determined by GC with naphthalene as the internal standard. [b] Isolated yield.

General procedure A : Under Nitrogen atmosphere, into an oven-dried 10 mL reaction tube equipped with a magnetic stir bar and sealed with a rubber stopper sequentially added NiI_2 (7.8 mg, 0.05 mmol, 5 mol %), **L1** (7.2 mg, 0.05 mmol, 5 mol %), **1a** (75 ul, 0.5 mmol, 1.0 equiv), **2a** (80 mg, 0.75 mmol, 1.5 equiv), LiBr (65 mg, 0.75 mmol, 1.5 equiv), Zn (49 mg, 0.75 mmol, 1.5 equiv), NMP (1.5 mL). The mixture was stirred at 30 ℃ for 24 h. The reaction was extracted with acetate (3 ×10 mL), and then the solvent was removed under reduced pressure, and the residue was purified by column chromatography on silica gel to afford the products.

Table S2. Ineffective Substrates

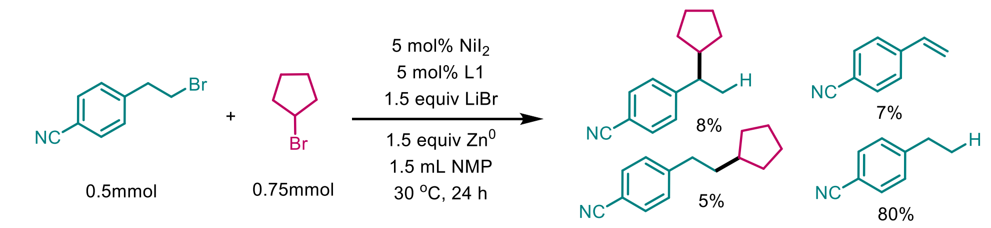

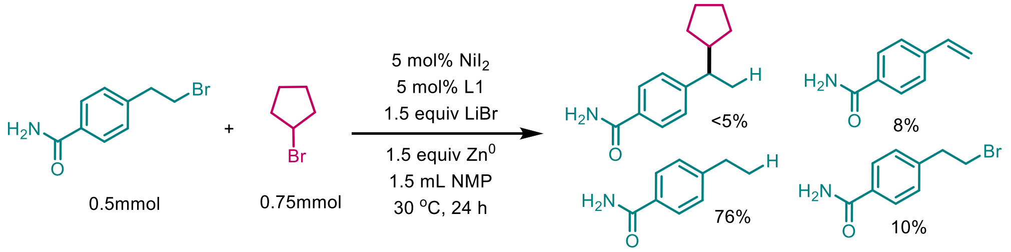

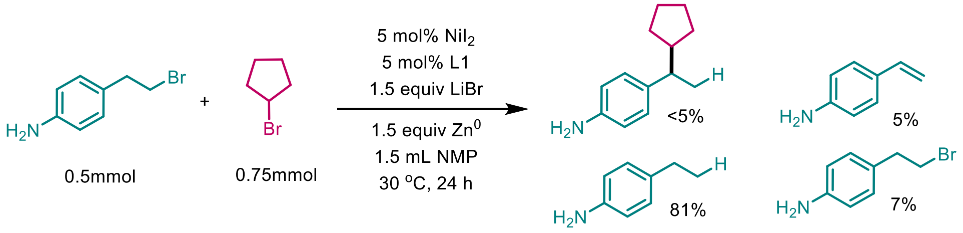

Functional group tolerance studies

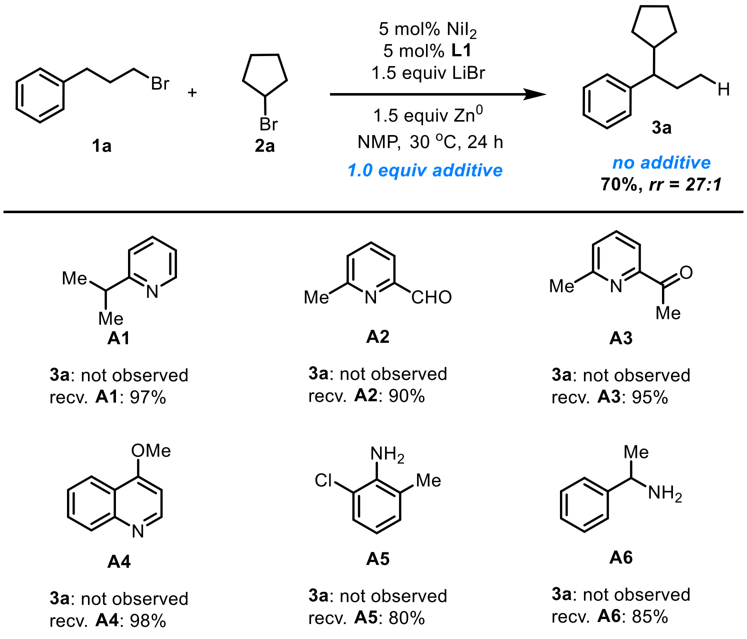

3. Synthesis of Substrates

3.1 General Procedure (B) for Synthesis of Alkyl Bromines

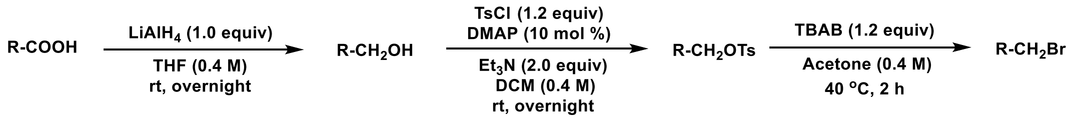

General procedure for the reduction of carboxylic acid[1]: To a stirred solution of $LiAlH_4$ (1.0 equiv) in THF (0.4 M) was added a solution of carboxylic acid (1.0 equiv) in THF dropwise at 0 °C. The mixture was stirred at 0 ℃ for another 1 h and then allowed to warm to room temperature for 12 h. The reaction progress was monitored by thin-layer chromatography (TLC). The reaction was quenched with 10% NaOH, then the mixture was extracted with EtOAc (3 × 40 mL), and the organic layers were combined and concentrated in vacuo to give a crude material of alcohol, which was used directly in the next step without further purification.

General procedure for the alcohol tosylation[2]: To a solution of corresponding starting alcohol (1.0 equiv) in DCM (0.4 M), TsCl (1.2 equiv), DMAP (10 mol %) and Et_3N (2 equiv) were added. The reaction mixture was stirred rapidly at room temperature for 12 h. The reaction progress was monitored by thin-layer chromatography (TLC). After completion of the reaction, the mixture was extracted with DCM (3 × 40 mL), and the organic layers were combined and concentrated in vacuo to give a crude material of alkyl tosylate. The crude product was purified via flash chromatography over silica gel.

General procedure for the alkyl bromines: To a solution of corresponding starting alcohol tosylate (1.0 equiv) in Acetone (0.4 M), TBAB (1.2 equiv) was added. The reaction mixture was stirred rapidly at 40 ℃ for 2 h. The reaction progress was monitored by thin-layer chromatography (TLC). After completion of the reaction, the mixture was concentrated in vacuo to give a crude material of alkyl tosylate. The crude product was then purified via flash chromatography over silica gel.

3.2 General Procedure (C) for Synthesis of Alkyl Bromines

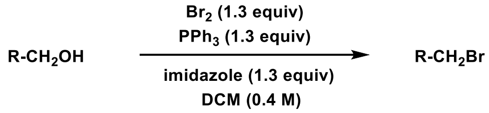

To a solution of PPh_3 (1.3 equiv) and imidazole (1.3 equiv) in anhydrous DCM (0.4 M), Br_2 was added slowly. The reaction mixture was stirred rapidly at 0 °C . Then alcohol was added dropwise. The mixture was stirred at 0 ℃ for another 1 h and then allowed to warm to room temperature for 12 h. The reaction progress was monitored by thin-layer chromatography (TLC). After completion of the reaction, the

mixture was quenched with saturated aqueous $NaHCO_3$ and extracted with DCM (3 × 40 mL), and the organic layers were combined and concentrated in vacuo to give a crude material of alkyl bromine. The crude product was purified via flash chromatography over silica gel.

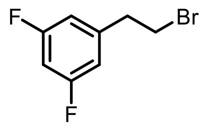

1-(2-bromoethyl)-3,5-difluorobenzene (2.2 g, 51 %, a colorless liquid.**):** prepared according to the **general procedure B.** ^{1}H NMR (400 MHz, Chloroform-*d*) δ 6.80 - 6.66 (m, 3 H), 3.55 (t, *J* = 7.3 Hz, 2 H), 3.15 (t, *J* = 7.3 Hz, 2 H).

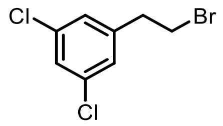

1-(2-bromoethyl)-3,5-dichlorobenzene (2.8 g, 59%, a colorless liquid.**):** prepared according to the **general procedure B.** ^{1}H NMR (400 MHz, Chloroform-*d*) δ 7.39 (d, *J* = 8.21 Hz, 1 H), 7.31 (d, *J* = 2.08 Hz, 1 H), 7.06 (dd, *J* = 8.19, 2.10 Hz, 1 H), 3.54 (t, *J* = 7.25 Hz, 2 H), 3.12 (t, *J* = 7.25 Hz, 2 H).

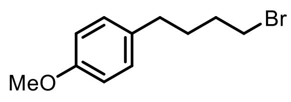

1-(4-bromobutyl)-4-methoxybenzene (2.5 g, 54%, a colorless liquid.**):** prepared according to the **general procedure B**. ^{1}H NMR (400 MHz, Chloroform-*d*) δ 7.11 (d, *J* = 8.28 Hz, 2 H), 6.85 (d, *J* = 8.26 Hz, 2 H), 3.80 (s, 3 H), 3.43 (t, *J* = 6.82 Hz, 2 H), 2.61 (t, *J* = 7.58 Hz, 2 H), 1.90 (p, *J* = 7.06 Hz, 2 H), 1.76 (p, *J* = 7.60 Hz, 2 H).

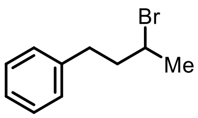

(3-bromobutyl)benzene (2.0 g, 96%, a colorless liquid.**):** prepared according to the **general procedure C**. ^{1}H NMR (400 MHz, Chloroform-*d*) δ 7.21 (m, 2 H), 7.12 (m, 3 H), 4.01 (m, 1 H), 2.80 (m, 1 H), 2.67 (m, 1 H), 2.11 - 2.02 (m, 1 H), 2.01 - 1.92 (m, 1 H), 1.65 (d, *J* = 6.7 Hz, 3 H).

3.3 Synthesis of Dueterium-Labeled Alkyl Bromides

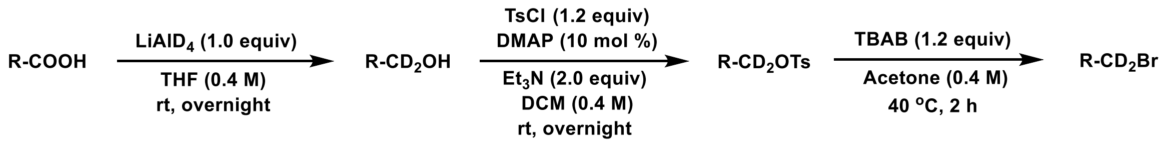

General procedure for the dueterium-labeled alkyl bromides: According to the **general procedure B.**

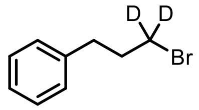

(3-bromopropyl-3,3-d2)benzene (0.2 g, 36%, 94% D**):** a colorless liquid. ^{1}H NMR (400 MHz, Chloroform-*d*) δ 7.32 (td, *J* = 6.99, 1.54 Hz, 2 H), 7.27 - 7.14 (m, 3 H), 2.80 (t, *J* = 7.42 Hz, 2 H), 2.18 (t, *J* = 7.38 Hz, 2 H). ^{13}C NMR (101 MHz, Chloroform-*d*) δ 140.7, 128.7, 128.6, 126.3, 34.0, 34.0, 32.80 (dt, *J* = 46.46, 23.30 Hz).

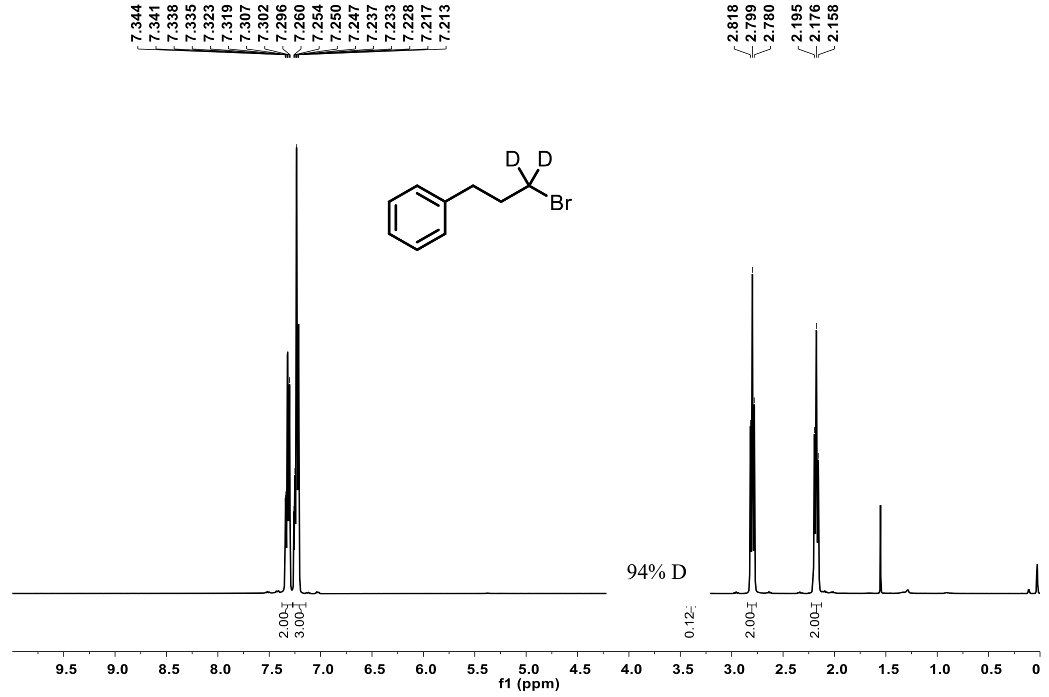

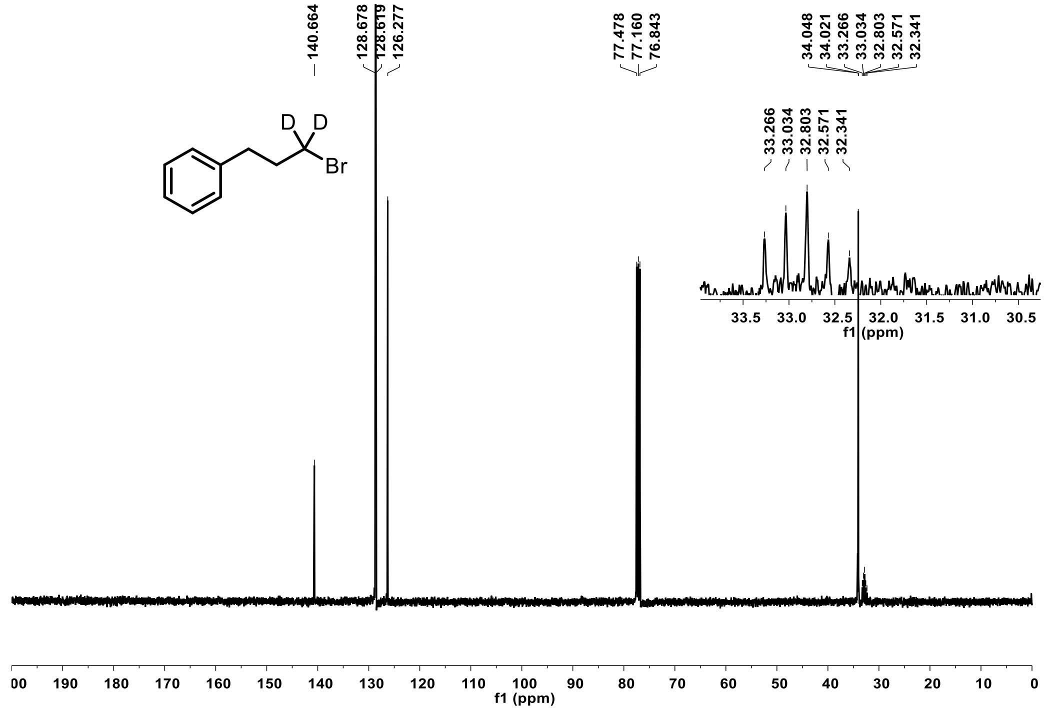

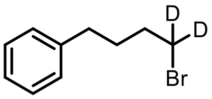

(4-bromobutyl-4,4-d2)benzene (0.2 g, 39%, 94% D**):** a colorless liquid. ^{1}H NMR (600 MHz, Chloroform-d) δ 7.30 (dd, J = 8.44, 6.36 Hz, 2 H), 7.25 - 7.14 (m, 3 H), 2.65 (t, J = 7.50 Hz, 2 H), 1.89 (dd, J = 8.90, 5.83 Hz, 2 H), 1.83 - 1.73 (m, 2 H). ^{13}C NMR (151 MHz, Chloroform-d) δ 141.9, 128.5, 126.0, 35.1, 33.7 (tt, *J* = 13.41 Hz), 32.1, 29.9. **Note**: one aryl carbon signal is missing due to overlapping, which is consistent with a precedent report (Org. Lett. 2012, 14, 4842–4845. See: SI page 2)

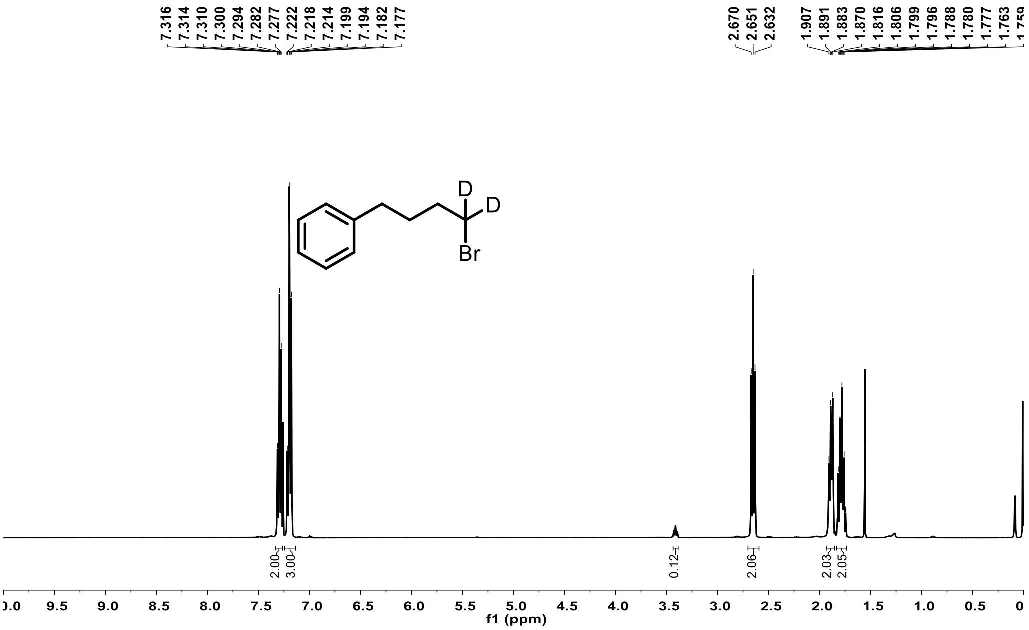

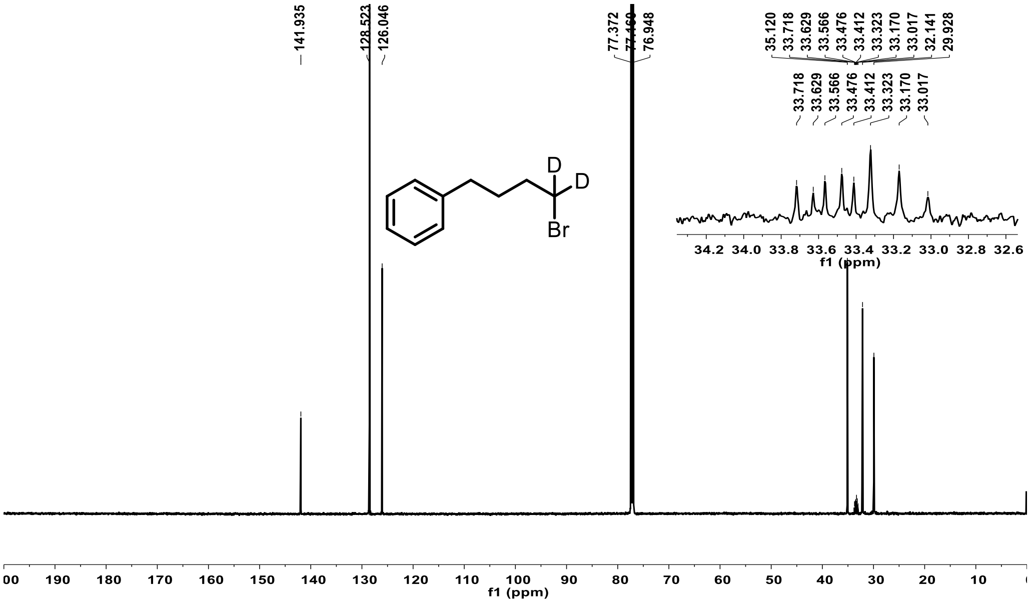

3.4 Synthesis of (3-Bromopropyl-3-d)benzene

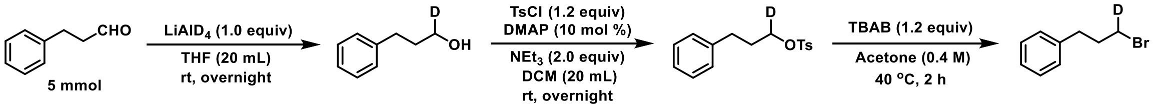

Procedure for the reduction of 3-phenylpropanal[1]: To a stirred solution of $LiAlD_4$ (210 mg, 5 mmol, 1.0 equiv) in THF (20 mL) was added a solution of 3-phenylpropanal (0.7 mL, 5 mmol, 1.0 equiv) in THF dropwise at 0 ℃. The mixture was stirred at 0 °C for another 1 h and then allowed to warm to room temperature for 12 h. The reaction progress was monitored by thin-layer chromatography (TLC).The reaction was quenched with 10% NaOH, then the mixture was extracted with EtOAc (3 × 40 mL), and the organic layers were combined and concentrated in vacuo to give a crude material of 3-phenylpropan-1-*d*-1-ol, which was used without further purification.

Procedure for 3-phenylpropan-1-*d*-1-ol tosylation[2] **:** To a solution of 3-phenylpropan-1-d-1-ol (0.7 mL, 5 mmol, 1.0 equiv) in DCM (20 mL), TsCl (1.2 g, 6 mmol, 1.2 equiv), DMAP (61 mg, 0.5 mmol, 10 mol %) and Et_3N (1.4 mL, 10 mmol, 2 equiv) were added. The reaction mixture was stirred rapidly at room temperature for 12 h. The reaction progress was monitored by thin-layer chromatography (TLC). After completion of the reaction, the mixture was extracted with DCM (3 × 40 mL), and the organic layers were combined and concentrated in vacuo to give a crude material. The crude product was purified via flash chromatography over silica gel.

Procedure for (3-bromopropyl-3-d)benzene: To a solution of corresponding 3-phenylpropan-1-d-1-ol tosylation (1.0 equiv) in Acetone (0.4 M), TBAB (1.2 equiv) was added. The reaction mixture was stirred rapidly at 40 ℃ for 2 h. The reaction progress was monitored by thin-layer chromatography (TLC). After completion of the reaction, the mixture was concentrated in vacuo to give a crude material of alkyl tosylate. The crude product was then purified via flash chromatography over silica gel.

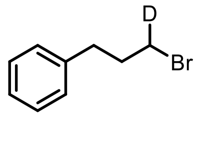

(3-bromopropyl-3-d)benzene (0.5 g, 48%, 96% D)**:** a colorless liquid. ^{1}H NMR (400 MHz, Chloroform-d) δ 7.36 - 7.26 (m, 2 H), 7.22 (td, J = 6.43, 1.64 Hz, 3 H), 3.39 (tt, J = 6.53, 1.65 Hz, 1 H), 2.79 (t, J = 7.36 Hz, 2 H), 2.17 (q, J = 7.18 Hz, 2 H).^{13}C NMR (101 MHz, Chloroform-d) δ 140.7, 128.7, 128.6, 126.3, 34.2, 34.1, 33.1 (t, J = 23.35 Hz).

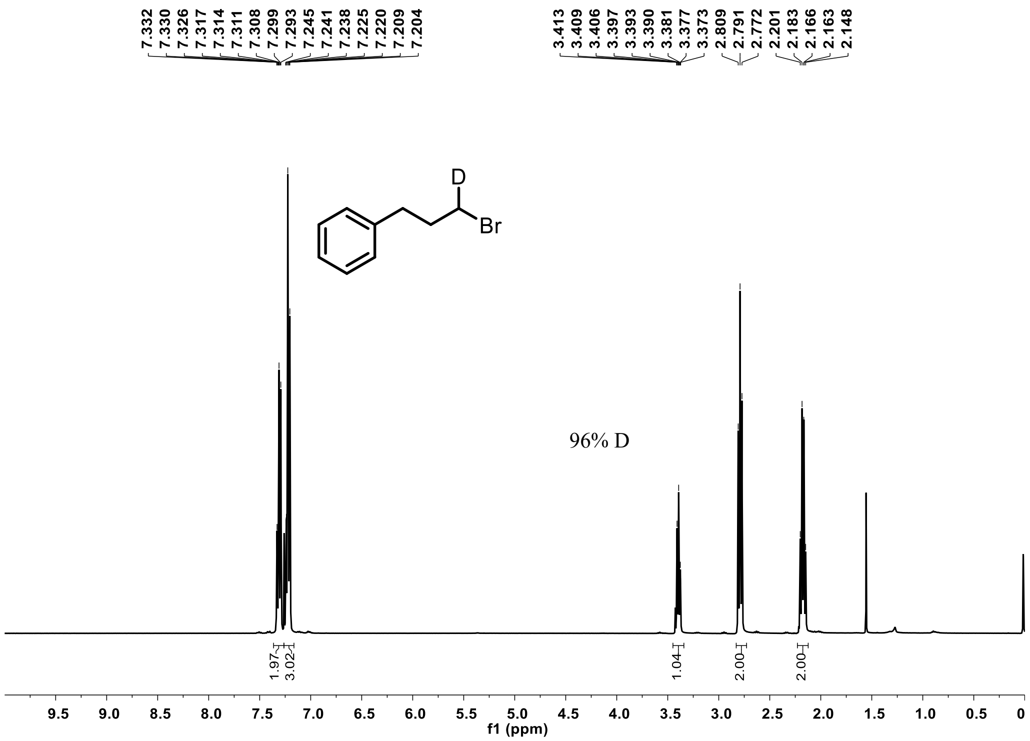
7.332
7.330
7.326
7.317
7.314
7.311
7.308
7.299
7.293
7.245
7.241
7.238
7.225
7.220
7.209
7.204
3.413
3.409
3.406
3.397
3.393
3.390
3.381
3.377
3.373
2.809
2.791
2.772
2.201
2.183
2.166
2.163
2.148
D
Br
96% D
1.97
3.02
1.04
2.00
2.00
f1 (ppm)

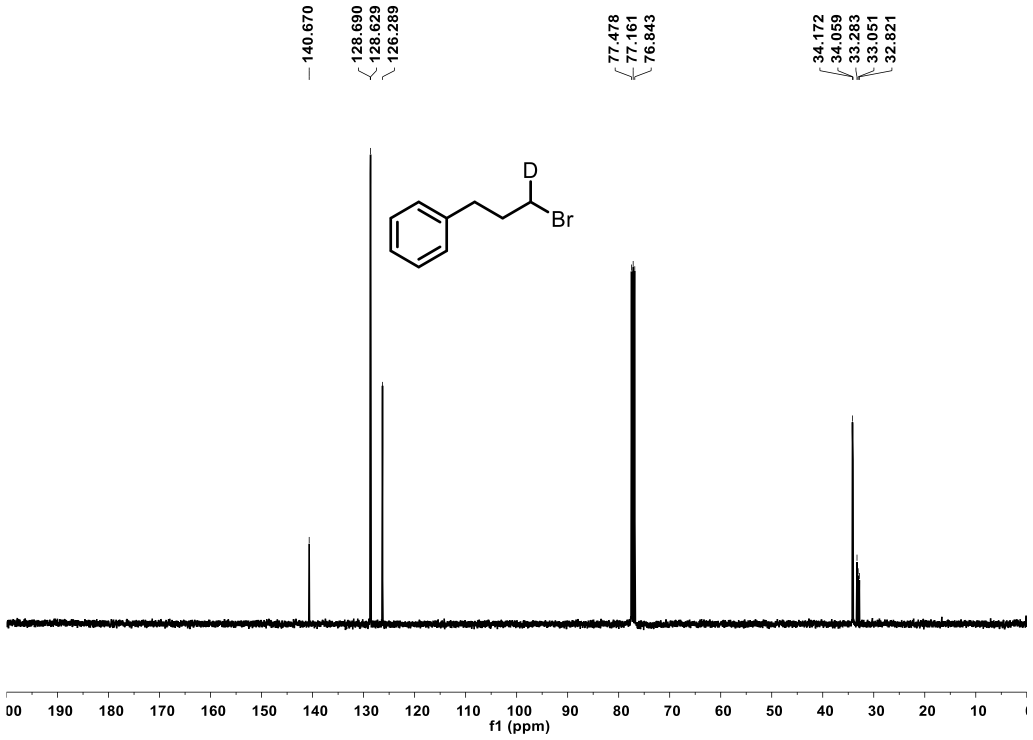
140.670
128.690
128.629
126.289
77.478
77.161
76.843
34.172
34.059
33.283
33.051
32.821
D
Br
f1 (ppm)

3.5 Synthesis of 1-Bromocyclopentane-1-d

Procedure method according to 3.4

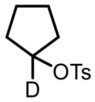

cyclopentyl-1-d 4-methylbenzenesulfonate (0.8 g, 65%, 98% D) 1H NMR (400 MHz, Chloroform-*d*) δ 7.85 (d, J = 7.75 Hz, 2 H), 7.40 (d, J = 7.82 Hz, 2 H), 2.52 (s, 3 H), 1.91 - 1.73 (m, 6 H), 1.67 - 1.56 (m, 2 H).

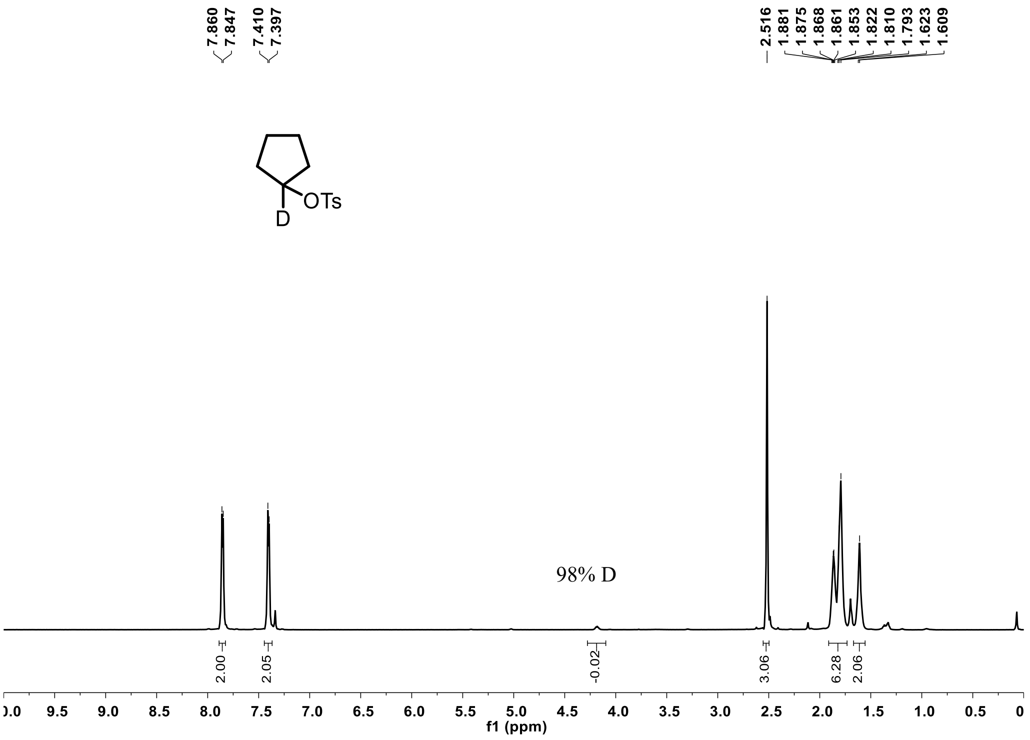

4. Synthesis of Ligands

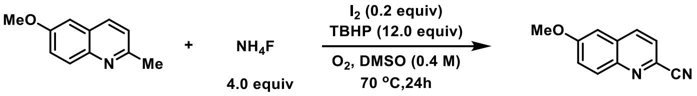

Synthesis of 6-methoxyquinoline-2-carbonitrile : Under O2, a 200 mL of Schlenk flask equipped with a stir bar was charged with 6-methoxy-2-methylquinoline (5.2 g ,30 mmol), I2 (10.2 mg, 0.04 mmol), NH4F (4.5 g, 120 mmol), TBHP (70% in water, 48.6 mL, 260 mmol), DMSO (50 mL). The reaction mixture was stirred at 70 ℃ for 48 h in oil bath. After the completion of the reaction (monitored by TLC), the solvent was extracted by EtOAc (3 × 40 mL) and the organic layers were combined and concentrated in vacuo and the residue was purified by flash column chromatography on silica gel with petroleum ether-EtOAc as the eluent to give the desired product(3.0 g, 54%, a white solid).

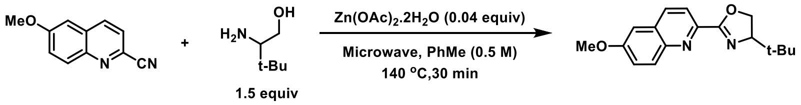

Synthesis of 6-methoxyquinoline-2-carbonitrile : a 10 mL of microwave tube equipped with a stir bar was charged with 6-methoxyquinoline-2-carbonitrile (921 mg, 5 mmol), 2-amino-3,3-dimethylbutan-1-ol (879 mg, 7.5 mmol), Zn(OAc)2.2H2O (43.9 mg, 0.2 mmol), PhMe (5 mL). The reaction mixture was stirred at 140 ℃ for 30 min. After the completion of the reaction (monitored by TLC), the solvent was extracted by EtOAc (3 × 40 mL) and the organic layers were combined and concentrated in vacuo and the residue was purified by flash column chromatography on silica gel with petroleum ether-EtOAc as the eluent to give the desired product(924 mg, 65%, a white solid). 1H NMR (400 MHz, Chloroform-d) δ 8.24 - 8.05 (m, 3H), 7.39 (dd, J = 9.27, 2.78 Hz, 1H), 7.09 (d, J = 2.77 Hz, 1H), 4.52 (dd, J = 10.25, 8.74 Hz, 1H), 4.38 (t, J = 8.46 Hz, 1H), 4.16 (dd, J = 10.26, 8.18 Hz, 1H), 3.94 (s, 3H), 1.00 (s, 9H). ^{13}C NMR (101 MHz, Chloroform-d) δ 162.9, 158.9, 144.7, 143.7, 135.3, 131.9, 130.2, 123.0, 121.5, 105.0, 76.6, 69.6, 55.7, 34.2, 26.1. HRMS (ESI) Calculated for $C_{17}H_{20}N_2O_2$ ($[M+H]^+$): 285.1598, measured: 285.1599.

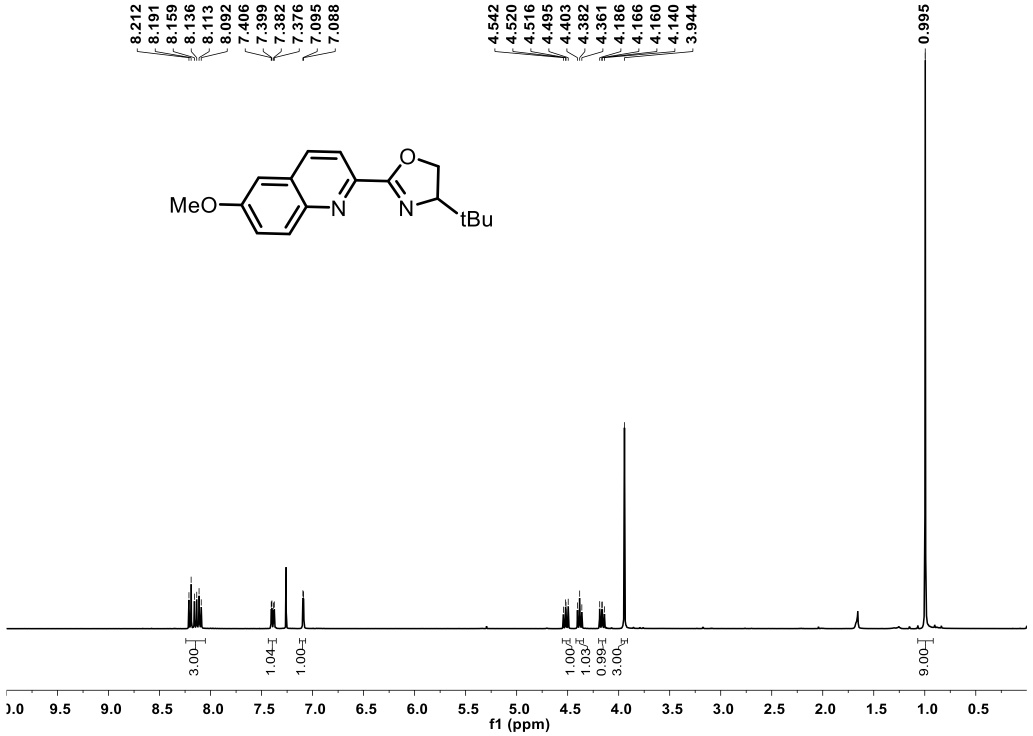
8.212
8.191
8.159
8.136
8.113
8.092
7.406
7.399
7.382
7.376
7.095
7.088
4.542
4.520
4.516
4.495
4.403
4.382
4.361
4.186
4.166
4.160
4.140
3.944
0.995
MeO
N
O
N
tBu
3.00
1.04
1.00
1.00
1.03
0.99
3.00
9.00
f1 (ppm)

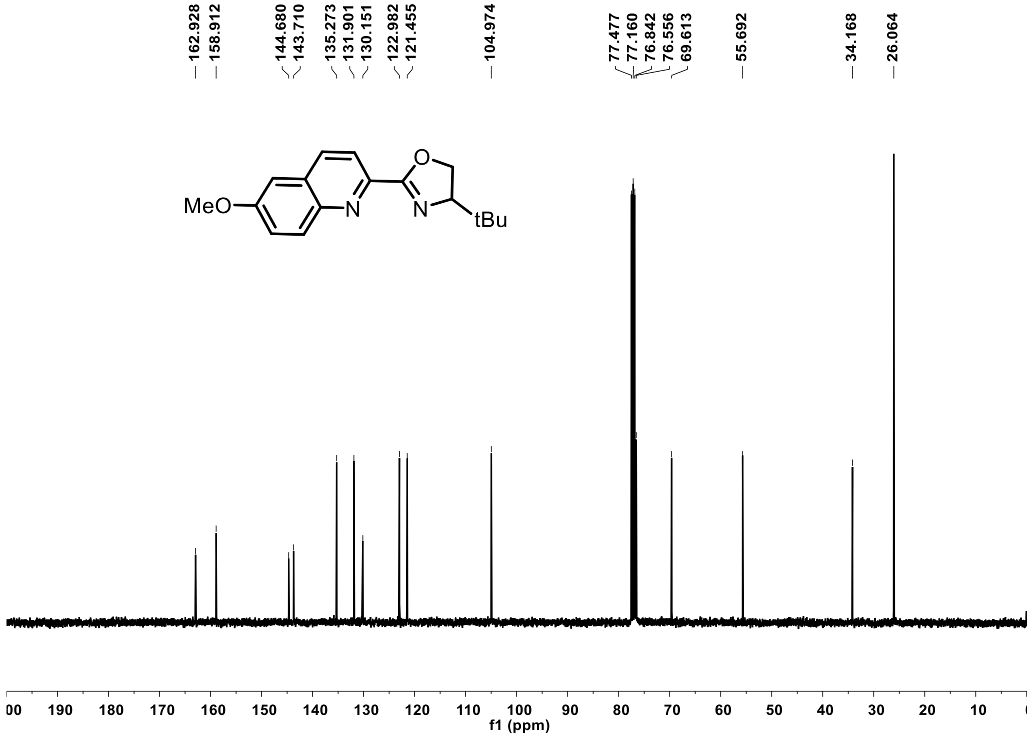
162.928
158.912
144.680
143.710
135.273
131.901
130.151
122.982
121.455
104.974
77.477
77.160
76.842
76.556
69.613
55.692
34.168
26.064
MeO
N
O
N
tBu
f1 (ppm)

5. Analytical Data of Compounds

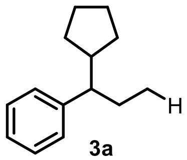

3a

(1-cyclopentylpropyl)benzene (3a) [3]**:** The reaction was conducted following the general procedure **A** in a 0.5 mmol scale. The residue was purified by column chromatography on silica gel to afford the product **3a** (X = Br : 41.4 mg, 70% yield, *rr* = 27/1; X= Cl : 25.4 mg, 43% yield, *rr* = 13/1) as a colorless oil. ^{1}H NMR (400 MHz, Chloroform-*d*) δ 7.28 - 7.25 (m, 2 H), 7.18 - 7.16 (m, 1 H), 7.15 - 7.11 (m, 2 H), 2.15 (td, *J* = 10.3, 3.6 Hz, 1 H), 2.05 - 1.78 (m, 3 H), 1.66 - 1.55 (m, 2 H), 1.54 - 1.28 (m, 4 H), 1.26 - 1.15 (m, 1 H), 1.00 - 0.90 (m, 1 H), 0.68 (t, *J* = 7.4 Hz, 3 H) ppm. ^{13}C NMR (101 MHz, Chloroform-*d*) δ 145.7, 128.2, 128.0, 125.6, 54.2, 46.5, 31.9, 31.6, 28.1, 25.3, 24.9, 12.2 ppm.

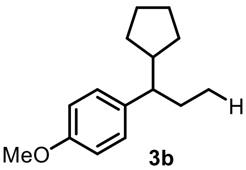

3b

1-(1-cyclopentylpropyl)-4-methoxybenzene (3b): The reaction was conducted following the general procedure **A** in a 0.5 mmol scale. The residue was purified by column chromatography on silica gel to afford the product **3b** (77.5 mg, 71% yield, *rr* = 10/1) as a colorless oil. ^{1}H NMR (400 MHz, Chloroform-*d*) δ 7.06 - 7.02 (m, 2 H), 6.84 - 6.80 (m, 2 H), 3.79 (s, 3 H), 2.11 (td, *J* = 10.18, 3.61 Hz, 1 H), 2.01 - 1.86 (m, 2 H), 1.83 - 1.75 (m, 1 H), 1.68 - 1.58 (m, 1 H), 1.55 - 1.43 (m, 3 H), 1.42 - 1.29 (m, 2 H), 1.23 - 1.13 (m, 1 H), 1.01 - 0.88 (m, 1 H), 0.68 (t, *J* = 7.37 Hz, 3 H) ppm. ^{13}C NMR (101 MHz, Chloroform-*d*) δ 157.6, 137.9, 129.0, 113.4, 55.3, 53.4, 46.7, 31.9, 31.7, 28.2, 25.5, 25.0, 12.3 ppm. HRMS (ESI) Calculated for $C_{15}H_{24}O$ ([M+H]$^+$): 218.1743, measured: 218.1723.

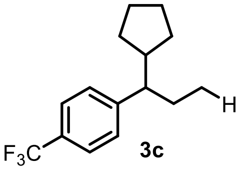

3c

1-(1-cyclopentylpropyl)-4-(trifluoromethyl)benzene (3c) [3]**:** The reaction was conducted following the general procedure **A** in a 0.5 mmol scale. The residue was purified by column chromatography on silica gel to afford the product **3c** (92.3 mg, 72% yield, *rr* = 14/1) as a colorless oil. ^{1}H NMR (400 MHz, Chloroform-*d*) δ 7.52 (d, *J* = 7.98 Hz, 2 H), 7.24 (d, *J* = 8.0 Hz, 2 H), 2.24 (td, *J* = 10.3, 3.6 Hz, 1 H), 2.05 - 1.82 (m, 3 H), 1.68 - 1.58 (m, 1 H), 1.55 - 1.50 (m, 2 H), 1.49 - 1.16 (m, 4 H), 0.97 - 0.87 (m, 1 H), 0.67 (t, *J* = 7.4 Hz, 3 H) ppm. ^{13}C NMR (101 MHz, Chloroform-*d*) δ 150.1 (q, *J* = 1.08 Hz), 128.5, 128.1 (q, *J* = 32.8 Hz), 125.1 (q, *J* = 3.79 Hz), 124.6 (q, *J* = 271.7 Hz), 54.3, 46.4, 31.9, 31.7, 28.1, 25.3, 25.0, 12.2 ppm. ^{19}F NMR (377 MHz, Chloroform-*d*) δ -62.15 ppm.

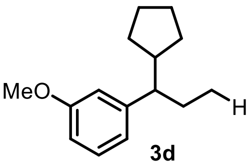

3d

1-(1-cyclopentylpropyl)-3-methoxybenzene (3d): The reaction was conducted following the general procedure A in a 0.5 mmol scale. The residue was purified by column chromatography on silica gel with petroleum ether to afford the product **3d** (66.6 mg, 61% yield, *rr* = 10:1) as a colorless oil. ^{1}H NMR (400 MHz, Chloroform-*d*) δ 7.19 (t, *J* = 7.8 Hz, 1 H), 6.73 (t, *J* = 9.1 Hz,

2 H), 6.69 (s, 1 H), 3.80 (s, 3 H), 2.13 (td, J = 10.4, 3.2 Hz, 1 H), 2.00 - 1.89 (m, 2 H), 1.84 - 1.79 (m, 1 H), 1.65 - 1.60 (m, 1 H), 1.56 - 1.47 (m, 3 H), 1.45 - 1.34 (m, 2 H), 1.23 - 1.16 (m, 1 H), 1.00 - 0.94 (m, 1 H), 0.70 (t, J = 7.3 Hz, 3 H) ppm; ^{13}C NMR (101 MHz, Chloroform-*d*) δ 159.4, 147.6, 128.9, 120.9, 114.3, 110.5, 55.2, 54.4, 46.6, 32.0, 31.7, 28.1, 25.4, 25.0, 12.3 ppm; HRMS (ESI) Calculated for $C_{15}H_{23}O$ ([M+H]$^+$): 218.1743, measured: 218.1723.

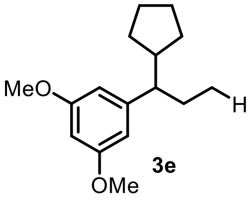

3e

1-(1-cyclopentylpropyl)-3,5-dimethoxybenzene (3e): The reaction was conducted following the general procedure **A** in a 0.5 mmol scale. The residue was purified by column chromatography on silica gel to afford the product **3e** (59.6 mg, 48% yield, *rr* = 35/1) as a colorless oil. ^{1}H NMR (400 MHz, Chloroform-*d*) δ 6.31 - 6.29 (m, 3 H), 3.78 (s, 6 H), 2.08 (td, J = 10.2, 3.6 Hz, 1 H), 2.00 - 1.87 (m, 2 H), 1.84 - 1.74 (m, 1 H), 1.57 - 1.35 (m, 6 H), 1.22 - 1.12 (m, 1 H), 1.04 - 0.93 (m, 1 H), 0.70 (t, J = 7.4 Hz, 3 H) ppm; ^{13}C NMR (101 MHz, Chloroform-*d*) δ 160.5, 148.6, 106.5, 97.2, 55.3, 54.7, 46.5, 31.9, 31.7, 28.1, 25.4, 25.0, 12.4 ppm; HRMS (ESI) Calculated for $C_{16}H_{25}O_2$ ([M+H]$^+$): 248.1838, measured: 248.1849.

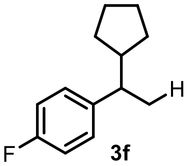

3f

1-(1-cyclopentylethyl)-4-fluorobenzene (3f) [3]**:** The reaction was conducted following the general procedure **A** in a 0.5 mmol scale. The residue was purified by column chromatography on silica gel to afford the product **3f** (X = Br : 77.1 mg,74 % yield, *rr* = 29/1; X = Cl : 47.1 mg, 49 % yield, rr = 14/1) as a colorless oil. ^{1}H NMR (400 MHz, Chloroform-*d*) δ 7.14 - 7.11 (m, 2 H), 6.98 - 6.94 (m, 2 H), 2.42 (dq, J = 14.0, 6.9 Hz, 1 H), 1.94 - 1.86 (m, 2 H), 1.70 - 1.52 (m, 3 H), 1.49 - 1.35 (m, 2 H), 1.28 - 1.17 (m, 4 H), 1.04 - 0.94 (m, 1 H) ppm; ^{13}C NMR (101 MHz, Chloroform-*d*) δ 161.2 (d, J = 242.70 Hz), 143.7 (d, J = 3.17 Hz), 128.6 (d, J = 7.57 Hz), 114.9 (d, J = 20.87 Hz), 47.9, 45.6, 31.9, 31.5, 25.5, 25.2, 21.8 ppm; ^{19}F NMR (377 MHz, Chloroform-*d*) δ -118.08.

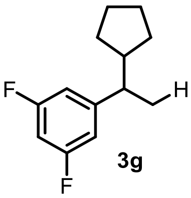

3g

1-(1-cyclopentylethyl)-3,5-difluorobenzene (3g): The reaction was conducted following the general procedure **A** in a 0.5 mmol scale. The residue was purified by column chromatography on silica gel to afford the product **3g** (53.6 mg, 51% yield, *rr* = 27/1) as a colorless oil. ^{1}H NMR (400 MHz, Chloroform-*d*) δ 6.72 - 6.58 (m, 3 H), 2.41 (dq, J = 13.5, 6.7 Hz, 1 H), 1.94 - 1.83 (m, 2 H), 1.69 - 1.38 (m, 5 H), 1.27 - 1.14 (m, 4 H), 1.05 - 0.95 (m, 1 H) ppm; ^{13}C NMR (101 MHz, Chloroform-*d*) δ 163.0 (dd, J = 247.2, 12.9 Hz), 152.3 (t, J = 8.2 Hz), 110.1 (dd, J = 18.0, 5.8 Hz), 101.2 (t, J = 25.4 Hz), 47.8, 46.4 (t, J = 1.8 Hz), 31.8, 31.4, 25.4, 25.2, 21.3 ppm; ^{19}F NMR (377 MHz, Chloroform-*d*) δ -110.92 ppm; HRMS (ESI) Calculated for $C_{13}H_{16}F_2Na$ ([M+Na]$^+$): 233.1096, measured: 233.1112.

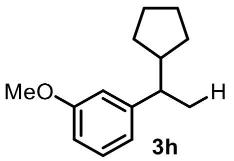

3h

1-(1-cyclopentylethyl)-3-methoxybenzene (3h) [3]: The reaction was conducted following the general procedure **A** in a 0.5 mmol scale. The residue was purified by column chromatography on silica gel to afford the product **3h** (81.7 mg, 80% yield, *rr* = 24/1) as a colorless oil. ^{1}H NMR (600 MHz, Chloroform-*d*) δ 7.22 (t, *J* = 7.7 Hz, 1 H), 6.80 (d, *J* = 7.3 Hz, 1 H), 6.76 - 6.74 (m, 2 H), 3.82 (s, 3 H), 2.42 (dq, *J* = 13.3, 7.3 Hz, 1 H), 1.98 - 1.91 (m, 2 H), 1.70 - 1.65 (m, 1 H), 1.61 - 1.54 (m, 2 H), 1.50 - 1.41 (m, 2 H), 1.30 - 1.20 (m, 4 H), 1.08 - 1.01 (m, 1 H) ppm; ^{13}C NMR (101 MHz, Chloroform-*d*) δ 159.5, 149.9, 129.1, 119.9, 113.4, 110.6, 55.2, 47.6, 46.4, 31.9, 31.6, 25.5, 25.2, 21.6.

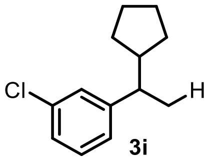

3i

1-chloro-3-(1-cyclopentylethyl)benzene (3i) [3]: The reaction was conducted following the general procedure **A** in a 0.5 mmol scale. The residue was purified by column chromatography on silica gel to afford the product **3i** (73.1 mg, 70% yield, *rr* = 14/1) as a colorless oil. ^{1}H NMR (400 MHz, Chloroform-*d*) δ 7.22 - 7.14 (m, 3 H), 7.06 (dt, *J* = 7.4, 1.4 Hz, 1 H), 2.41 (dq, *J* = 9.3, 6.9 Hz, 1 H), 1.98 - 1.87 (m, 2 H), 1.72 - 1.35 (m, 5 H), 1.25 (d, *J* = 6.9 Hz, 3 H), 1.22 - 1.17 (m, 1 H), 1.04 - 0.95 (m, 1 H) ppm; ^{13}C NMR (101 MHz, Chloroform-*d*) δ 150.4, 134.1, 129.6, 127.6, 126.1, 125.8, 47.7, 46.3, 32.0, 31.6, 25.4, 25.2, 21.5 ppm.

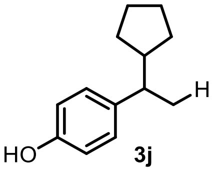

3j

4-(1-cyclopentylethyl)phenol (3j): The reaction was conducted following the general procedure **A** in a 0.5 mmol scale. The residue was purified by column chromatography on silica gel to afford the product **3j** (48.5 mg, 51% yield, *rr* = 8/1) as a white solid. ^{1}H NMR (400 MHz, Chloroform-*d*) δ 7.07 - 7.03 (m, 2 H), 6.77 - 6.74 (m, 2 H), 4.94 (s, 1 H), 2.37 (dq, *J* = 9.3, 6.9 Hz, 1 H), 1.93 - 1.84 (m, 2 H), 1.70 - 1.59 (m, 1 H), 1.59 - 1.49 (m, 2 H), 1.49 - 1.34 (m, 2 H), 1.22 (d, *J* = 6.9 Hz, 3 H), 1.21 - 1.14 (m, 1 H), 1.06 - 0.95 (m, 1 H) ppm; ^{13}C NMR (101 MHz, Chloroform-*d*) δ 153.4, 140.5, 128.4, 115.0, 47.9, 45.4, 31.9, 31.5, 25.5, 25.2, 21.8 ppm; HRMS (ESI) Calculated for $C_{13}H_{17}O$ ($[M-H]^-$): 189.1286, measured: 189.1285.

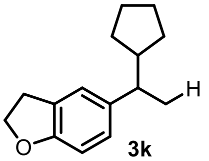

3k

5-(1-cyclopentylethyl)-2,3-dihydrobenzofuran (3k): The reaction was conducted following the general procedure **A** in a 0.5 mmol scale. The residue was purified by column chromatography on silica gel to afford the product **3k** (81.1 mg, 75% yield, *rr* = 27/1) as a colorless oil. ^{1}H NMR (400 MHz, Chloroform-*d*) δ 7.01 (s, 1 H), 6.92 - 6.89 (m, 1 H), 6.70 (d, *J* = 8.1 Hz, 1 H), 4.55 (t, *J* = 8.7 Hz, 2 H), 3.19 (t, *J* = 8.7 Hz, 2 H), 2.36 (dq, *J* = 9.0, 6.9 Hz, 1 H), 1.93 - 1.84 (m, 2 H), 1.71 – 1.62 (m,1 H), 1.60 - 1.49 (m, 2 H), 1.49 - 1.36 (m, 2 H), 1.22 (d, *J* = 6.9 Hz, 3 H), 1.21 - 1.14 (m, 1 H), 1.09 - 0.92 (m, 1 H) ppm; ^{13}C NMR (101 MHz, Chloroform-*d*) δ 158.1, 140.4, 126.8, 126.7, 123.7, 108.8, 71.2, 48.0, 45.7, 32.0, 31.6, 30.0, 25.5, 25.2, 22.0 ppm; HRMS (ESI) Calculated for $C_{15}H_{20}ONa$ ($[M+Na]^+$): 239.1414, measured: 239.1406.

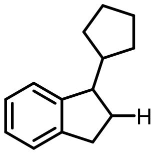
3l

1-cyclopentyl-2,3-dihydro-1H-indene (3l) [3]**:** The reaction was conducted following the general procedure **A** in a 0.5 mmol scale. The residue was purified by column chromatography on silica gel to afford the product **3l** (86.6 mg, 93% yield, *rr* = 20/1) as a colorless oil. ^{1}H NMR (400 MHz, Chloroform-*d*) δ 7.29 - 7.23 (m, 1 H), 7.22 - 7.17 (m, 1 H), 7.15 - 7.08 (m, 2 H), 3.06 (td, *J* = 7.9, 5.9 Hz, 1 H), 2.96 - 2.88 (m, 1 H), 2.83 - 2.75 (m, 1 H), 2.23 - 2.12 (m, 1 H), 2.10 - 1.98 (m, 1 H), 1.92 - 1.78 (m, 2 H), 1.78 - 1.69 (m, 1 H), 1.68 - 1.58 (m, 2 H), 1.58 - 1.48 (m, 2 H), 1.41 - 1.32 (m, 1 H), 1.27 - 1.18 (m, 1 H) ppm; ^{13}C NMR (101 MHz, Chloroform-*d*) δ 147.5, 144.5, 126.2, 125.9, 124.6, 124.5, 50.0, 44.5, 31.52, 31.50, 30.6, 30.4, 25.7, 25.3 ppm.

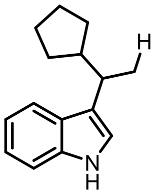
3m

3-(1-cyclopentylethyl)-1H-indole (3m): The reaction was conducted following the general procedure **A** in a 0.5 mmol scale. The residue was purified by column chromatography on silica gel to afford the product **3m** (23.5 mg, 22% yield, *rr* = 13/1) as a colorless oil. ^{1}H NMR (400 MHz, Chloroform-*d*) δ 7.88 (s, 1 H), 7.66 (d, *J* = 7.9 Hz, 1 H), 7.34 (d, *J* = 8.1 Hz, 1 H), 7.19 - 7.15 (m, 1 H), 7.11 - 7.07 (m, 1 H), 6.95 (d, *J* = 2.3 Hz, 1 H), 2.89 - 2.76 (m, 1 H), 2.21 - 2.14 (m, 1 H), 1.92 - 1.83 (m, 1 H), 1.66 - 1.58 (m, 1 H), 1.55 - 1.48 (m, 2 H), 1.34 (d, *J* = 7.0 Hz, 3 H), 1.32 - 1.24 (m, 2 H), 1.21 - 1.11 (m, 2 H) ppm; ^{13}C NMR (101 MHz, Chloroform-*d*) δ 136.4, 127.2, 122.8, 121.8, 120.4, 119.7, 119.0, 111.2, 47.1, 36.6, 31.8, 31.5, 25.7, 25.4, 20.9 ppm; HRMS (ESI) Calculated for $C_{15}H_{20}N$ ($[M+H]^+$): 214.1590, measured: 214.1594.

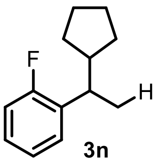
3n

1-(1-cyclopentylethyl)-2-fluorobenzene (3n) [3]**:** The reaction was conducted following the general procedure **A** in a 0.5 mmol scale. The residue was purified by column chromatography on silica gel to afford the product **3n** (64.4 mg, 67% yield, *rr* = 16/1) as a colorless oil. ^{1}H NMR (400 MHz, Chloroform-*d*) δ 7.27 (td, *J* = 7.4, 1.9 Hz, 1 H), 7.15 - 7.09 (m, 1 H), 7.07 - 7.03 (m, 1 H), 7.01 - 6.95 (m, 1 H), 2.89 (dq, *J* = 9.9, 6.9 Hz, 1 H), 2.06 - 1.98 (m, 1 H), 1.94 - 1.87 (m, 1 H), 1.76 - 1.66 (m, 1 H), 1.64 - 1.55 (m, 2 H), 1.55 - 1.43 (m, 2 H), 1.26 - 1.18 (m, 4 H), 1.08 - 0.98 (m, 1 H) ppm; ^{13}C NMR (101 MHz, Chloroform-*d*) δ 160.6 (d, *J* = 243.8 Hz), 134.4 (d, *J* = 14.8 Hz), 128.5 (d, *J* = 5.5 Hz), 126.9 (d, *J* = 8.4 Hz), 123.9 (d, *J* = 3.5 Hz), 115.2 (d, *J* = 23.4 Hz), 46.6 (d, *J* = 1.2 Hz), 38.5 (d, *J* = 1.4 Hz), 31.7, 31.6, 25.5, 25.2, 20.3 (d, *J* = 1.2 Hz) ppm; ^{19}F NMR (377 MHz, Chloroform-*d*) δ -118.71 ppm.

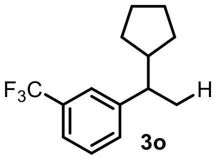
3o

1-(1-cyclopentylethyl)-3-(trifluoromethyl)benzene) (3o) [3]**:** The reaction was conducted following the general procedure **A** in a 0.5 mmol scale. The residue was purified by column chromatography on silica gel to afford the product **3o** (64.2 mg, 53% yield, *rr* = 20/1) as a colorless oil. ^{1}H NMR (400 MHz, Chloroform-*d*) δ 7.47 - 7.43 (m, 2 H), 7.41 - 7.35 (m, 2 H), 2.51 (dq, *J* = 9.4, 6.9 Hz, 1 H), 2.02 - 1.85

(m, 2 H), 1.74 - 1.62 (m, 1 H), 1.60 - 1.51 (m, 2 H), 1.51 - 1.42 (m, 1 H), 1.42 - 1.32 (m, 1 H), 1.28 (d, *J* = 6.9 Hz, 3 H), 1.26 - 1.18 (m, 1 H), 1.05 - 0.93 (m, 1 H) ppm; ^{13}C NMR (101 MHz, Chloroform-*d*) δ 148.9, 130.7 (d, *J* = 1.5 Hz), 130.42 (q, *J* = 31.7 Hz), 128.6, 124.4 (q, *J* = 272.2 Hz) 124.0 (q, *J* = 3.7 Hz), 122.6 (q, *J* = 3.9 Hz), 47.5, 46.2, 31.9, 31.5, 25.4, 25.2, 21.4 ppm; ^{19}F NMR (377 MHz, Chloroform-*d*) δ -62.42 ppm.

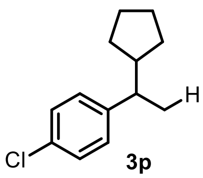

1-chloro-4-(1-cyclopentylethyl)benzene) (3p) [3]**:** The reaction was conducted following the general procedure **A** in a 0.5 mmol scale. The residue was purified by column chromatography on silica gel to afford the product **3p** (88.5 mg, 79% yield, *rr* = 30/1) as a colorless oil. ^{1}H NMR (400 MHz, Chloroform-*d*) δ 7.24 - 7.21 (m, 2 H), 7.11 - 7.07 (m, 2 H), 2.39 (dq, *J* = 9.0, 6.9 Hz, 1 H), 1.93 - 1.84 (m, 2 H), 1.67 - 1.32 (m, 5 H), 1.26 - 1.14 (m, 4 H), 1.01 - 0.91 (m, 1 H) ppm; ^{13}C NMR (101 MHz, Chloroform-*d*) δ 146.6, 131.3, 128.7, 128.4, 47.7, 45.8, 31.9, 31.5, 25.5, 25.2, 21.6 ppm.

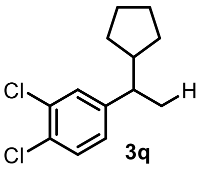

1,2-dichloro-4-(1-cyclopentylethyl)benzene (3q): The reaction was conducted following the general procedure **A** in a 0.5 mmol scale. The residue was purified by column chromatography on silica gel to afford the product **3q** (91.2 mg, 75% yield, *rr* = 36/1) as a colorless oil. ^{1}H NMR (400 MHz, Chloroform-*d*) δ 7.34 (d, *J* = 8.2 Hz, 1 H), 7.27 - 7.25 (m, 1 H), 7.03 - 7.00 (m, 1 H), 2.43 - 2.36 (m, 1 H), 1.93 - 1.84 (m, 2 H), 1.70 - 1.62 (m, 1 H), 1.59 - 1.35 (m, 4 H), 1.23 (d, *J* = 6.9 Hz, 3 H), 1.21 - 1.14 (m, 1 H), 1.03 - 0.93 (m, 1 H) ppm; ^{13}C NMR (101 MHz, Chloroform-*d*) δ 148.5, 132.1, 130.2, 129.5, 129.3, 126.9, 47.5, 45.7, 31.8, 31.5, 25.4, 25.2, 21.5 ppm; HRMS (EI) Calculated for $[C_{13}H_{16}Cl_2]^+$: 242.0629, $[M+2]^+$: 244.0594, $[M+4]^+$: 246.0565, measured: $[M]^+$: 242.0648, $[M+2]^+$: 244.0608, $[M+4]^+$: 246.0570.

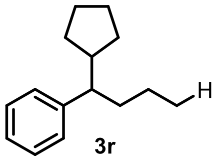

(1-cyclopentylbutyl)benzene (3r): The reaction was conducted following the general procedure **A** in a 0.5 mmol scale. The residue was purified by column chromatography on silica gel to afford the product **3r** (78.9 mg, 78% yield, *rr* = 20/1) as a colorless oil. ^{1}H NMR (400 MHz, Chloroform-*d*) δ 7.27 - 7.22 (m, 2 H), 7.18 - 7.11 (m, 3 H), 2.25 (td, *J* = 10.7, 3.6 Hz, 1 H), 2.03 - 1.83 (m, 2 H), 1.77 - 1.60 (m, 2 H), 1.60 - 1.46 (m, 3 H), 1.43 - 1.26 (m, 2 H), 1.24 - 1.13 (m, 1 H), 1.13 - 0.99 (m, 2 H), 0.99 - 0.87 (m, 1 H), 0.81 (t, *J* = 7.3 Hz, 3 H) ppm; ^{13}C NMR (101 MHz, Chloroform-*d*) δ 146.1, 128.2, 128.1, 125.7, 52.3, 46.9, 37.7, 32.0, 31.8, 25.4, 25.0, 20.8, 14.3 ppm; HRMS (ESI) Calculated for $C_{15}H_{22}Na$ ($[M+Na]^+$): 225.1613, measured: 225.1593.

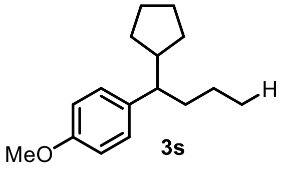

3s

1-(1-cyclopentylbutyl)-4-methoxybenzene (3s): The reaction was conducted following the general procedure **A** in a 0.5 mmol scale. The residue was purified by column chromatography on silica gel to afford the product **3s** (128.0 mg, 81% yield, *rr* = 16/1) as a colorless oil. ^{1}H NMR (400 MHz, Chloroform-*d*) δ 7.06 - 7.03 (m, 2 H), 6.84 - 6.81 (m, 2 H), 3.79 (s, 3 H), 2.21 (td, *J* = 10.3, 3.6 Hz, 1 H), 1.95 - 1.90 (m, 2 H), 1.72 - 1.62 (m, 2 H), 1.56 - 1.46 (m, 3 H), 1.45 - 1.37 (m, 1 H), 1.36 - 1.26 (m, 1 H), 1.23 - 1.13 (m, 1 H), 1.12 - 1.00 (m, 2 H), 0.99 - 0.89 (m, 1 H), 0.81 (t, *J* = 7.4 Hz, 3 H) ppm; ^{13}C NMR (101 MHz, Chloroform-*d*) δ 157.6, 138.2, 128.9, 113.4, 55.3, 51.3, 47.0, 37.8, 31.9, 31.7, 25.4, 25.0, 20.8, 14.3 ppm; HRMS (ESI) Calculated for $C_{16}H_{24}ONa$ ([M+Na]$^+$): 255.1719, measured: 218.1723.

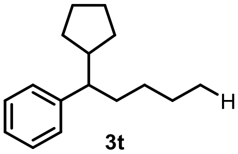

3t

(1-cyclopentylpentyl)benzene (3t): The reaction was conducted following the general procedure **A** in a 0.5 mmol scale. The residue was purified by column chromatography on silica gel to afford the product **3t** (77.9 mg, 72% yield, *rr* = 17/1) as a colorless oil. ^{1}H NMR (400 MHz, Chloroform-*d*) δ 7.29 - 7.24 (m, 2 H), 7.19 - 7.15 (m, 1 H), 7.14 - 7.11 (m, 2 H), 2.26 - 2.20 (m, 1 H), 2.04 - 1.88 (m, 2 H), 1.80 - 1.70 (m, 1 H), 1.65 - 1.52 (m, 3 H), 1.51 - 1.36 (m, 2 H), 1.34 - 1.25 (m, 2 H), 1.24 - 1.13 (m, 2 H), 1.06 - 0.92 (m, 3 H), 0.79 (t, *J* = 7.3 Hz, 3 H) ppm; ^{13}C NMR (101 MHz, Chloroform-*d*) δ 146.1, 128.1, 128.0, 125.6, 52.4, 46.8, 35.0, 31.8, 31.7, 29.8, 25.3, 24.9, 22.9, 14.1 ppm; HRMS (ESI) Calculated for $C_{16}H_{25}$ ([M+H]$^+$): 217.1960, measured: 217.1951.

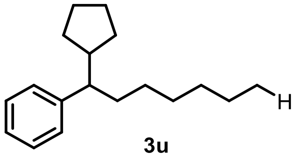

3u

(1-cyclopentylheptyl)benzene (3u): The reaction was conducted following the general procedure **A** in a 0.5 mmol scale. The residue was purified by column chromatography on silica gel to afford the product **3u** (85.6 mg, 70% yield, *rr* = 15/1) as a colorless oil. ^{1}H NMR (400 MHz, Chloroform-*d*) δ 7.20 - 7.17 (m, 2 H), 7.12 - 7.04 (m, 3 H), 2.16 (td, *J* = 10.2, 3.7 Hz, 1 H), 1.95 - 1.81 (m, 2 H), 1.72 - 1.61 (m, 1 H), 1.58 - 1.50 (m, 1 H), 1.47 - 1.38 (m, 3 H), 1.25 - 1.19 (m, 2 H), 1.16 - 1.04 (m, 7 H), 0.99 - 0.88 (m, 3 H), 0.76 (t, *J* = 6.9 Hz, 3 H) ppm; ^{13}C NMR (101 MHz, Chloroform-*d*) δ 146.2, 128.2, 128.1, 125.7, 52.5, 46.9, 35.4, 31.94, 31.92, 31.7, 29.6, 27.7, 25.4, 25.0, 22.8, 14.2 ppm; HRMS (ESI) Calculated for $C_{18}H_{29}$ ([M+H]$^+$): 218.1743, measured: 245.2262.

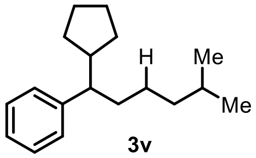

3v

(1-cyclopentyl-5-methylhexyl)benzene (3v): The reaction was conducted following the general procedure A in a 0.5 mmol scale. The residue was purified by column chromatography on silica gel to afford the product **3v** (48.9 mg, 40 % yield, *rr* = 6/1) as a colorless oil. ^{1}H NMR (400 MHz, Chloroform-*d*) δ 7.21 - 7.17 (m, 2 H), 7.11 - 7.04 (m, 3 H), 2.19 - 2.13 (m, 1 H), 1.93 - 1.82 (m, 2 H), 1.69 - 1.56 (m, 2 H), 1.49 - 1.42 (m, 3 H), 1.37 - 1.29 (m, 2 H), 1.26 - 1.13 (m, 2 H), 1.06 - 0.94 (m, 4 H), 0.91 - 0.83 (m, 1 H), 0.70 (dd, *J* = 8.4, 6.6 Hz, 6 H) ppm; ^{13}C NMR (101 MHz, Chloroform-*d*) δ 146.2, 128.2, 128.1, 125.7, 52.4, 46.9, 39.2, 35.6, 31.9, 31.7, 27.9, 25.4, 25.4, 25.0, 23.0, 22.5 ppm; HRMS (ESI) Calculated for $C_{18}H_{29}$ ([M+H]$^+$): 218.1743, measured: 245.2262.

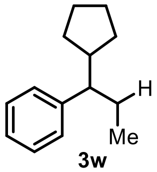

3w

(1-cyclopentylpropyl)benzene (3w) [3]: The reaction was conducted following the general procedure **A** in a 0.5 mmol scale. The residue was purified by column chromatography on silica gel to afford the product **3w** (26.1 mg, 44% yield, *rr* = 17/1) as a colorless oil. ^{1}H NMR (400 MHz, Chloroform-*d*) δ 7.28 - 7.25 (m, 2 H), 7.18 - 7.16 (m, 1 H), 7.15 - 7.11 (m, 2 H), 2.15 (td, *J* = 10.3, 3.6 Hz, 1 H), 2.05 - 1.78 (m, 3 H), 1.66 - 1.55 (m, 2 H), 1.54 - 1.28 (m, 4 H), 1.26 - 1.15 (m, 1 H), 1.00 - 0.90 (m, 1 H), 0.68 (t, *J* = 7.4 Hz, 3 H) ppm. ^{13}C NMR (101 MHz, Chloroform-*d*) δ 145.7, 128.2, 128.0, 125.6, 54.2, 46.5, 31.9, 31.6, 28.1, 25.3, 24.9, 12.2 ppm.

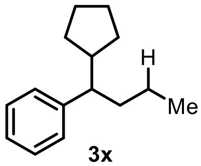

3x

(1-cyclopentylbutyl)benzene (3x) [3]: The reaction was conducted following the general procedure A in a 0.5 mmol scale. The residue was purified by column chromatography on silica gel to afford the product **3x** (63.7 mg, 63% yield, *rr* = 7/1) as a colorless oil. ^{1}H NMR (400 MHz, Chloroform-*d*) δ 7.28 - 7.23 (m, 2 H), 7.19 - 7.11 (m, 3 H), 2.28 -2.22 (m, 1 H), 2.03 - 1.88 (m, 2 H), 1.76 - 1.56 (m, 3 H), 1.54 - 1.45 (m, 2 H), 1.42 - 1.26 (m, 2 H), 1.24 - 1.13 (m, 1 H), 1.11 - 0.99 (m, 2 H), 0.98 - 0.89 (m, 1 H), 0.81 (t, *J* = 7.3 Hz, 3 H) ppm; ^{13}C NMR (101 MHz, Chloroform-*d*) δ 146.1, 128.2, 128.1, 125.7, 52.2, 46.9, 37.7, 32.0, 31.8, 25.4, 25.0, 20.8, 14.3 ppm.

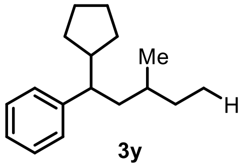

3y

(1-cyclopentyl-3-methylpentyl)benzene (3y): The reaction was conducted following the general procedure **A** in a 0.5 mmol scale. The residue was purified by column chromatography on silica gel to afford the product **3y** (49.5 mg, 43% yield, *rr* = 8/1, dr = 1/1) as a colorless oil.^{1}H NMR (400 MHz, Chloroform-*d*) δ 7.32 - 7.28 (m, 2 H), 7.23 - 7.15 (m, 3 H), 2.44 - 2.36 (m, 1 H), 2.02 - 1.90 (m, 2 H), 1.75 - 1.62 (m, 2 H), 1.58 - 1.52 (m, 2 H), 1.50 - 1.40 (m, 2 H), 1.36 - 1.22 (m, 2 H), 1.21 - 1.09 (m, 2 H), 1.06 - 0.92 (m, 2 H), 0.86 - 0.76 (m, 6 H) ppm; ^{13}C NMR (101 MHz, Chloroform- *d*) δ 146.3, 146.0, 128.17, 128.16, 128.1, 125.7, 49.79, 49.77, 47.7, 47.5, 42.5, 42.2, 32.0, 31.9, 31.78, 31.76, 31.7,

31.4, 31.0, 27.5, 25.4, 25.04, 25.02, 20.5, 18.6, 11.5, 10.7. HRMS (ESI) Calculated for $C_{17}H_{26}Na$ ($[M+Na]^+$): 253.1926, measured: 253.1911.

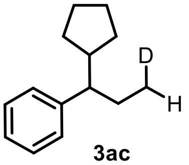

3ac

(1-cyclopentylpropyl-3-d)benzene (3ac): The reaction was conducted following the general procedure **A** in a 0.5 mmol scale. The residue was purified by column chromatography on silica gel to afford the product **3ac** (46.4 mg, 49% yield, 95% D, *rr* = 15/1) as a colorless oil. ^{1}H NMR (400 MHz, Chloroform-*d*) δ 7.32 - 7.29 (m, 2 H), 7.23 - 7.20 (m, 1 H), 7.19 - 7.16 (m, 2 H), 2.19 (td, *J* = 10.3, 3.6 Hz, 1 H), 2.09 - 1.93 (m, 2 H), 1.90 - 1.82 (m, 1 H), 1.73 - 1.63 (m, 1 H), 1.58 - 1.52 (m, 2 H), 1.51 - 1.28 (m, 3 H), 1.28 - 1.18 (m, 1 H), 1.04 - 0.94 (m, 1 H), 0.74 - 0.68 (m, 2 H) ppm; ^{13}C NMR (101 MHz, Chloroform-*d*) δ 145.8, 128.3, 128.1, 125.8, 54.3, 46.6, 32.0, 31.7, 28.1, 25.4, 25.0, 12.0 (t, *J* = 19.2 Hz) ppm; HRMS (ESI) Calculated for $C_{14}H_{20}D$ ($[M+H]^+$): 290.1714, measured: 290.1700.

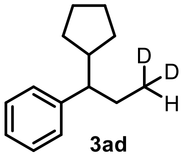

3ad

(1-cyclopentylpropyl-3,3-d2)benzene (3ad): The reaction was conducted following the general procedure **A** in a 0.5 mmol scale. The residue was purified by column chromatography on silica gel with petroleum ether to afford the product **3ad** (70.4 mg, 74% yield, 94% D, *rr* = 21/1) as a colorless oil. ^{1}H NMR (400 MHz, Chloroform-*d*) δ 7.32 - 7.26 (m, 2 H), 7.23 - 7.13 (m, 3 H), 2.18 (td, *J* = 10.3, 3.6 Hz, 1 H), 2.08 - 1.90 (m, 2 H), 1.88 - 1.82 (m, 1 H), 1.71 - 1.62 (m, 1 H), 1.60 - 1.49 (m, 3 H), 1.48 - 1.31 (m, 2 H), 1.29 - 1.18 (m, 1 H), 1.05 - 0.94 (m, 1 H), 0.72 - 0.65 (m, 1 H) ppm; ^{13}C NMR (101 MHz, Chloroform-*d*) δ 145.8, 128.3, 128.1, 125.8, 54.3, 46.6, 32.0, 31.7, 28.0, 25.4, 25.0, 11.7 (p, *J* = 38.2, 19.1 Hz) ppm; HRMS (ESI) Calculated for $C_{14}H_{19}D_2$ ($[M+H]^+$): 191.1762, measured: 191.1763.

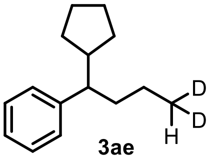

3ae

(1-cyclopentylbutyl-4,4-d2)benzene (3ae): The reaction was conducted following the general procedure A in a 0.5 mmol scale. The residue was purified by column chromatography on silica gel to afford the product **3ae** (43.9 mg, 43% yield, 93% D, *rr* = 22/1) as a colorless oil. ^{1}H NMR (400 MHz, Chloroform-*d*) δ 7.28 - 7.24 (m, 2 H), 7.18 - 7.11 (m, 3 H), 2.25 (td, *J* = 10.3, 3.2 Hz, 1 H), 2.03 - 1.88 (m, 2 H), 1.76 - 1.67 (m, 1 H), 1.65 - 1.46 (m, 4 H), 1.44 - 1.27 (m, 2 H), 1.24 - 1.15 (m, 1 H), 1.12 - 0.91 (m, 3 H), 0.81 - 0.75 (m, 1 H) ppm; ^{13}C NMR (101 MHz, Chloroform-*d*) δ 146.2, 128.2, 128.1, 125.7, 52.3, 46.9, 37.6, 32.0, 31.8, 25.4, 25.0, 20.7, 13.74 (p, *J* = 19.0 Hz) ppm; HRMS (ESI) Calculated for $C_{15}H_{20}D_2$ ($[M+Na]^+$): 227.1739, measured: 227.1705.

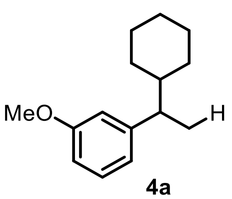
4a

1-(1-cyclohexylethyl)-3-methoxybenzene (4a): The reaction was conducted following the general procedure **A** in a 0.5 mmol scale. The residue was purified by column chromatography on silica gel to afford the product **4a** (69.9 mg, 64% yield, *rr* = 10/1) as a colorless oil. ^{1}H NMR (400 MHz, Chloroform-*d*) δ 7.20 (t, *J* = 7.8 Hz, 1 H), 6.76 - 6.70 (m, 3 H), 3.81 (s, 3 H), 2.44 - 2.37 (m, 1 H), 1.90 - 1.86 (m, 1 H), 1.77 - 1.72 (m, 1 H), 1.64 - 1.59 (m, 3 H), 1.45 - 1.34 (m, 2 H), 1.22 (d, *J* = 7.1 Hz, 3 H), 1.14 - 1.07 (m, 2 H), 1.00 - 0.79 (m, 2 H) ppm; ^{13}C NMR (101 MHz, Chloroform-*d*) δ 159.5, 149.1, 129.0, 120.4, 113.9, 110.5, 55.2, 46.2, 44.2, 33.4, 31.6, 30.7, 26.7, 26.6, 19.0 ppm; HRMS (ESI) Calculated for $C_{15}H_{22}O$ ($[M+H]^+$): 218.1743, measured: 218.1723.

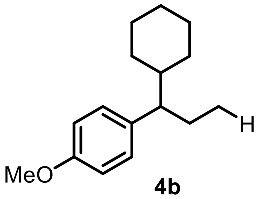
4b

1-(1-cyclohexylpropyl)-4-methoxybenzene (4b) [3]: The reaction was conducted following the general procedure **A** in a 0.5 mmol scale. The residue was purified by column chromatography on silica gel to afford the product **4b** (52.3 mg, 45% yield, *rr* = 12/1) as a colorless oil. ^{1}H NMR (400 MHz, Chloroform-*d*) δ 7.05 - 7.02 (m, 2 H), 6.87 - 6.84 (m, 2 H), 3.83 (s, 3 H), 2.20 - 2.15 (m, 1 H), 1.93 - 1.81 (m, 2 H), 1.78 - 1.72 (m, 1 H), 1.56 - 1.39 (m, 4 H), 1.31 - 1.04 (m, 4 H), 0.97 - 0.87 (m, 1 H), 0.83 - 0.76 (m, 1 H), 0.72 (t, *J* = 7.3 Hz, 3 H) ppm; ^{13}C NMR (101 MHz, Chloroform-*d*) δ 157.6, 136.7, 129.5, 113.3, 55.3, 53.3, 43.2, 31.6, 31.1, 26.80, 26.78, 26.7, 25.5, 12.6 ppm.

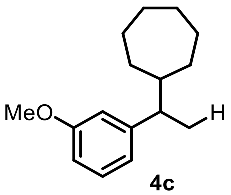
4c

(1-(3-methoxyphenyl)ethyl)cycloheptane (4c) [3]: The reaction was conducted following the general procedure **A** in a 0.5 mmol scale. The residue was purified by column chromatography on silica gel to afford the product **4c** (73.2 mg, 63% yield, *rr* = 20/1) as a colorless oil. ^{1}H NMR (400 MHz, Chloroform-*d*) δ 7.21 (t, *J* = 8.1 Hz, 1 H), 6.79 (d, *J* = 7.8 Hz, 1 H), 6.74 - 6.72 (m, 2 H), 3.81 (s, 3 H), 2.62 - 2.54 (m, 1 H), 1.82 - 1.75 (m, 1 H), 1.71 - 1.62 (m, 2 H), 1.61 - 1.53 (m, 4 H), 1.50 - 1.40 (m, 3 H), 1.40 - 1.28 (m, 2 H), 1.22 (d, *J* = 7.0 Hz, 3 H), 1.19 - 1.11 (m, 1 H) ppm; ^{13}C NMR (101 MHz, Chloroform-*d*) δ 159.5, 149.2, 129.0, 120.4, 113.9, 110.5, 55.2, 46.0, 45.5, 32.7, 31.3, 28.6, 28.4, 26.9, 26.7, 18.5 ppm.

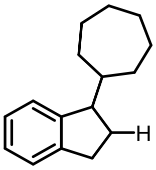
4d

1-cycloheptyl-2,3-dihydro-1H-indene (4d) [3]: The reaction was conducted following the general procedure **A** in a 0.5 mmol scale. The residue was purified by column chromatography on silica gel to afford the product **4d** (65.4 mg, 61% yield, *rr* = 11/1) as a colorless oil. ^{1}H NMR (400 MHz, Chloroform-*d*) δ 7.25 - 7.10 (m, 4 H), 3.27 - 3.18 (m, 1 H), 2.97 - 2.75 (m, 2 H), 2.18 - 2.07 (m, 1 H), 2.07 - 1.97 (m, 1 H), 1.92 - 1.81 (m, 1 H), 1.80 - 1.72 (m, 2 H), 1.69 - 1.59 (m, 2 H), 1.59 - 1.49 (m, 3 H), 1.48 - 1.27

(m, 4 H), 1.26 - 1.16 (m, 1 H) ppm; ^{13}C NMR (101 MHz, $CDCl_3$ Chloroform-*d*) δ 146.6, 144.9, 126.2, 126.0, 124.4, 124.0, 52.0, 42.3, 34.2, 31.9, 29.3, 28.6, 27.8, 27.7, 27.2 ppm.

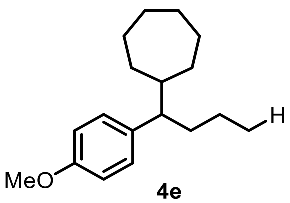

4e

(1-(4-methoxyphenyl)butyl)cycloheptane (4e): The reaction was conducted following the general procedure **A** in a 0.5 mmol scale. The residue was purified by column chromatography on silica gel to afford the product **4e** (65.1 mg, 50% yield, *rr* = 14/1) as a colorless oil. 1H NMR (400 MHz, Chloroform-d) δ 7.07 (d, J = 8.6 Hz, 2 H), 6.85 (d, J = 8.6 Hz, 2 H), 3.83 (s, 3 H), 2.39 - 2.34 (m, 1 H), 1.81 - 1.75 (m, 1 H), 1.70 - 1.59 (m, 3 H), 1.57 - 1.50 (m, 4 H), 1.45 - 1.37 (m, 3 H), 1.34 - 1.02 (m, 6 H), 0.86 (t, J = 7.3 Hz, 3 H) ppm; ^{13}C NMR (101 MHz, Chloroform-*d*) δ 157.6, 137.1, 129.5, 113.3, 55.3, 51.2, 44.9, 35.3, 32.6, 31.8, 28.6, 28.3, 27.0, 26.9, 21.2, 14.4 ppm; HRMS (ESI) Calculated for $C_{18}H_{28}ONa$ ($[M+Na]^+$): 283.2032, measured: 283.2033.

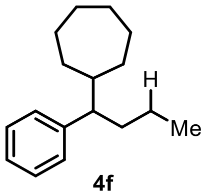

4f

(1-phenylbutyl)cycloheptane (4f): The reaction was conducted following the general procedure A in a 0.5 mmol scale. The residue was purified by column chromatography on silica gel to afford the product **4f** (56.4 mg, 49 % yield, *rr* = 3/1) as a colorless oil. ^{1}H NMR (400 MHz, Chloroform-*d*) δ 7.27 - 7.23 (m, 2 H), 7.19 - 7.15 (m, 1 H), 7.14 - 7.11 (m, 2 H), 2.45 - 2.37 (m, 1 H), 1.85 - 1.78 (m, 1 H), 1.73 - 1.61 (m, 3 H), 1.61 - 1.54 (m, 3 H), 1.46 - 1.35 (m, 4 H), 1.35 - 1.14 (m, 3 H), 1.13 - 0.99 (m, 3 H), 0.82 (t, *J* = 7.3 Hz, 3 H) ppm; ^{13}C NMR (101 MHz, Chloroform-*d*) δ 145.2, 128.8, 128.0, 125.7, 52.2, 44.8, 35.2, 32.5, 32.0, 28.6, 28.3, 26.9, 26.8, 21.2, 14.4 ppm; HRMS (ESI) Calculated for $C_{17}H_{27}$ ($[M+H]^+$): 231.2107, measured: 231.2101.

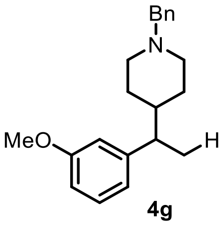

4g

1-benzyl-4-(1-(3-methoxyphenyl)ethyl)piperidine (4g): The reaction was conducted following the general procedure A in a 0.5 mmol scale. The residue was purified by column chromatography on silica gel to afford the product **4g** (54.2 mg, 35 % yield, *rr* = 4/1) as a colorless oil. ^{1}H NMR (400 MHz, Chloroform-*d*) δ 7.32 - 7.17 (m, 6 H), 6.78 - 6.67 (m, 3 H), 3.78 (s, 3 H), 3.45 (d, *J* = 1.82 Hz, 2 H), 2.96 - 2.74 (m, 2 H), 2.45 - 2.35 (m, 1 H), 1.97 - 1.74 (m, 3 H), 1.38 - 1.25 (m, 3 H), 1.22 (d, *J* = 6.98 Hz, 3 H), 1.20 - 1.12 (m, 1 H) ppm; ^{13}C NMR (101 MHz, Chloroform-*d*) δ 159.5, 148.5, 138.5, 129.4, 129.2, 128.2, 127.0, 120.2, 113.7, 110.7, 63.5, 55.2, 54.2, 54.1, 45.7, 42.5, 30.9, 30.2, 19.1 ppm; HRMS (ESI) Calculated for $C_{21}H_{27}NONa$ ($[M+Na]^+$): 332.2005, measured: 332.2011.

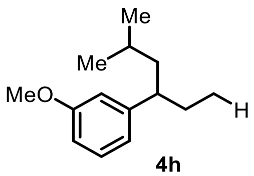

4h

1-methoxy-3-(5-methylhexan-3-yl)benzene (4h): The reaction was conducted following the general procedure A in a 0.5 mmol scale. The residue was purified by column chromatography on silica gel to afford the product **4h** (25.8 mg, 25 % yield, *rr* = 6/1) as a colorless oil. ^{1}H NMR (400 MHz, Chloroform-*d*) δ 7.23 - 7.14 (m, 1 H), 6.91 - 6.49 (m, 3 H), 3.81 (s, 3 H), 2.50 - 2.34 (m, 1 H), 1.67 - 1.44 (m, 3 H), 1.43 - 1.26 (m, 2 H), 0.83 (dd, *J* = 14.68, 5.83 Hz, 6 H), 0.76 (t, *J* = 7.27 Hz, 3 H) ppm; ^{13}C NMR (101 MHz, Chloroform-*d*) δ 159.6, 148.0, 129.2, 120.5, 113.9, 110.6, 55.2, 46.0, 45.6, 30.3, 25.5, 23.7, 22.0, 12.4 ppm; HRMS (ESI) Calculated for $C_{14}H_{22}O$ ([M+H]$^+$): 207.1752, measured: 207.1743.

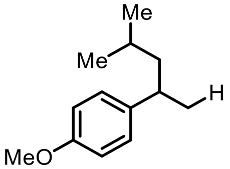

4i

1-methoxy-4-(4-methylpentan-2-yl)benzene (4i): The reaction was conducted following the general procedure A in a 0.5 mmol scale. The residue was purified by column chromatography on silica gel to afford the product **4i** (30.0 mg, 27 % yield, *rr* = 7/1) as a colorless oil. ^{1}H NMR (400 MHz, Chloroform-*d*) δ 7.13 - 7.08 (m, 2 H), 6.86 - 6.82 (m, 2 H), 3.79 (s, 3 H), 2.78 - 2.69 (m, 1 H), 1.51 - 1.31 (m, 3 H), 1.18 (d, *J* = 6.90 Hz, 3 H), 0.85 (dd, *J* = 11.48, 6.22 Hz, 6 H) ppm; ^{13}C NMR (101 MHz, Chloroform-*d*) δ 157.7, 140.2, 127.9, 113.8, 55.4, 48.1, 36.8, 25.7, 23.2, 22.5 ppm; HRMS (ESI) Calculated for $C_{13}H_{20}O$ ([M+Na]$^+$): 215.1406, measured: 215.1403.

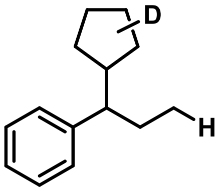

3ah

(1-(cyclopentyl-d)propyl)benzene (3ah): The reaction was conducted following the general procedure A in a 0.5 mmol scale. The residue was purified by column chromatography on silica gel to afford the product **3ah** (61.5 mg, 65 % yield, *rr* = 21/1) as a colorless oil. ^{1}H NMR (400 MHz, Chloroform-*d*) δ 7.21 - 7.17 (m, 2 H), 7.11 - 7.04 (m, 3 H), 2.11 - 2.05 (m, 1 H), 1.95 - 1.72 (m, 3 H), 1.55 - 1.52 (m, 1 H), 1.47 - 1.42 (m, 2 H), 1.35 - 1.21 (m, 2 H), 1.20 - 1.07 (m, 2 H), 0.90 - 0.83 (m, 1 H), 0.61 (t, *J* = 7.36 Hz, 3 H) ppm; ^{13}C NMR (101 MHz, Chloroform-*d*) δ 145.8, 128.3, 128.1, 125.8, 54.3, 46.6 (d, *J* = 9.06 Hz), 31.9 (d, *J* = 13.16 Hz), 31.7 (d, *J* = 9.91 Hz), 28.2, 25.4 (d, *J* = 9.98 Hz), 25.0 (d, *J* = 10.04 Hz), 12.3 ppm.

6. References

1. Bejot, R.; Tisserand, S.; Li, D. R.; Falck, J. R.; Mioskowskia, C. *Tetrahedron Lett.* **2014**, *48*, 3855.

2. Stokes, B. J.; Bischoff, A. J.; Sigman, M. S. *Chem. Sci.* **2014**, *5,* 2336.

3. Jörg T. Binder,Christopher J. Cordier, and Gregory C. Fu. *J. Am. Chem. Soc.* **2012**, *134*, 17003.

7. NMR Spectra

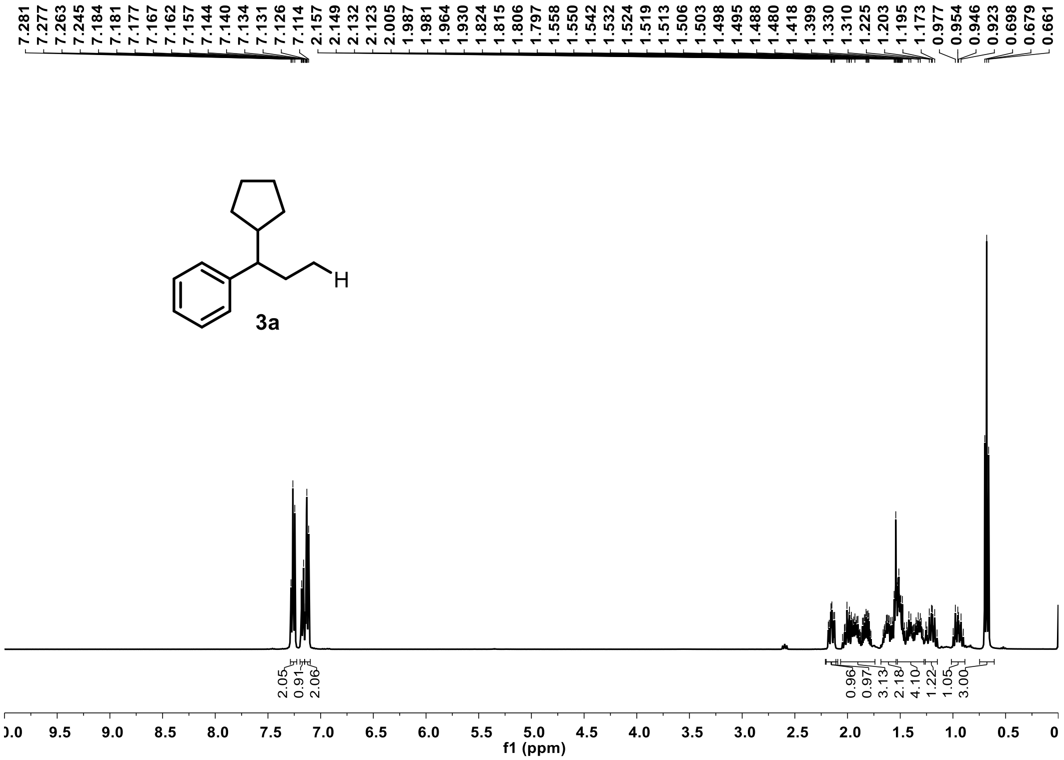

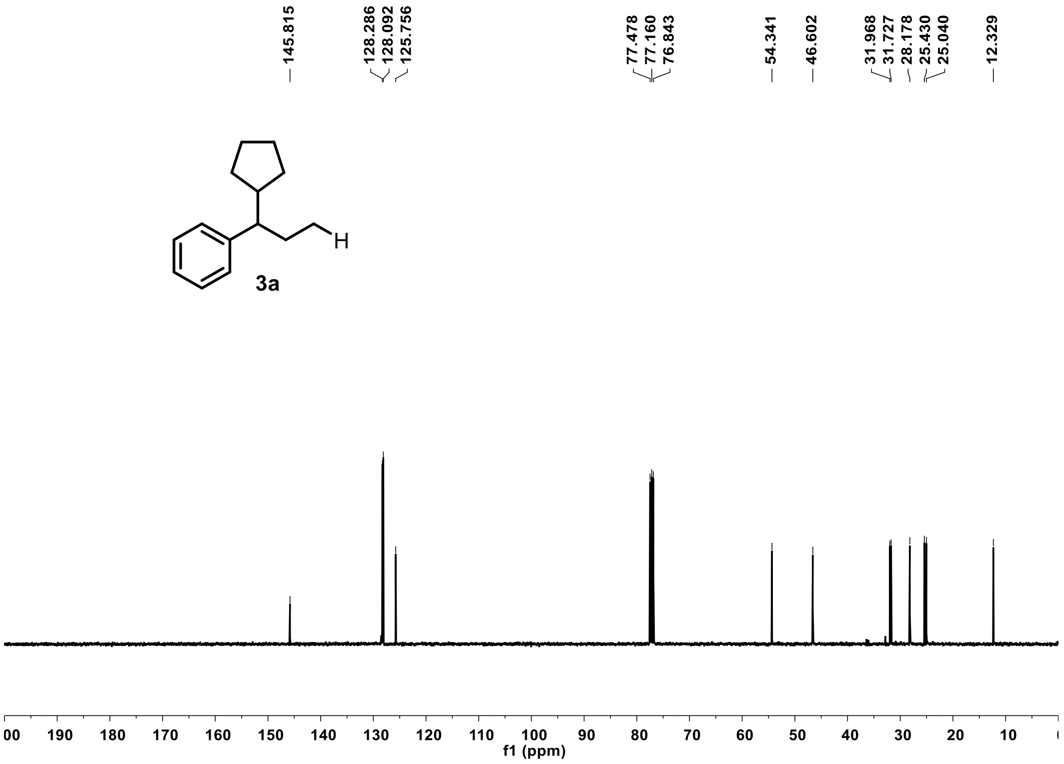

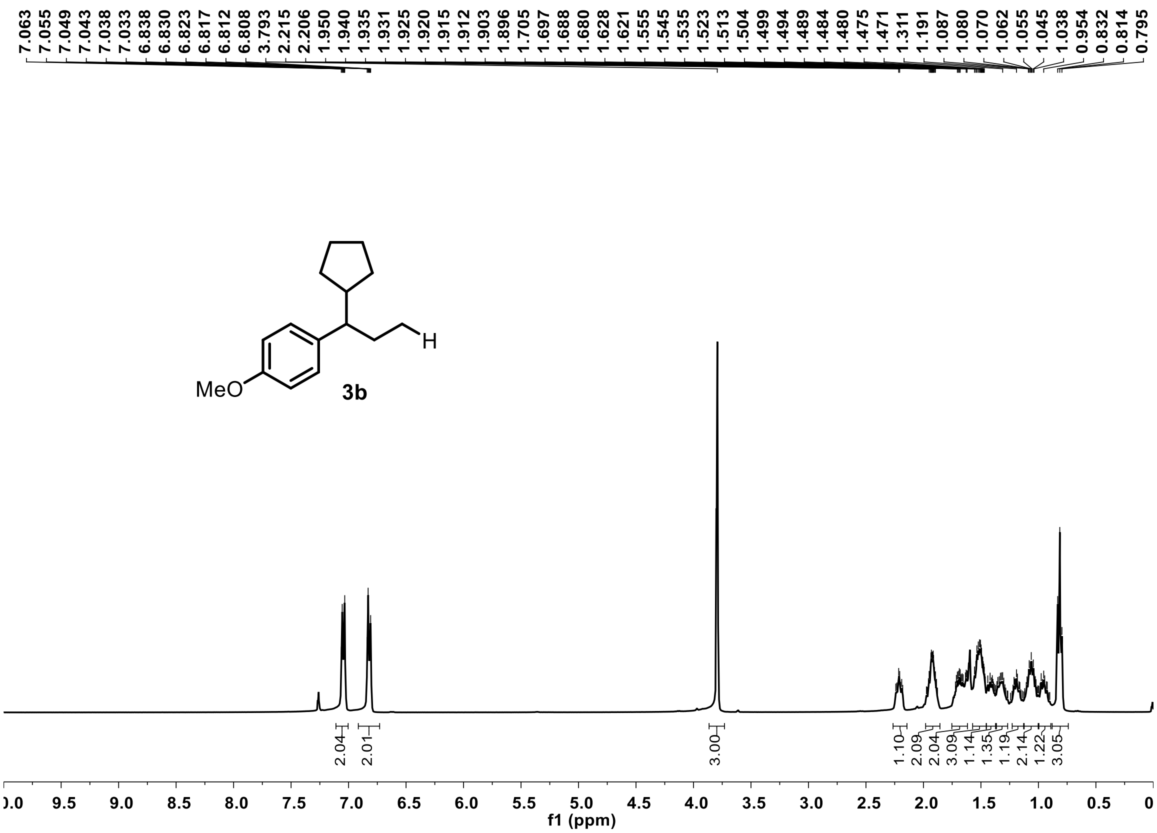
7.063 7.055 7.049 7.043 7.038 7.033 6.838 6.830 6.823 6.817 6.812 6.808 3.793 2.215 2.206 1.950 1.940 1.935 1.931 1.925 1.920 1.915 1.912 1.903 1.896 1.705 1.697 1.688 1.680 1.628 1.621 1.555 1.545 1.535 1.523 1.513 1.504 1.499 1.494 1.489 1.484 1.480 1.475 1.471 1.311 1.191 1.087 1.080 1.070 1.062 1.055 1.045 1.038 0.954 0.832 0.814 0.795
H
MeO
3b
2.04 2.01 3.00 1.10 2.09 2.04 3.09 1.14 1.35 1.19 2.14 1.22 3.05
f1 (ppm)

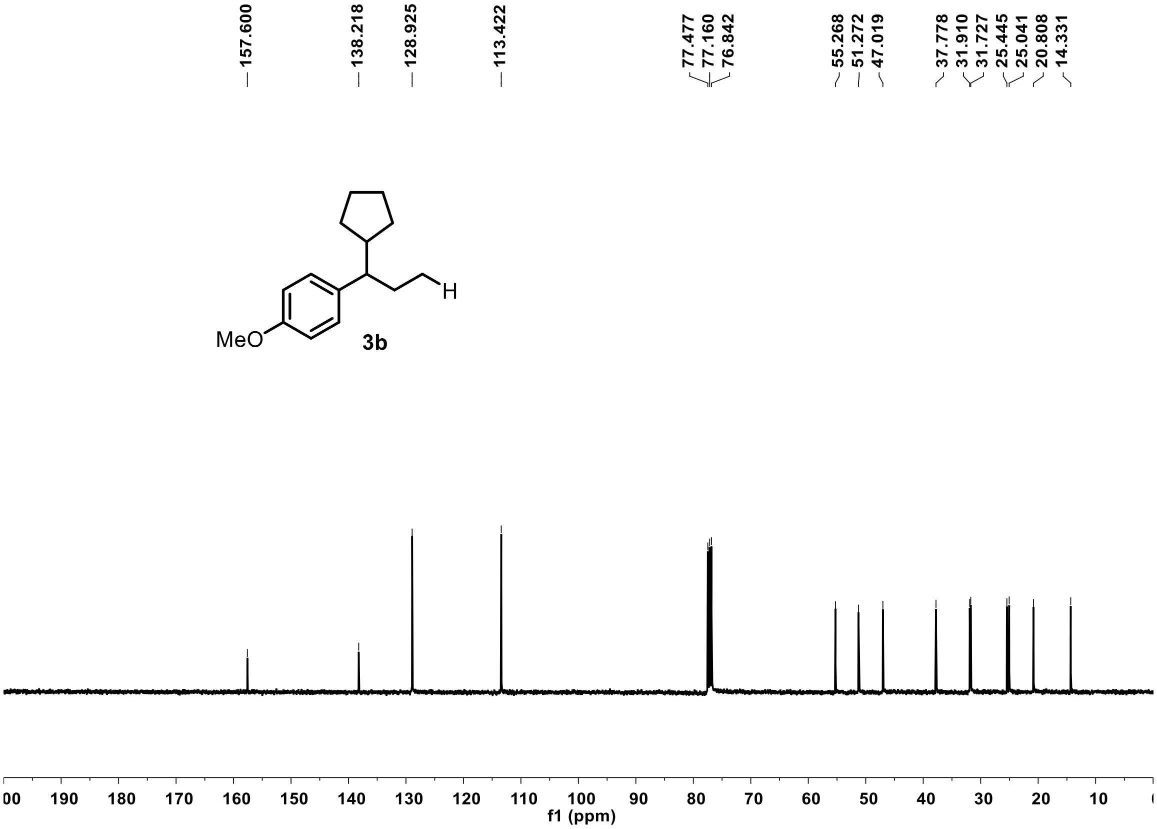
157.600 138.218 128.925 113.422 77.477 77.160 76.842 55.268 51.272 47.019 37.778 31.910 31.727 25.445 25.041 20.808 14.331
H
MeO
3b
f1 (ppm)

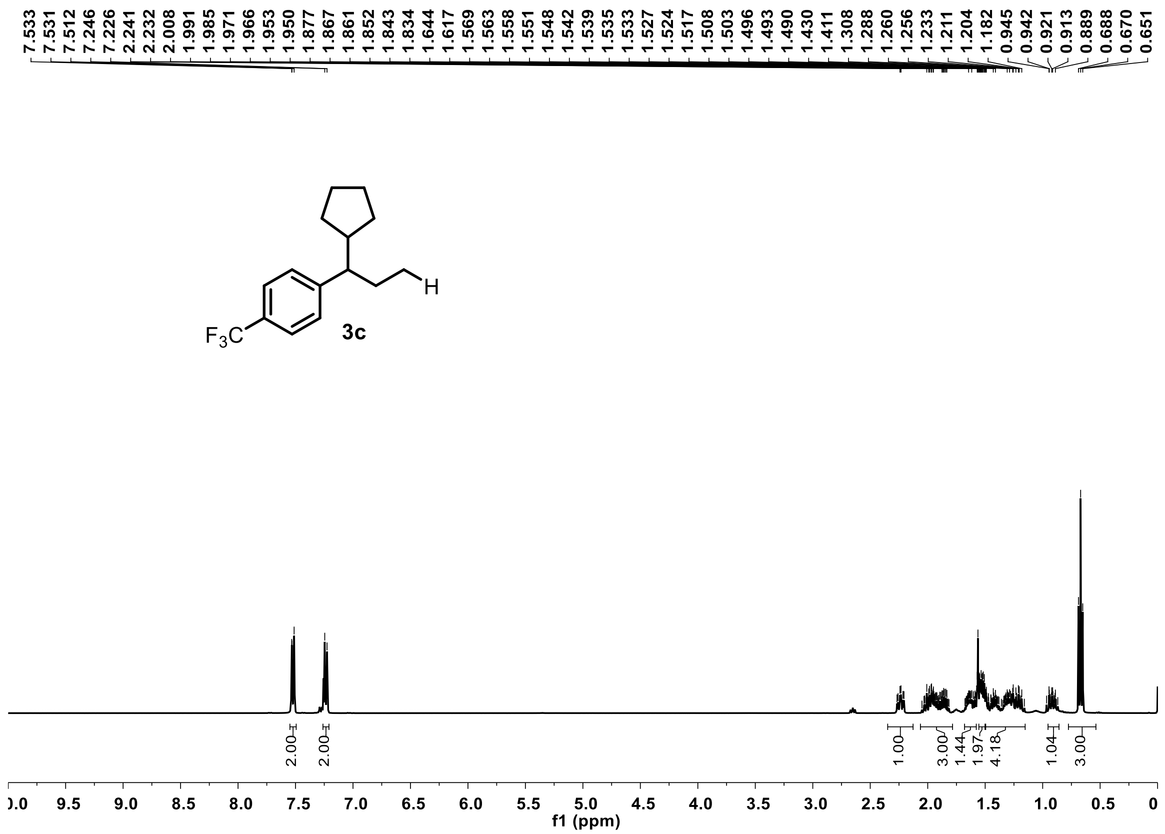
7.533 7.531 7.512 7.246 7.226 2.241 2.232 2.008 1.991 1.985 1.971 1.966 1.953 1.950 1.877 1.867 1.861 1.852 1.843 1.834 1.644 1.617 1.569 1.563 1.558 1.551 1.548 1.542 1.539 1.535 1.533 1.527 1.524 1.517 1.508 1.503 1.496 1.493 1.490 1.430 1.411 1.308 1.288 1.260 1.256 1.233 1.211 1.204 1.182 0.945 0.942 0.921 0.913 0.889 0.688 0.670 0.651
H
F3C
3c
2.00 2.00 1.00 3.00 1.44 1.97 4.18 1.04 3.00
9.5 9.0 8.5 8.0 7.5 7.0 6.5 6.0 5.5 5.0 4.5 4.0 3.5 3.0 2.5 2.0 1.5 1.0 0.5 0
f1 (ppm)

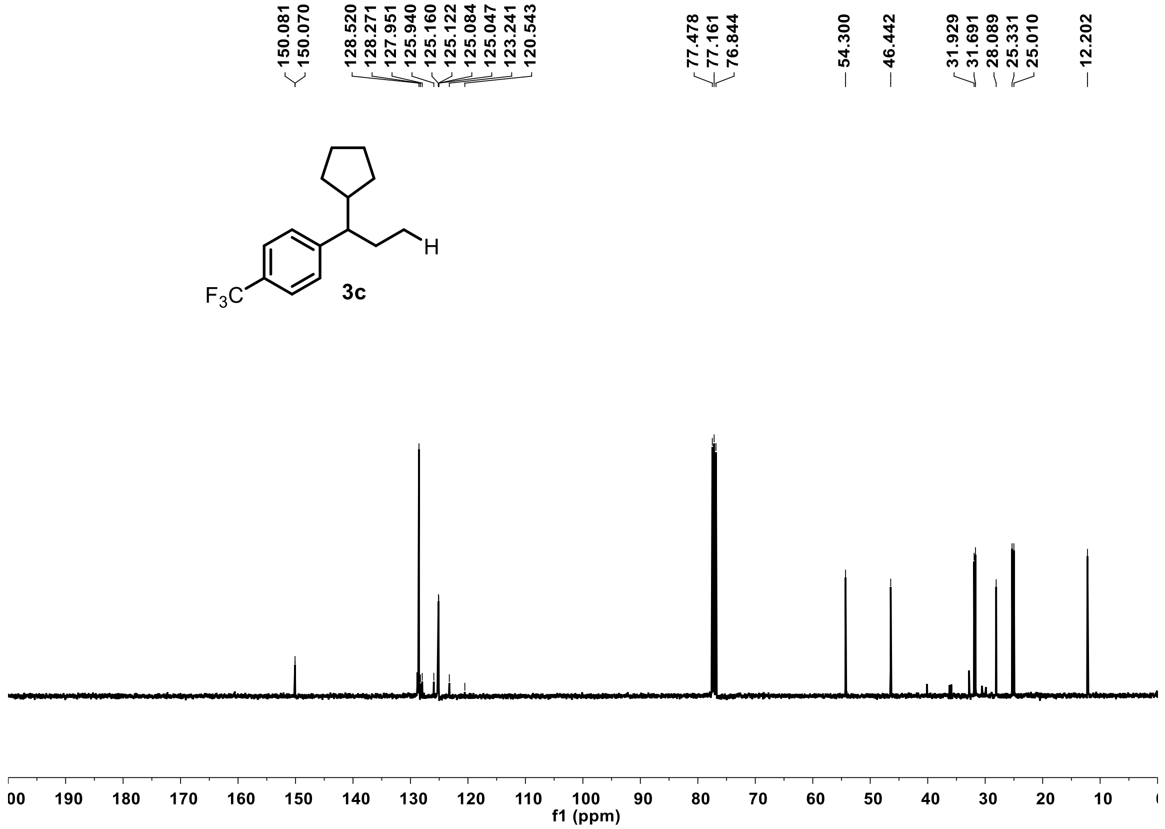
150.081 150.070 128.520 128.271 127.951 125.940 125.160 125.122 125.084 125.047 123.241 120.543 77.478 77.161 76.844 54.300 46.442 31.929 31.691 28.089 25.331 25.010 12.202
H
F3C
3c
190 180 170 160 150 140 130 120 110 100 90 80 70 60 50 40 30 20 10
f1 (ppm)

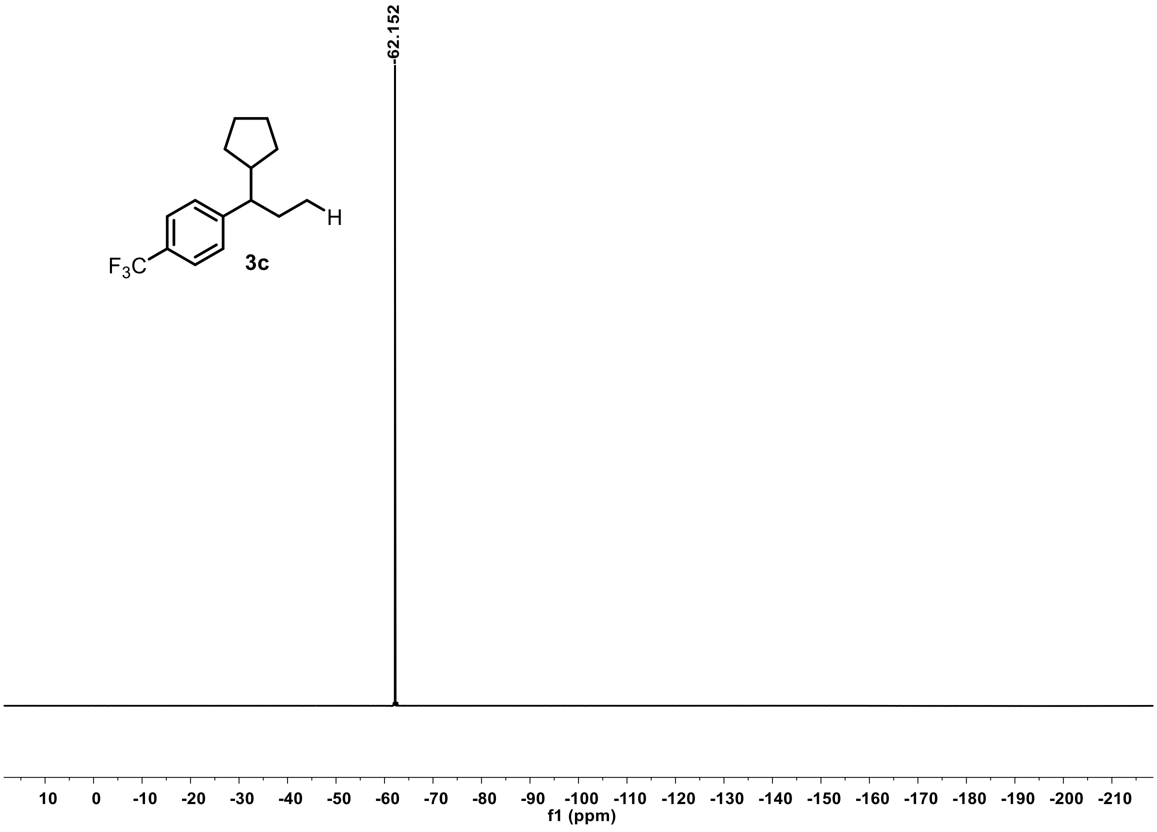
-62.152
F3C
H
3c
10 0 -10 -20 -30 -40 -50 -60 -70 -80 -90 -100 -110 -120 -130 -140 -150 -160 -170 -180 -190 -200 -210
f1 (ppm)

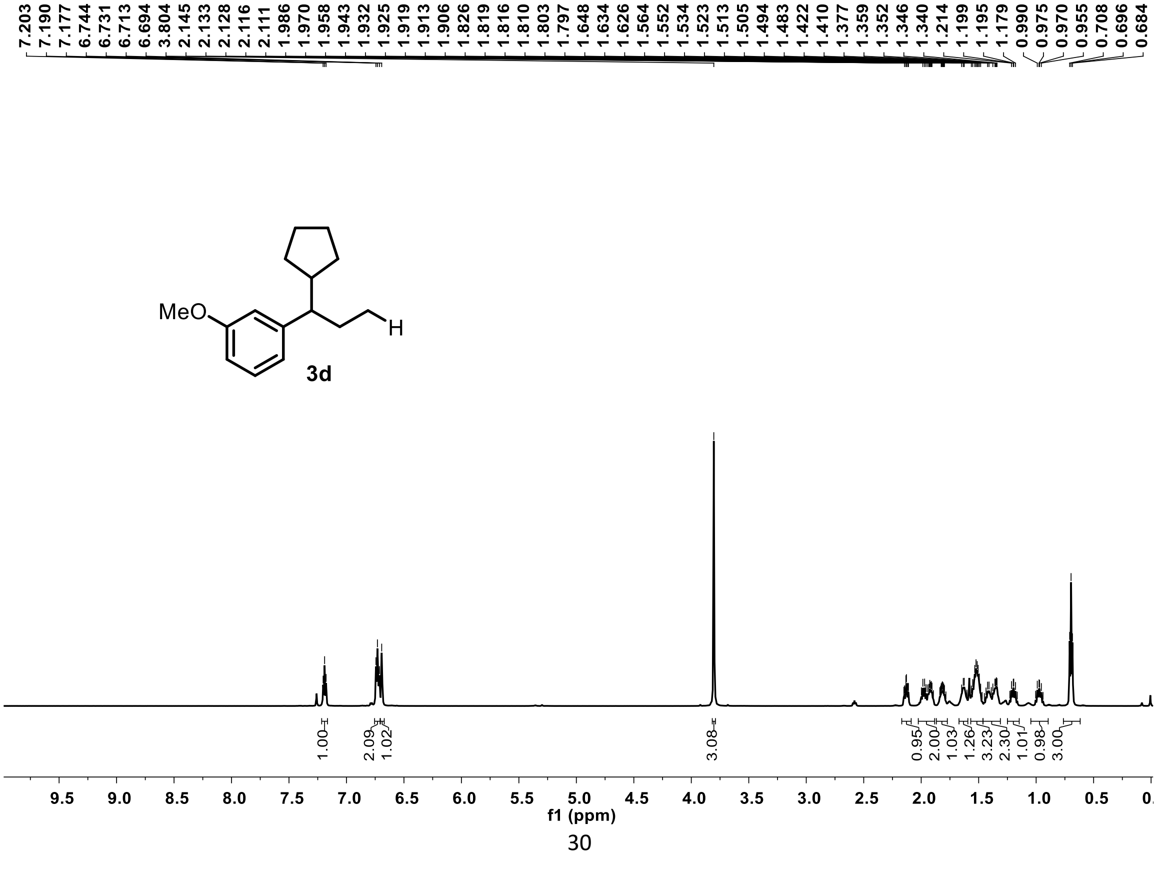
7.203 7.190 7.177 6.744 6.731 6.713 6.694 3.804 2.145 2.133 2.128 2.116 2.111 1.986 1.970 1.958 1.943 1.932 1.925 1.919 1.913 1.906 1.826 1.819 1.816 1.810 1.803 1.797 1.648 1.634 1.626 1.564 1.552 1.534 1.523 1.513 1.505 1.494 1.483 1.422 1.410 1.377 1.359 1.352 1.346 1.340 1.214 1.199 1.195 1.179 0.990 0.975 0.970 0.955 0.708 0.696 0.684
MeO
H
3d
1.00
2.09
1.02
3.08
0.95
2.00
1.03
1.26
3.23
2.30
1.01
0.98
3.00
9.5 9.0 8.5 8.0 7.5 7.0 6.5 6.0 5.5 5.0 4.5 4.0 3.5 3.0 2.5 2.0 1.5 1.0 0.5 0.
f1 (ppm)

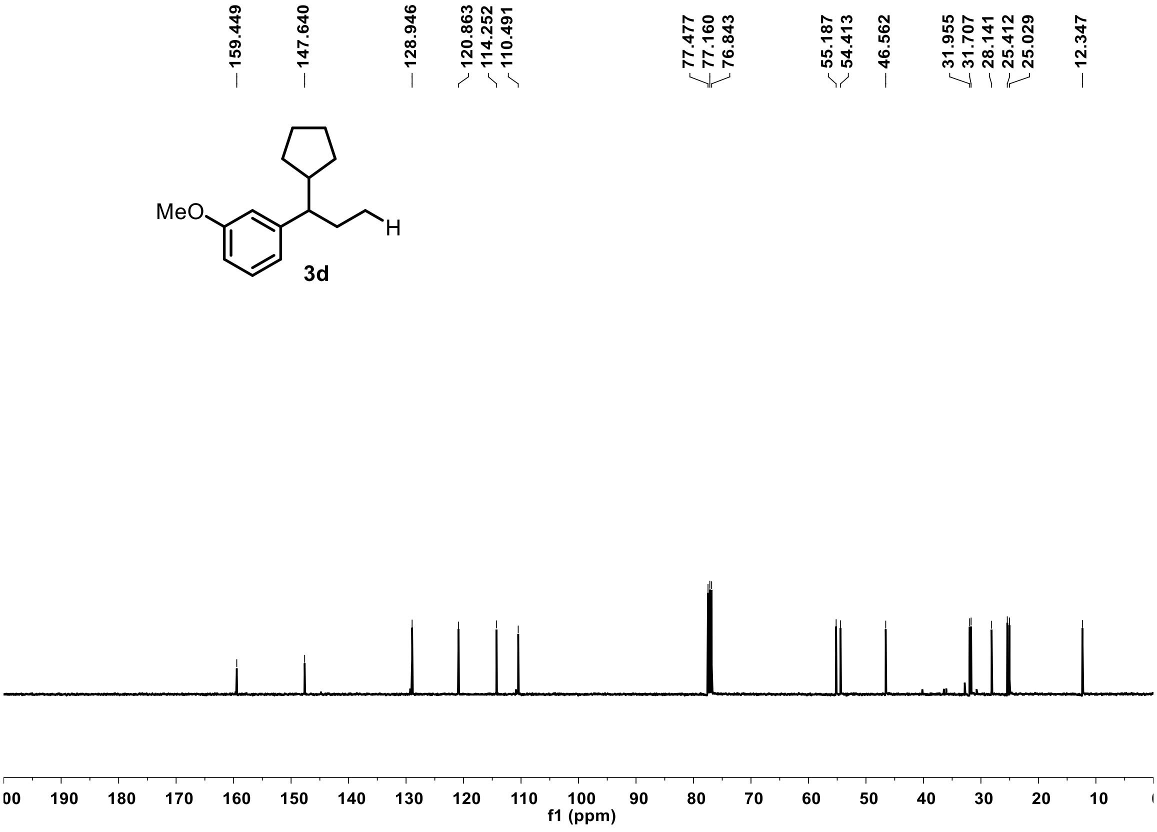
159.449
147.640
128.946
120.863
114.252
110.491
77.477
77.160
76.843
55.187
54.413
46.562
31.955
31.707
28.141
25.412
25.029
12.347
MeO
H
3d
00 190 180 170 160 150 140 130 120 110 100 90 80 70 60 50 40 30 20 10
f1 (ppm)

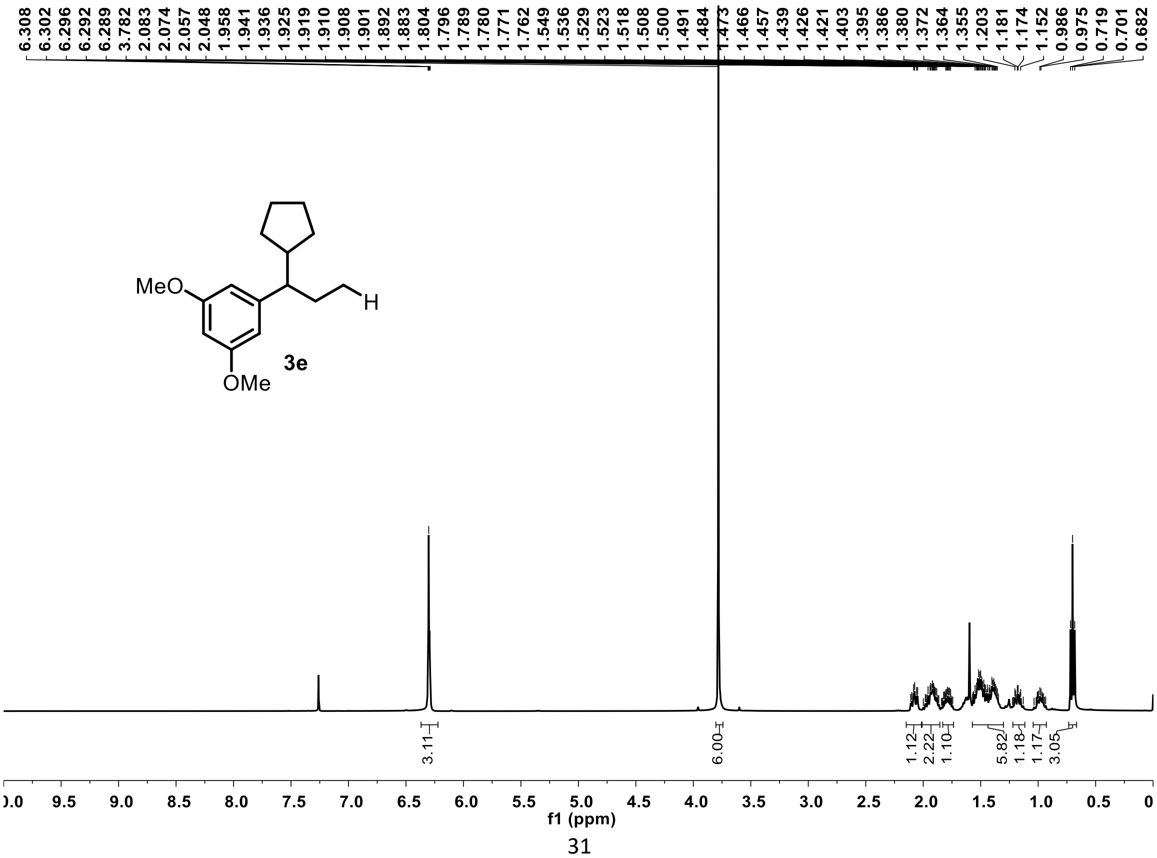
6.308
6.302
6.296
6.292
6.289
3.782
2.083
2.074
2.057
2.048
1.958
1.941
1.936
1.925
1.919
1.910
1.908
1.901
1.892
1.883
1.804
1.796
1.789
1.780
1.771
1.762
1.549
1.536
1.529
1.523
1.518
1.508
1.500
1.491
1.484
1.473
1.466
1.457
1.439
1.426
1.421
1.403
1.395
1.386
1.380
1.372
1.364
1.355
1.203
1.181
1.174
1.152
0.986
0.975
0.719
0.701
0.682
MeO
H
3e
OMe
3.11
6.00
1.12
2.22
1.10
5.82
1.18
1.17
3.05
0.0 9.5 9.0 8.5 8.0 7.5 7.0 6.5 6.0 5.5 5.0 4.5 4.0 3.5 3.0 2.5 2.0 1.5 1.0 0.5 0
f1 (ppm)

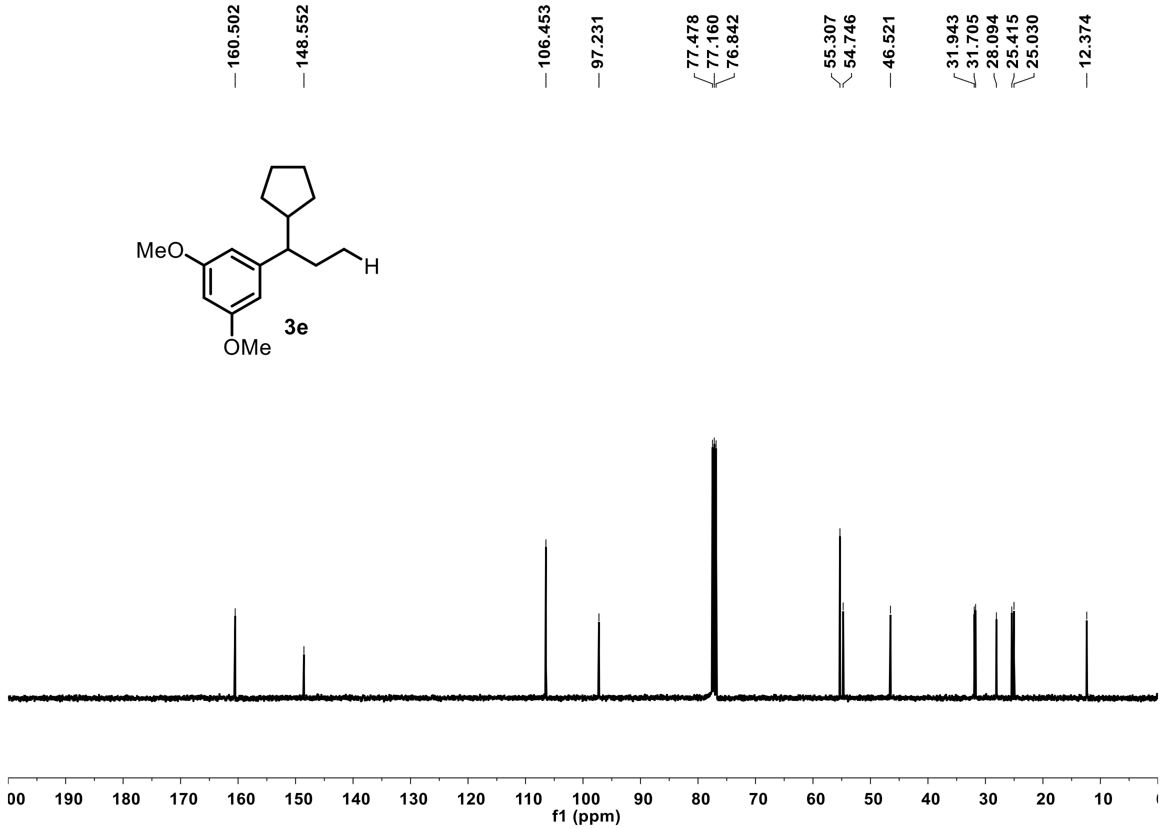
160.502
148.552
106.453
97.231
77.478
77.160
76.842
55.307
54.746
46.521
31.943
31.705
28.094
25.415
25.030
12.374
MeO
OMe
H
3e
f1 (ppm)

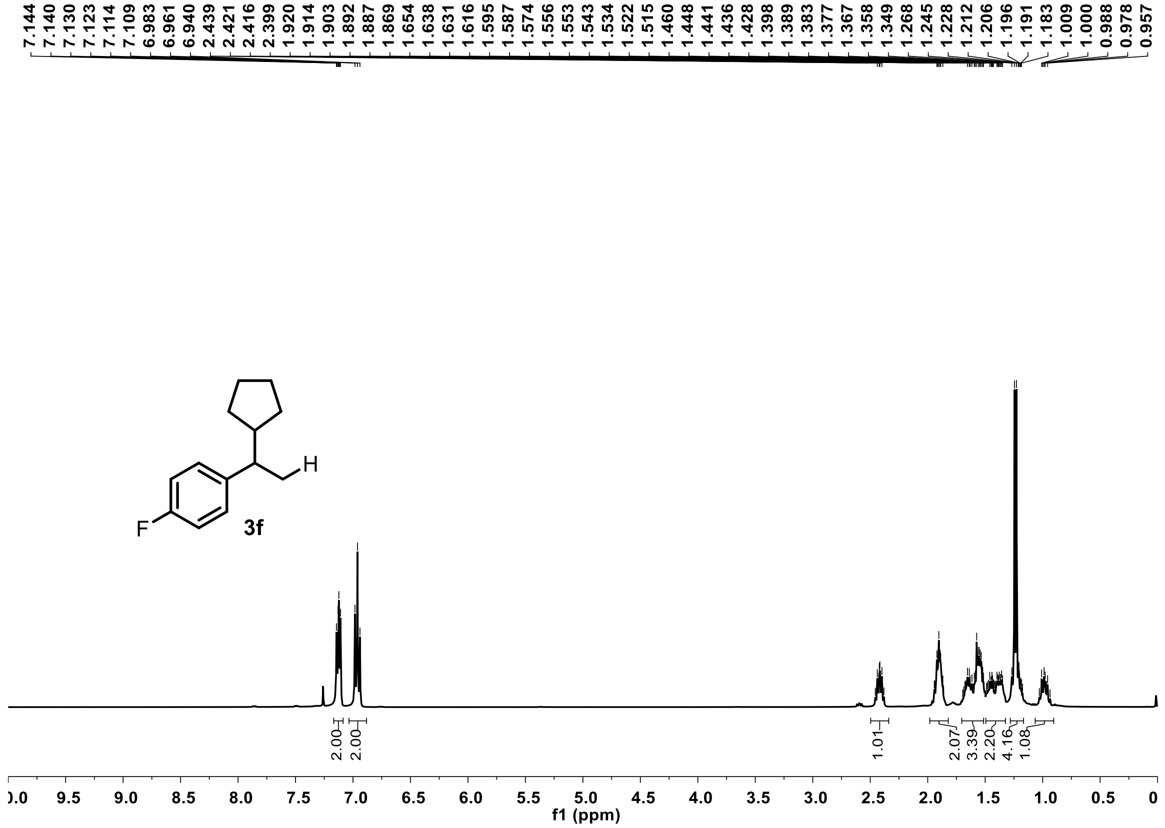
7.144
7.140
7.130
7.123
7.114
7.109
6.983
6.961
6.940
2.439
2.421
2.416
2.399
1.920
1.914
1.903
1.892
1.887
1.869
1.654
1.638
1.631
1.616
1.595
1.587
1.574
1.556
1.553
1.543
1.534
1.522
1.515
1.460
1.448
1.441
1.436
1.428
1.398
1.389
1.383
1.377
1.367
1.358
1.349
1.268
1.245
1.228
1.212
1.206
1.196
1.191
1.183
1.009
1.000
0.988
0.978
0.957
F
H
3f
2.00
2.00
1.01
2.07
3.39
2.20
4.16
1.08
f1 (ppm)

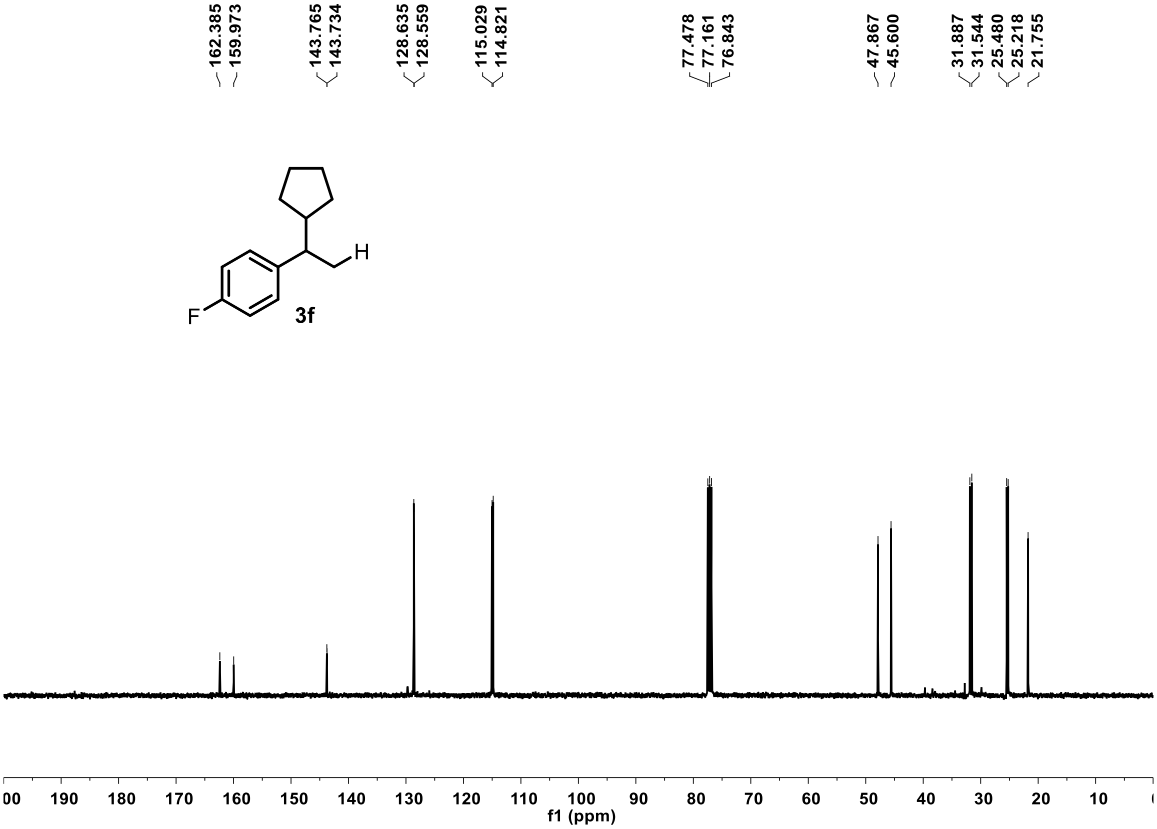
162.385
159.973
143.765
143.734
128.635
128.559
115.029
114.821
77.478
77.161
76.843
47.867
45.600
31.887
31.544
25.480
25.218
21.755
H
F
3f
f1 (ppm)

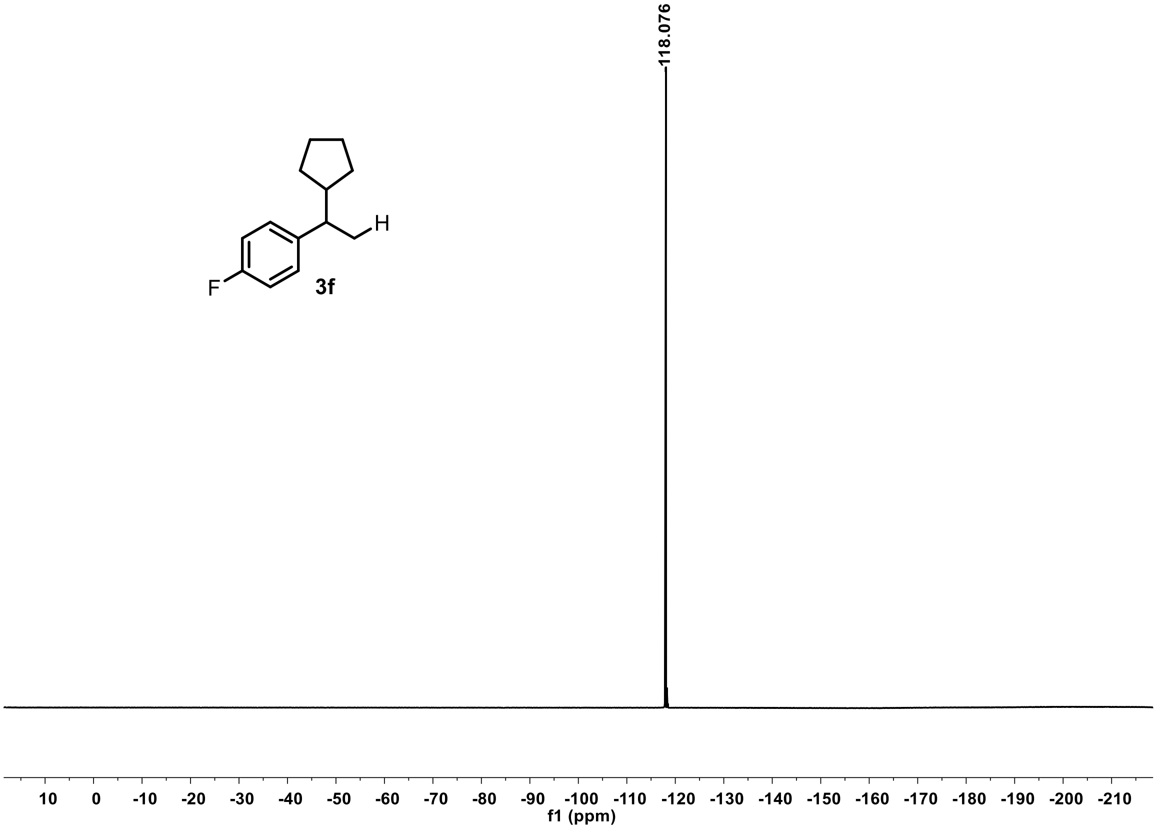
-118.076
H
F
3f
f1 (ppm)

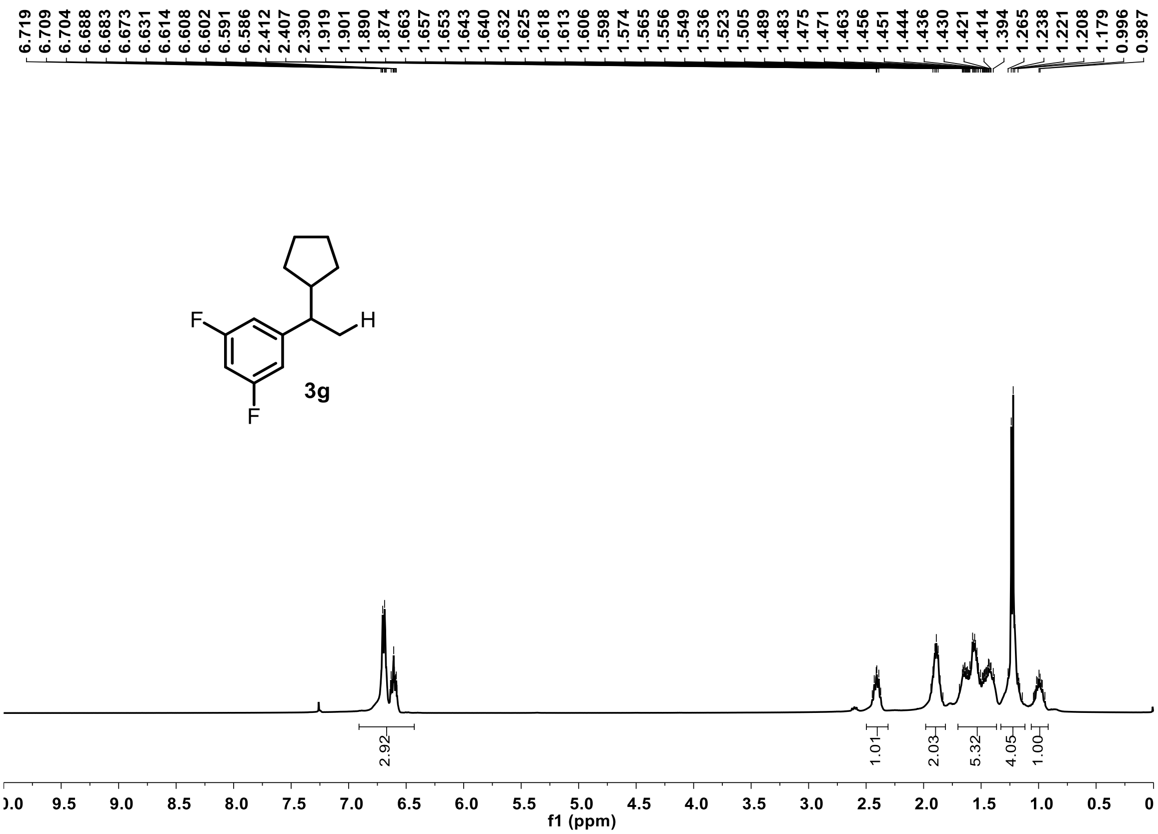
6.719 6.709 6.704 6.688 6.683 6.673 6.631 6.614 6.608 6.602 6.591 6.586 2.412 2.407 2.390 1.919 1.901 1.890 1.874 1.663 1.657 1.653 1.643 1.640 1.632 1.625 1.618 1.613 1.606 1.598 1.574 1.565 1.556 1.549 1.536 1.523 1.505 1.489 1.483 1.475 1.471 1.463 1.456 1.451 1.444 1.436 1.430 1.421 1.414 1.394 1.265 1.238 1.221 1.208 1.179 0.996 0.987
F
F
H
3g
2.92
1.01
2.03
5.32
4.05
1.00
f1 (ppm)

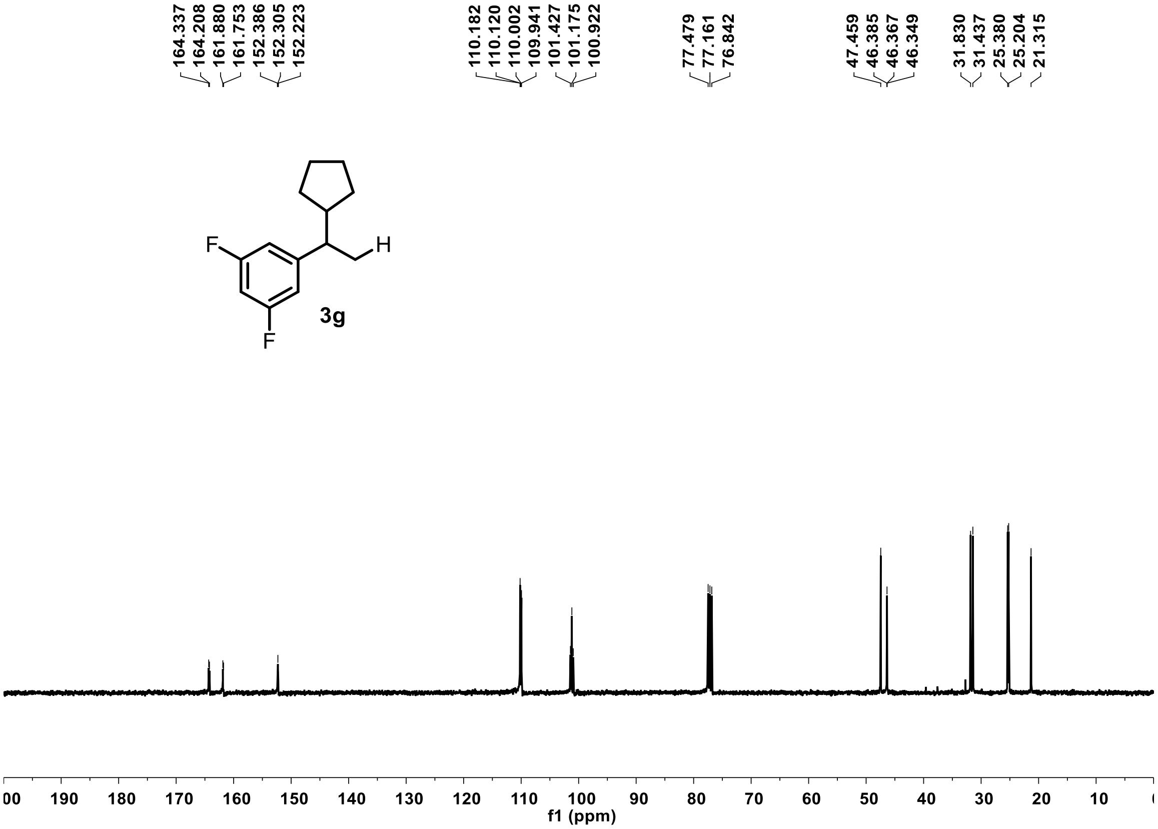
164.337 164.208 161.880 161.753 152.386 152.305 152.223
110.182 110.120 110.002 109.941 101.427 101.175 100.922
77.479 77.161 76.842
47.459 46.385 46.367 46.349
31.830 31.437 25.380 25.204 21.315
F
F
H
3g
f1 (ppm)

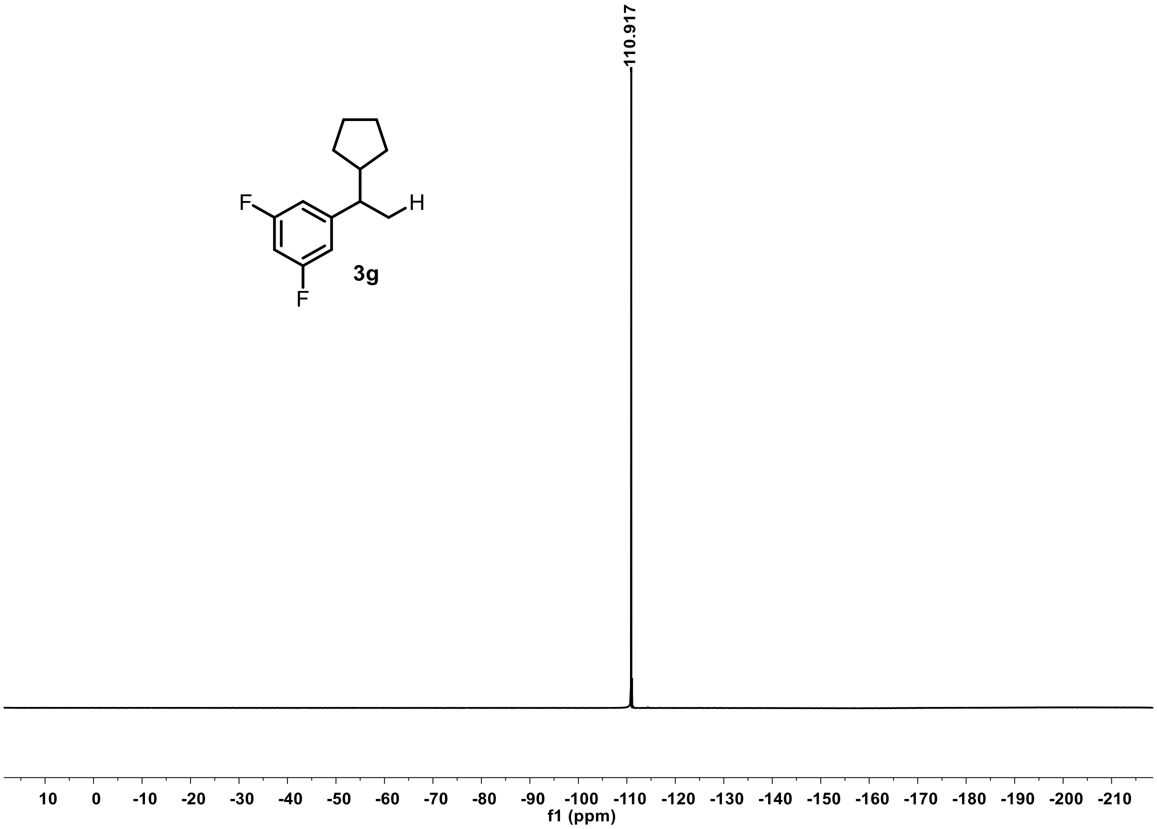
110.917
F
F
H
3g
10 0 -10 -20 -30 -40 -50 -60 -70 -80 -90 -100 -110 -120 -130 -140 -150 -160 -170 -180 -190 -200 -210
f1 (ppm)

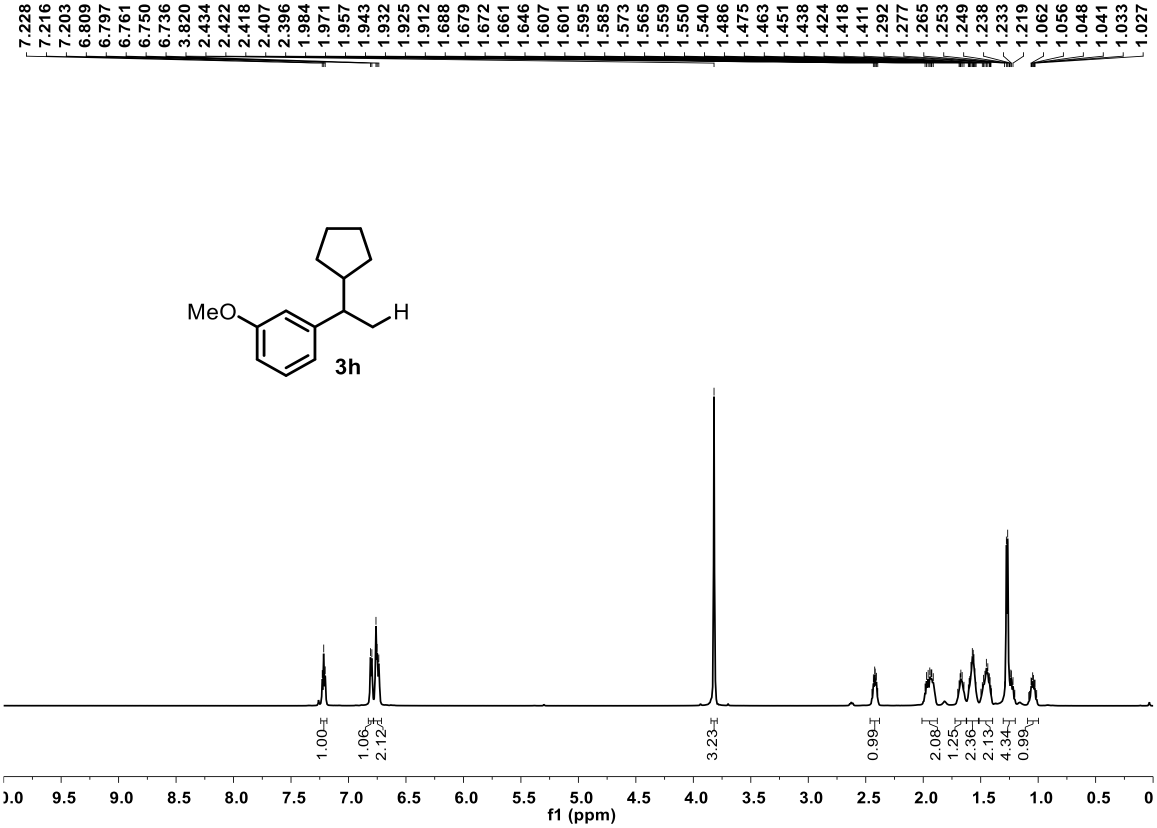
7.228 7.216 7.203 6.809 6.797 6.761 6.750 6.736 3.820 2.434 2.422 2.418 2.407 2.396 1.984 1.971 1.957 1.943 1.932 1.925 1.912 1.688 1.679 1.672 1.661 1.646 1.607 1.601 1.595 1.585 1.573 1.565 1.559 1.550 1.540 1.486 1.475 1.463 1.451 1.438 1.424 1.418 1.411 1.292 1.277 1.265 1.253 1.249 1.238 1.233 1.219 1.062 1.056 1.048 1.041 1.033 1.027
MeO
H
3h
1.00 1.06 2.12 3.23 0.99 2.08 1.25 2.36 2.13 4.34 0.99
9.5 9.0 8.5 8.0 7.5 7.0 6.5 6.0 5.5 5.0 4.5 4.0 3.5 3.0 2.5 2.0 1.5 1.0 0.5 0
f1 (ppm)

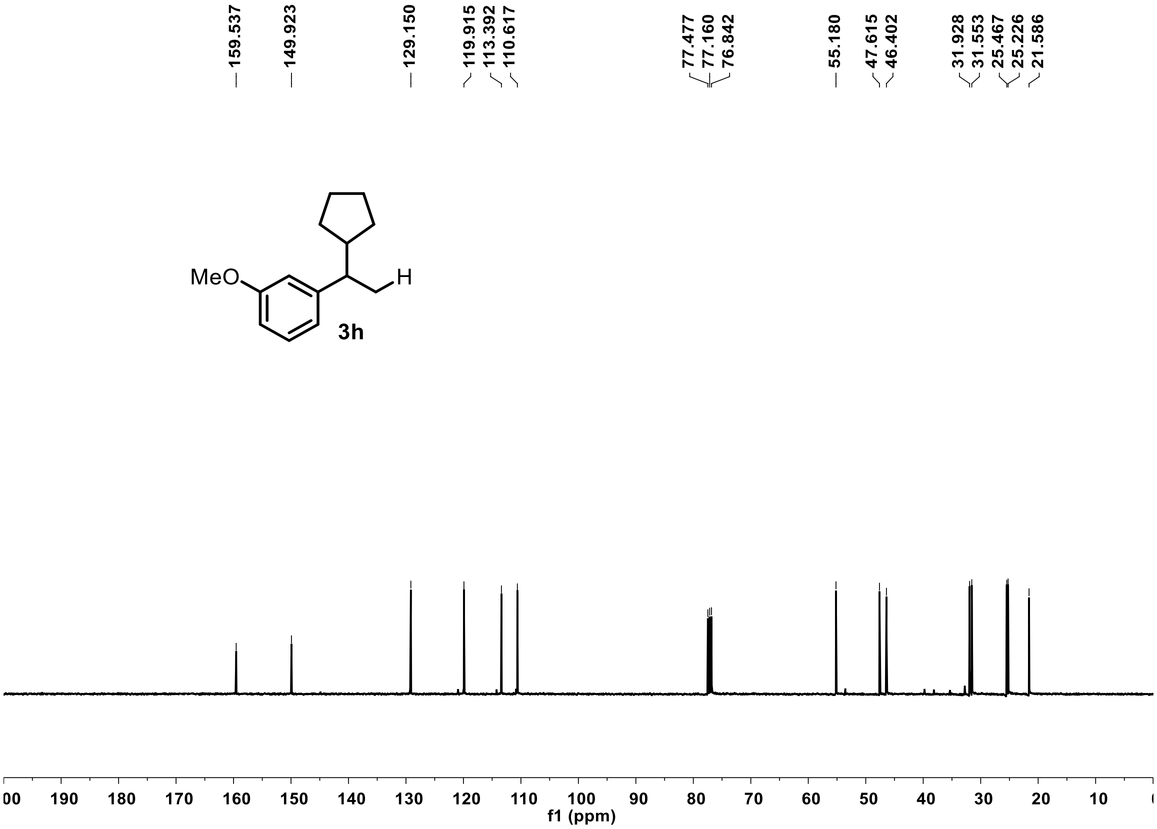
159.537
149.923
129.150
119.915
113.392
110.617
77.477
77.160
76.842
55.180
47.615
46.402
31.928
31.553
25.467
25.226
21.586
MeO
H
3h
00 190 180 170 160 150 140 130 120 110 100 90 80 70 60 50 40 30 20 10
f1 (ppm)

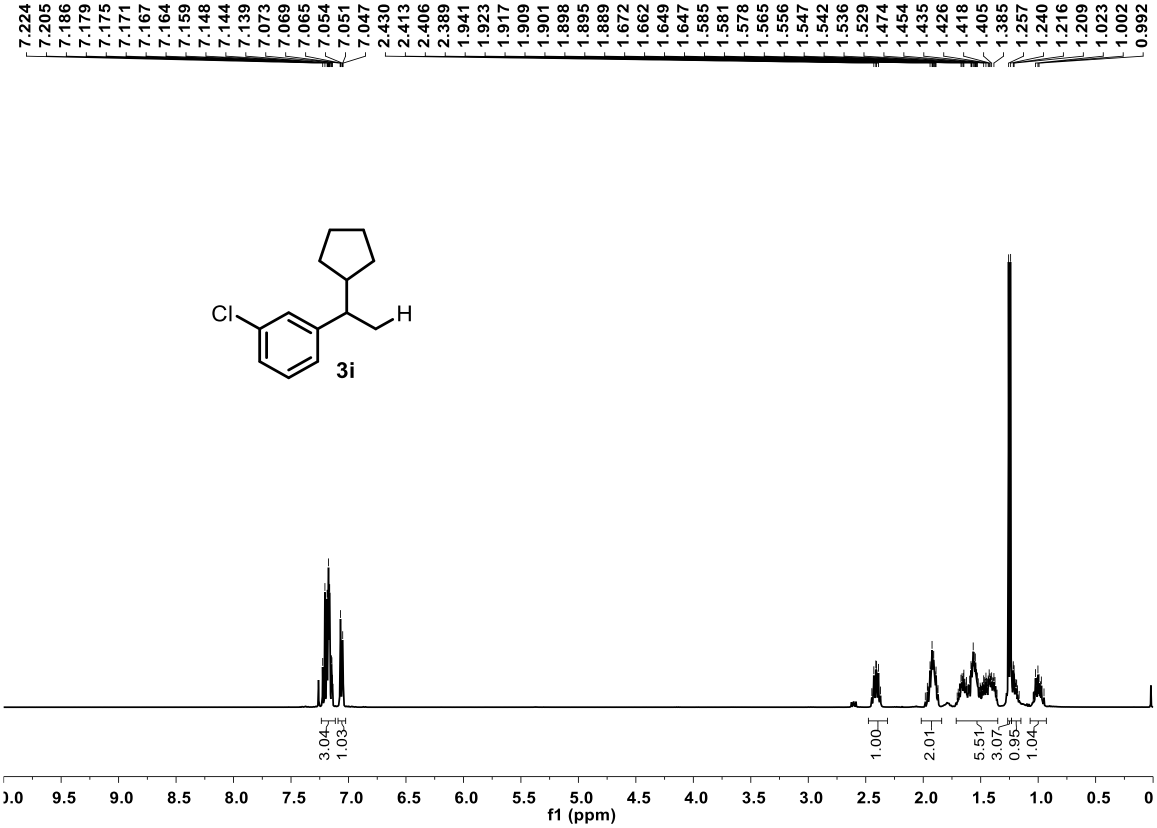
7.224
7.205
7.186
7.179
7.175
7.171
7.167
7.164
7.159
7.148
7.144
7.139
7.073
7.069
7.065
7.054
7.051
7.047
2.430
2.413
2.406
2.389
1.941
1.923
1.917
1.909
1.901
1.898
1.895
1.889
1.672
1.662
1.649
1.647
1.585
1.581
1.578
1.565
1.556
1.547
1.542
1.536
1.529
1.474
1.454
1.435
1.426
1.418
1.405
1.385
1.257
1.240
1.216
1.209
1.023
1.002
0.992
Cl
H
3i
3.04
1.03
1.00
2.01
5.51
3.07
0.95
1.04
9.5 9.0 8.5 8.0 7.5 7.0 6.5 6.0 5.5 5.0 4.5 4.0 3.5 3.0 2.5 2.0 1.5 1.0 0.5 0
f1 (ppm)

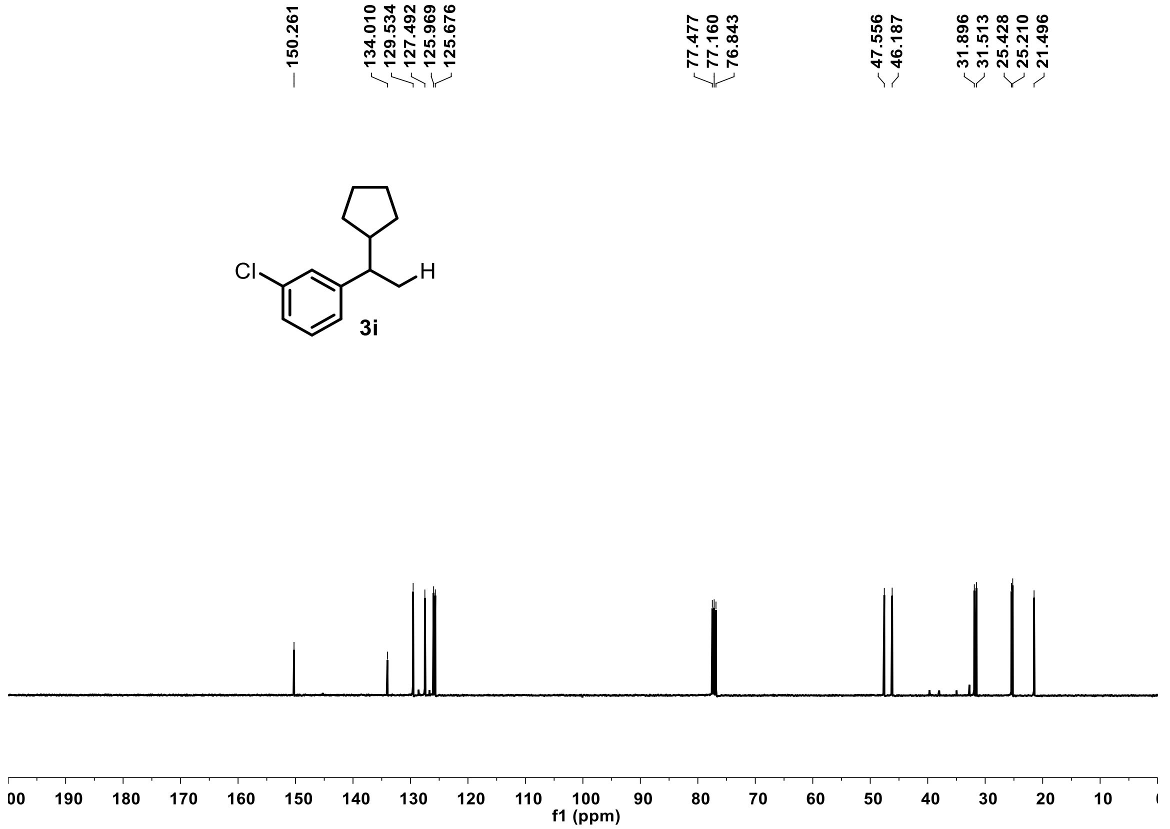
150.261
134.010
129.534
127.492
125.969
125.676
77.477
77.160
76.843
47.556
46.187
31.896
31.513
25.428
25.210
21.496
Cl
H
3i
f1 (ppm)

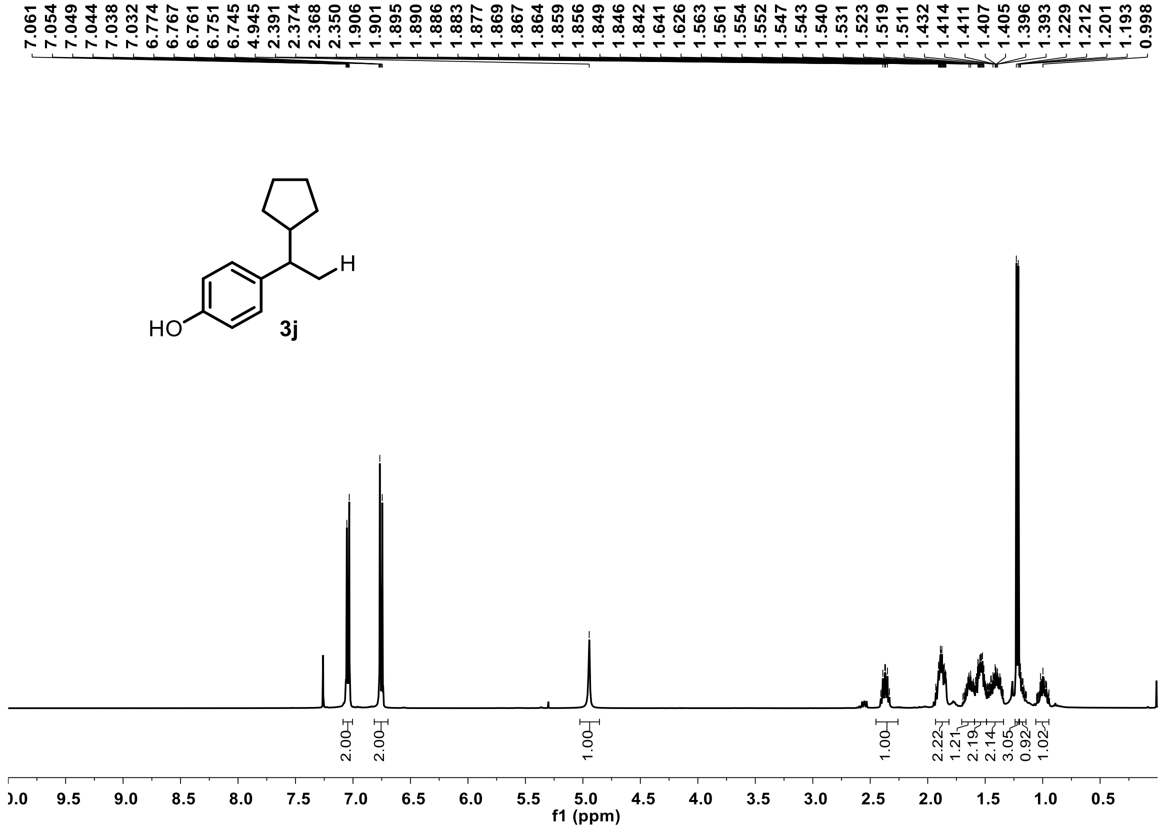
7.061
7.054
7.049
7.044
7.038
7.032
6.774
6.767
6.761
6.751
6.745
4.945
2.391
2.374
2.368
2.350
1.906
1.901
1.895
1.890
1.886
1.883
1.877
1.869
1.867
1.864
1.859
1.856
1.849
1.846
1.842
1.641
1.626
1.563
1.561
1.554
1.552
1.547
1.543
1.540
1.531
1.523
1.519
1.511
1.432
1.414
1.411
1.407
1.405
1.396
1.393
1.229
1.212
1.201
1.193
0.998
HO
H
3j
2.00
2.00
1.00
1.00
2.22
1.21
2.19
2.14
3.05
0.92
1.02
f1 (ppm)

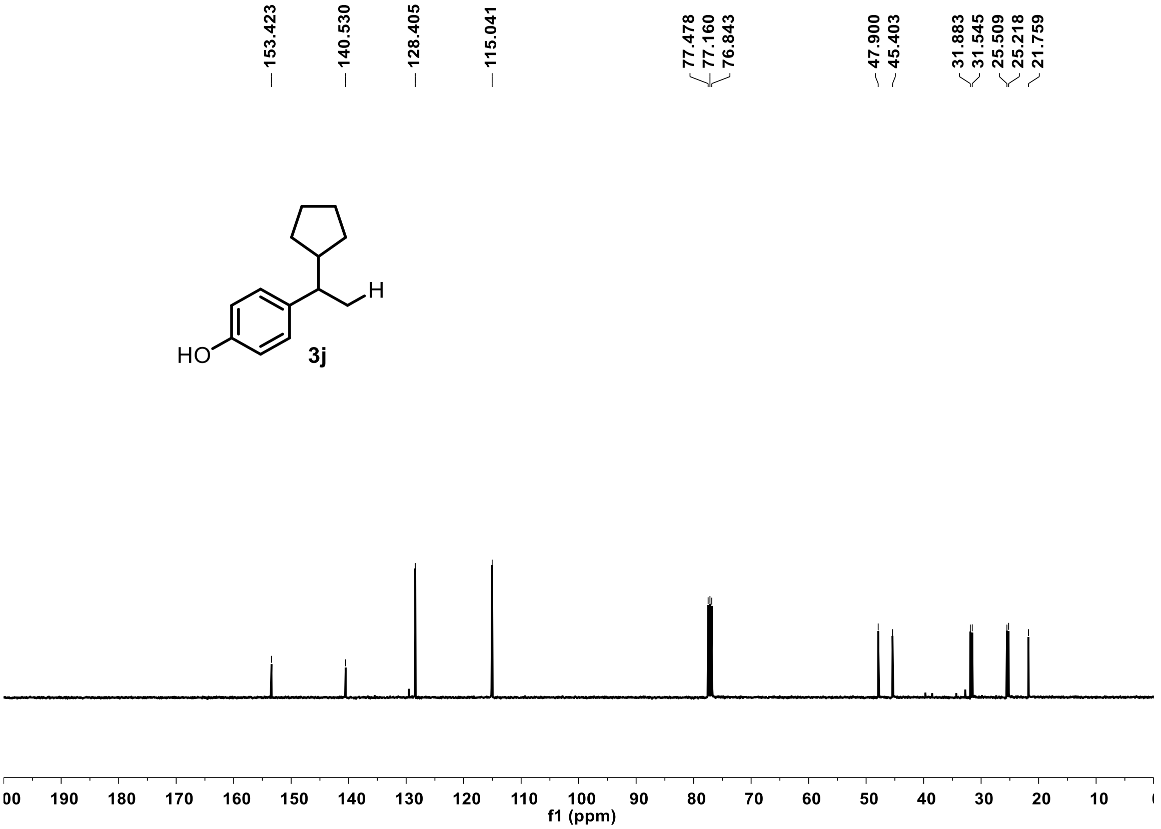
153.423
140.530
128.405
115.041
77.478
77.160
76.843
47.900
45.403
31.883
31.545
25.509
25.218
21.759
HO
H
3j
f1 (ppm)

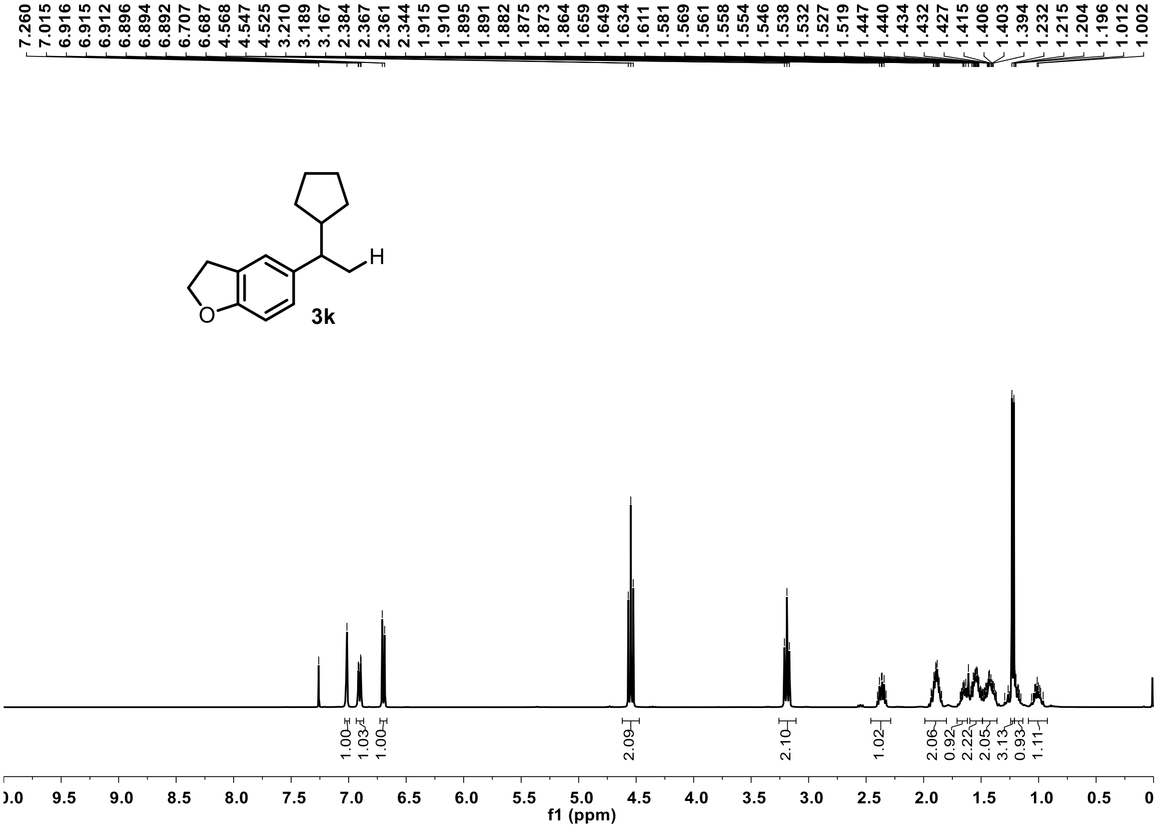
7.260
7.015
6.916
6.915
6.912
6.896
6.894
6.892
6.707
6.687
4.568
4.547
4.525
3.210
3.189
3.167
2.384
2.367
2.361
2.344
1.915
1.910
1.895
1.891
1.882
1.875
1.873
1.864
1.659
1.649
1.634
1.611
1.581
1.569
1.561
1.558
1.554
1.546
1.538
1.532
1.527
1.519
1.447
1.440
1.434
1.432
1.427
1.415
1.406
1.403
1.394
1.232
1.215
1.204
1.196
1.012
1.002
O
H
3k
1.00
1.03
1.00
2.09
2.10
1.02
2.06
0.92
2.22
2.05
3.13
0.93
1.11
f1 (ppm)

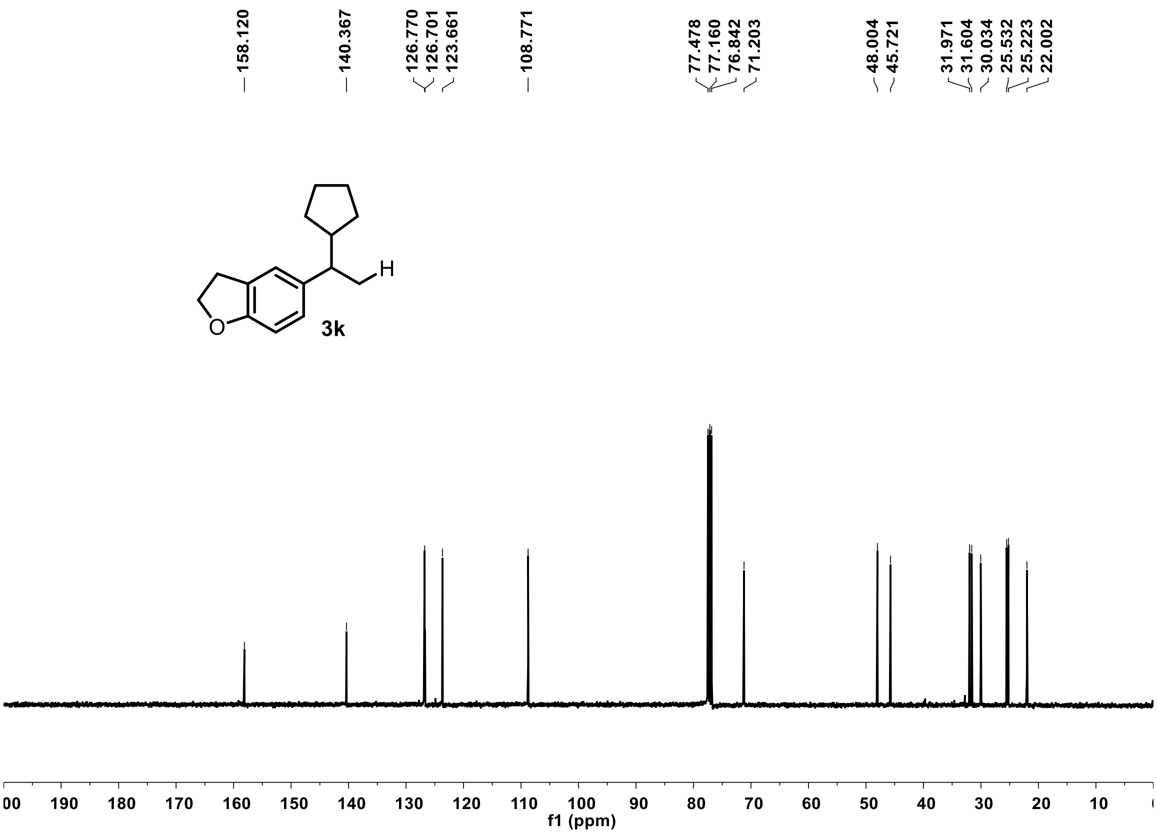
158.120
140.367
126.770
126.701
123.661
108.771
77.478
77.160
76.842
71.203
48.004
45.721
31.971
31.604
30.034
25.532
25.223
22.002
H
O
3k
f1 (ppm)

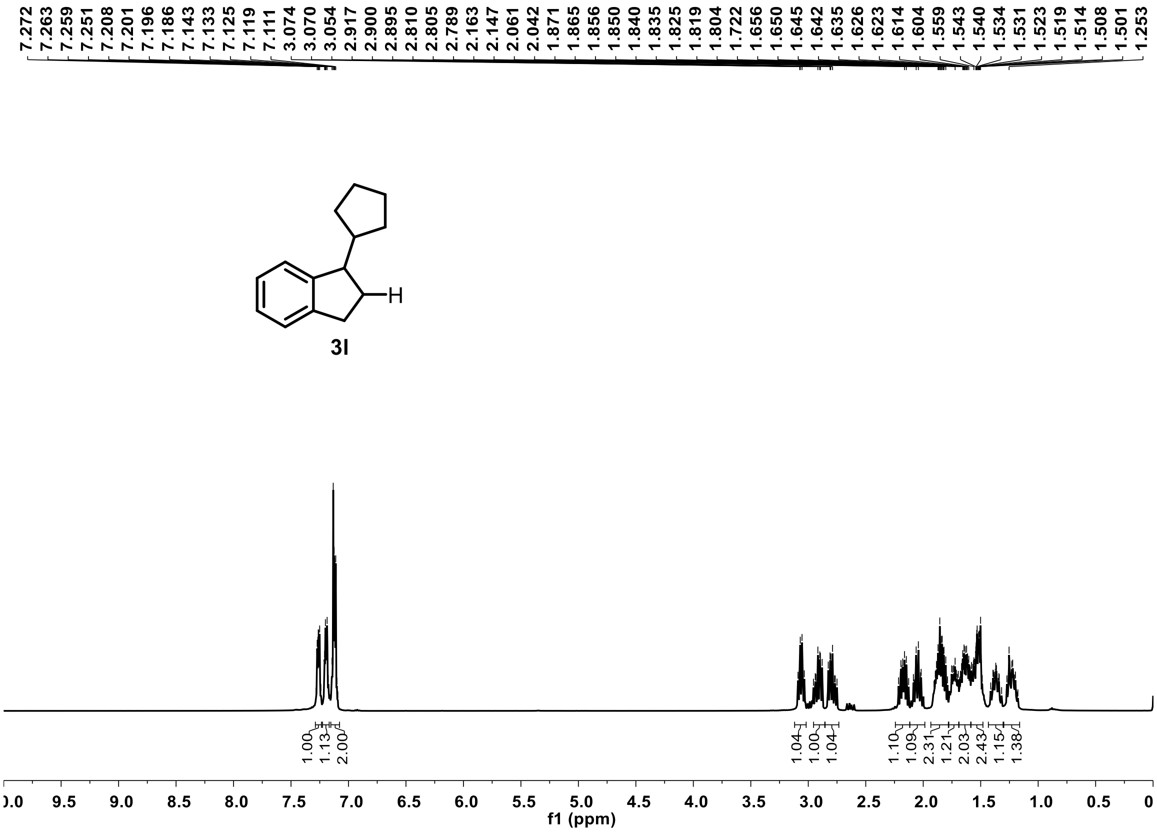
7.272
7.263
7.259
7.251
7.208
7.201
7.196
7.186
7.143
7.133
7.125
7.119
7.111
3.074
3.070
3.054
2.917
2.900
2.895
2.810
2.805
2.789
2.163
2.147
2.061
2.042
1.871
1.865
1.856
1.850
1.840
1.835
1.825
1.819
1.804
1.722
1.656
1.650
1.645
1.642
1.635
1.626
1.623
1.614
1.604
1.559
1.543
1.540
1.534
1.531
1.523
1.519
1.514
1.508
1.501
1.253
H
3l
1.00
1.13
2.00
1.04
1.00
1.04
1.10
1.09
2.31
1.21
2.03
2.43
1.15
1.38
f1 (ppm)

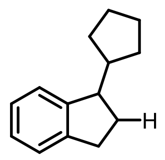

3l

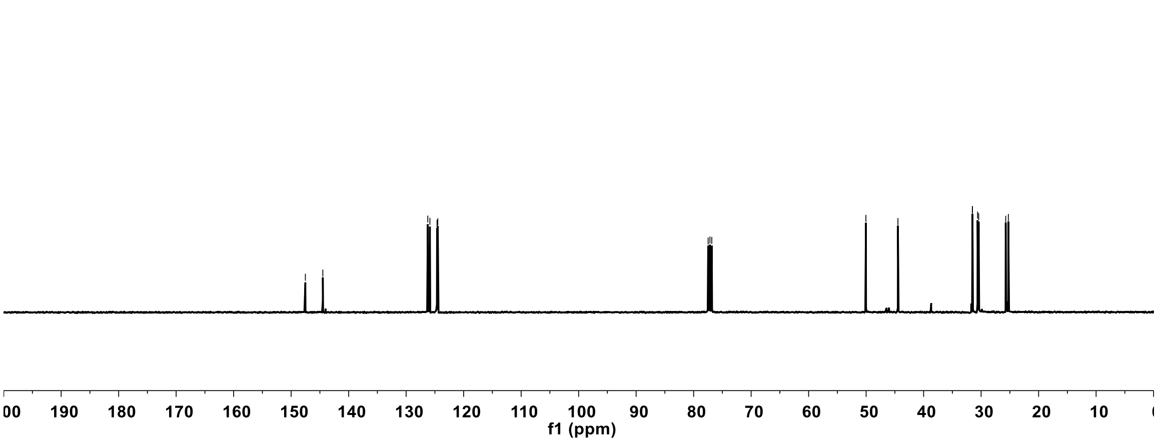

147.528
144.489
126.231
125.866
124.623
124.496
77.477
77.160
76.842
50.038
44.466
31.528
31.504
30.617
30.424
25.722
25.268
00 190 180 170 160 150 140 130 120 110 100 90 80 70 60 50 40 30 20 10
f1 (ppm)

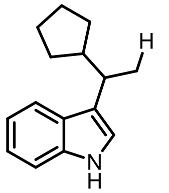

3m

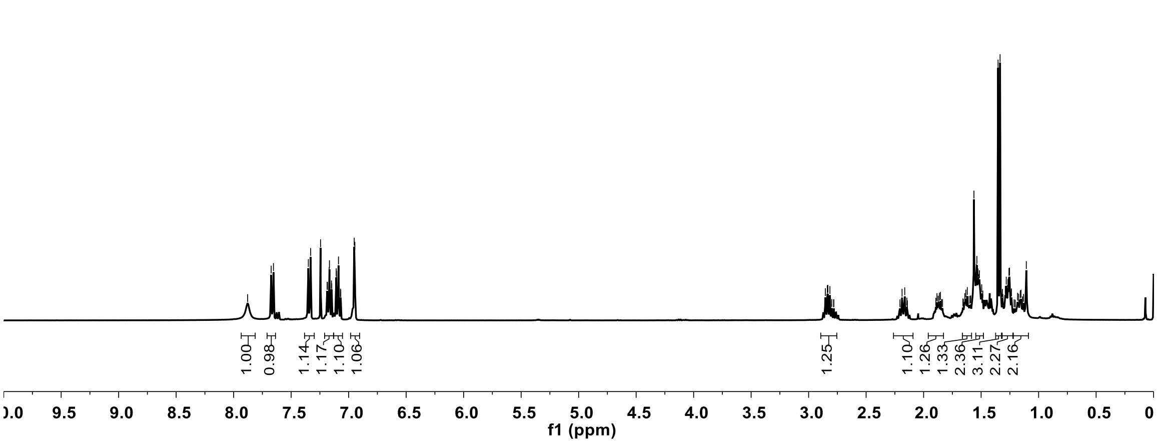

7.674
7.654
7.351
7.330
7.244
7.187
7.184
7.169
7.167
7.149
7.147
7.108
7.106
7.088
7.071
7.069
6.951
6.946
2.855
2.837
2.832
2.815
2.187
2.164
1.884
1.864
1.854
1.642
1.633
1.620
1.569
1.562
1.551
1.542
1.537
1.527
1.521
1.515
1.495
1.485
1.353
1.335
1.315
1.311
1.290
1.281
1.274
1.267
1.258
1.253
1.236
1.183
1.173
1.159
1.153
1.131
1.107
1.00
0.98
1.14
1.17
1.10
1.06
1.25
1.10
1.26
1.33
2.36
3.11
2.27
2.16
9.5 9.0 8.5 8.0 7.5 7.0 6.5 6.0 5.5 5.0 4.5 4.0 3.5 3.0 2.5 2.0 1.5 1.0 0.5 0
f1 (ppm)

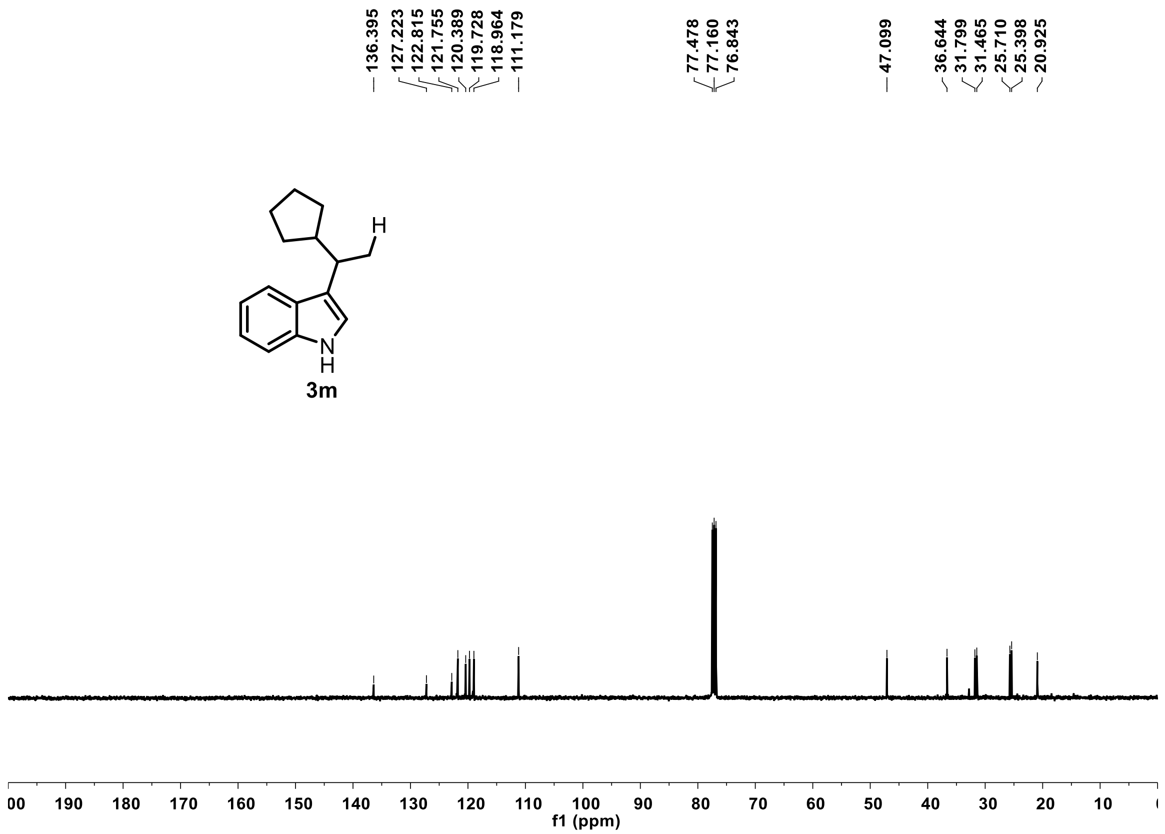

136.395
127.223
122.815
121.755
120.389
119.728
118.964
111.179
77.478
77.160
76.843
47.099
36.644
31.799
31.465
25.710
25.398
20.925
H
N
H
3m
00 190 180 170 160 150 140 130 120 110 100 90 80 70 60 50 40 30 20 10
f1 (ppm)

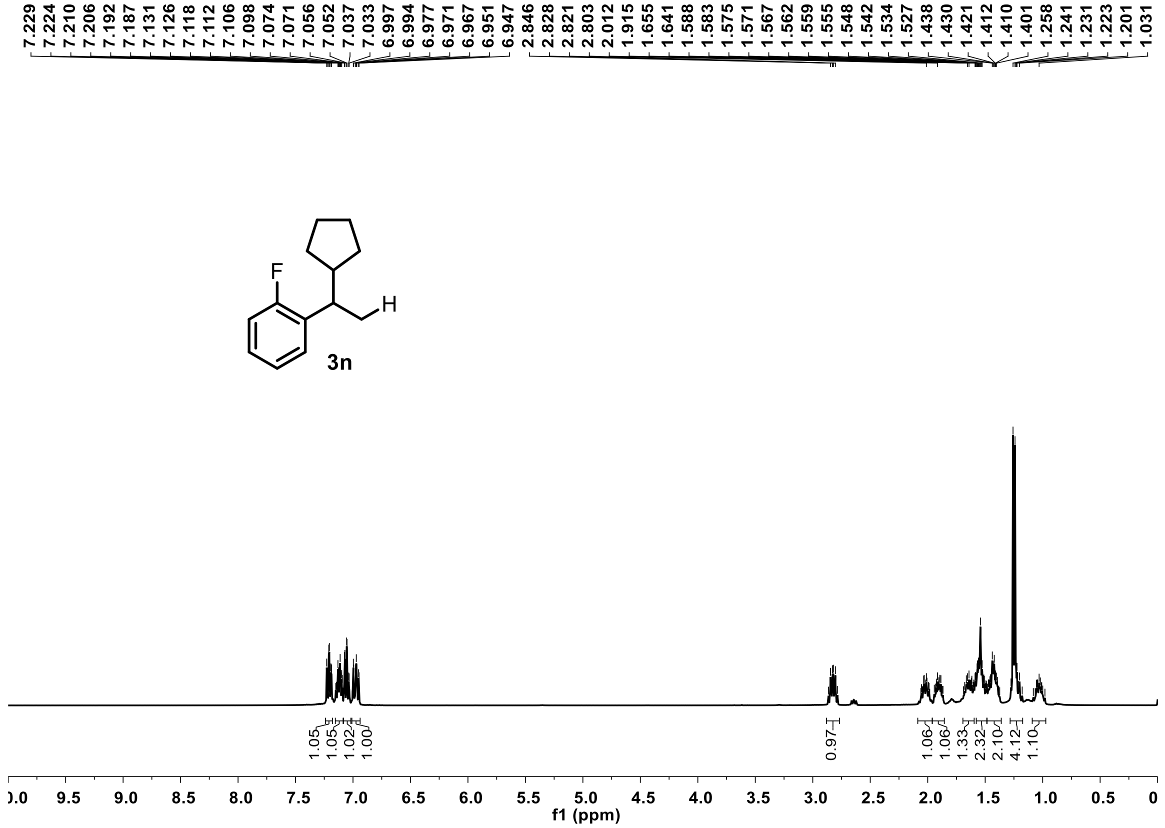

7.229
7.224
7.210
7.206
7.192
7.187
7.131
7.126
7.118
7.112
7.106
7.098
7.074
7.071
7.056
7.052
7.037
7.033
6.997
6.994
6.977
6.971
6.967
6.951
6.947
2.846
2.828
2.821
2.803
2.012
1.915
1.655
1.641
1.588
1.583
1.575
1.571
1.567
1.562
1.559
1.555
1.548
1.542
1.534
1.527
1.438
1.430
1.421
1.412
1.410
1.401
1.258
1.241
1.231
1.223
1.201
1.031
F
H
3n
1.05
1.05
1.02
1.00
0.97
1.06
1.06
1.33
2.32
2.10
4.12
1.10
9.5 9.0 8.5 8.0 7.5 7.0 6.5 6.0 5.5 5.0 4.5 4.0 3.5 3.0 2.5 2.0 1.5 1.0 0.5 0
f1 (ppm)

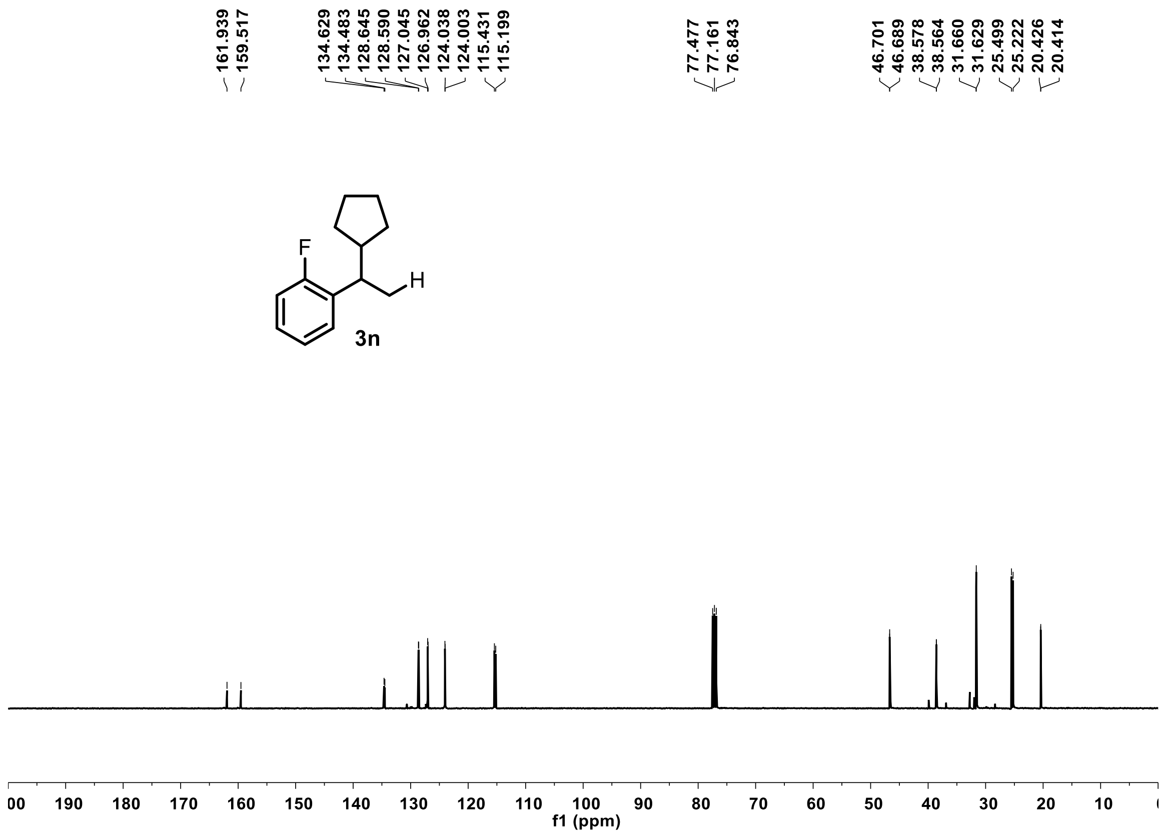
161.939
159.517
134.629
134.483
128.645
128.590
127.045
126.962
124.038
124.003
115.431
115.199
77.477
77.161
76.843
46.701
46.689
38.578
38.564
31.660
31.629
25.499
25.222
20.426
20.414
F
H
3n
190 180 170 160 150 140 130 120 110 100 90 80 70 60 50 40 30 20 10
f1 (ppm)

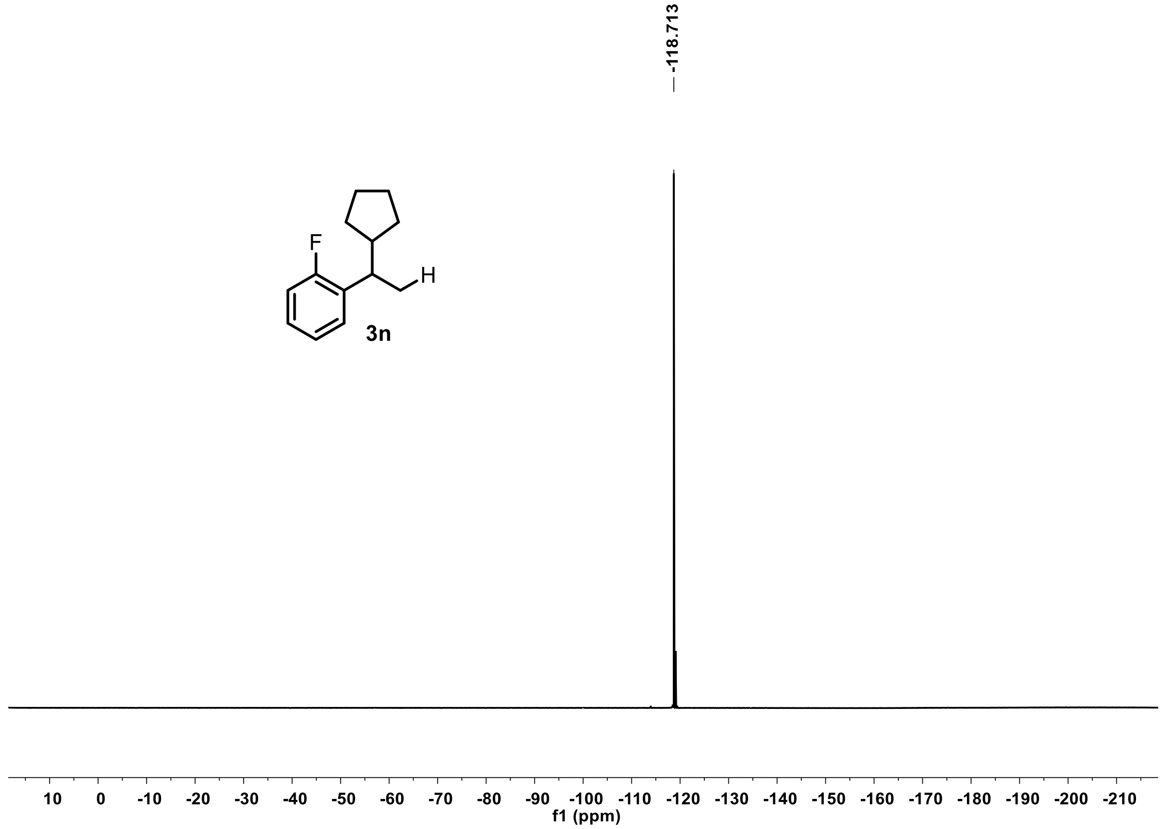
-118.713
F
H
3n
10 0 -10 -20 -30 -40 -50 -60 -70 -80 -90 -100 -110 -120 -130 -140 -150 -160 -170 -180 -190 -200 -210
f1 (ppm)

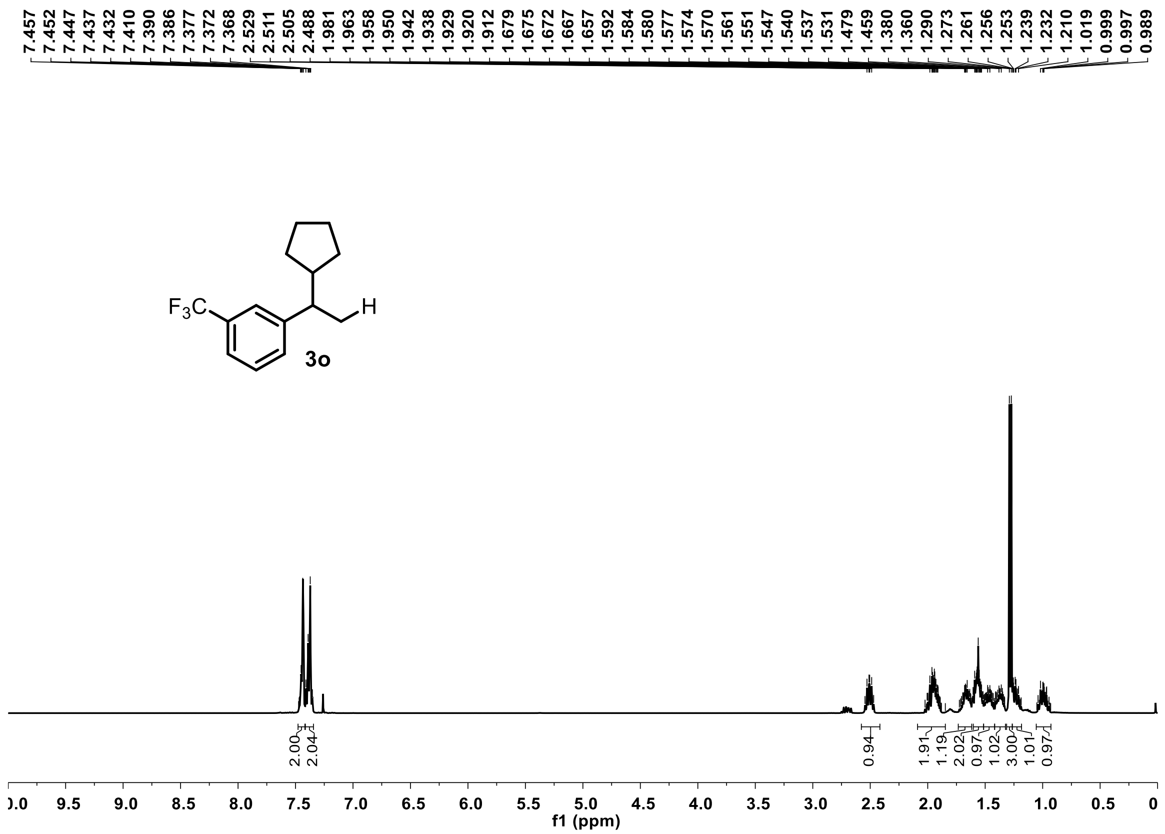
7.457 7.452 7.447 7.437 7.432 7.410 7.390 7.386 7.377 7.372 7.368 2.529 2.511 2.505 2.488 1.981 1.963 1.958 1.950 1.942 1.938 1.929 1.920 1.912 1.679 1.675 1.672 1.667 1.657 1.592 1.584 1.580 1.577 1.574 1.570 1.561 1.551 1.547 1.540 1.537 1.531 1.479 1.459 1.380 1.360 1.290 1.273 1.261 1.256 1.253 1.239 1.232 1.210 1.019 0.999 0.997 0.989
F_3C
H
3o
2.00
2.04
0.94
1.91
1.19
2.02
0.97
1.02
3.00
1.01
0.97
9.5 9.0 8.5 8.0 7.5 7.0 6.5 6.0 5.5 5.0 4.5 4.0 3.5 3.0 2.5 2.0 1.5 1.0 0.5 0
f1 (ppm)

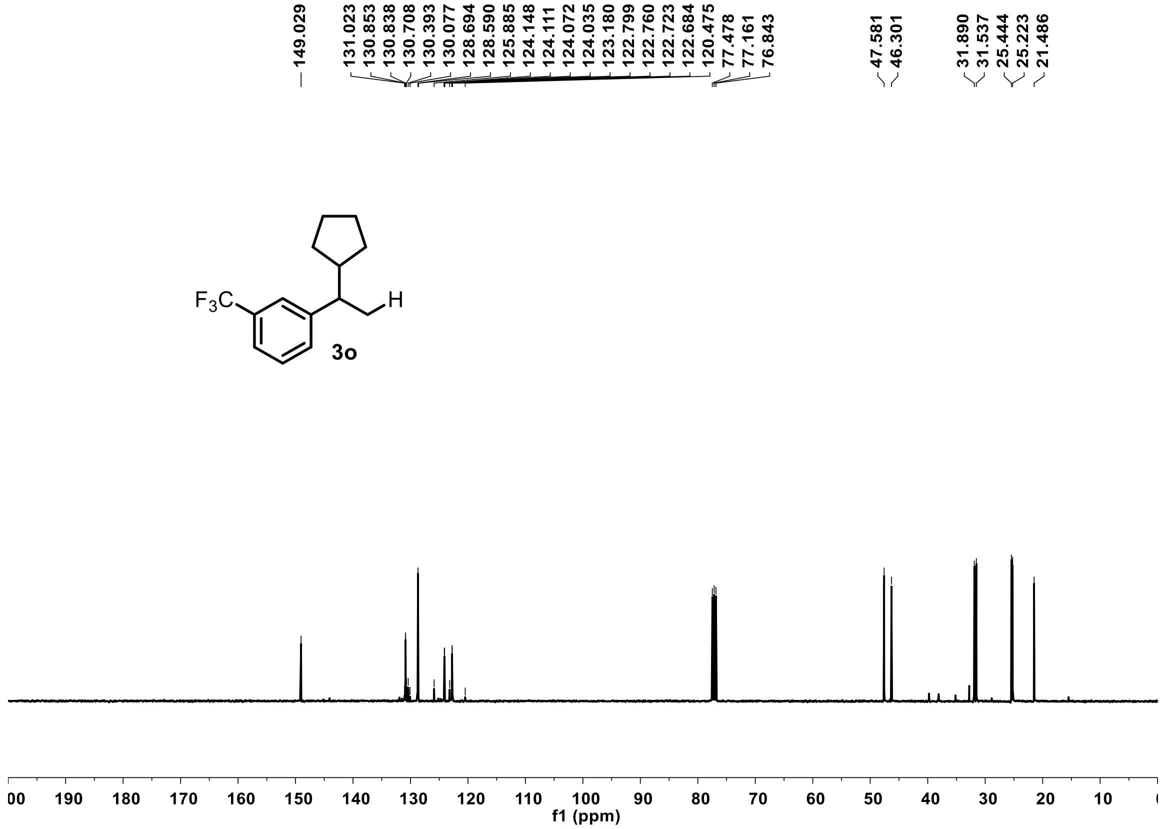
149.029
131.023 130.853 130.838 130.708 130.393 130.077 128.694 128.590 125.885 124.148 124.111 124.072 124.035 123.180 122.799 122.760 122.723 122.684 120.475 77.478 77.161 76.843
47.581 46.301
31.890 31.537 25.444 25.223 21.486
F_3C
H
3o
190 180 170 160 150 140 130 120 110 100 90 80 70 60 50 40 30 20 10
f1 (ppm)

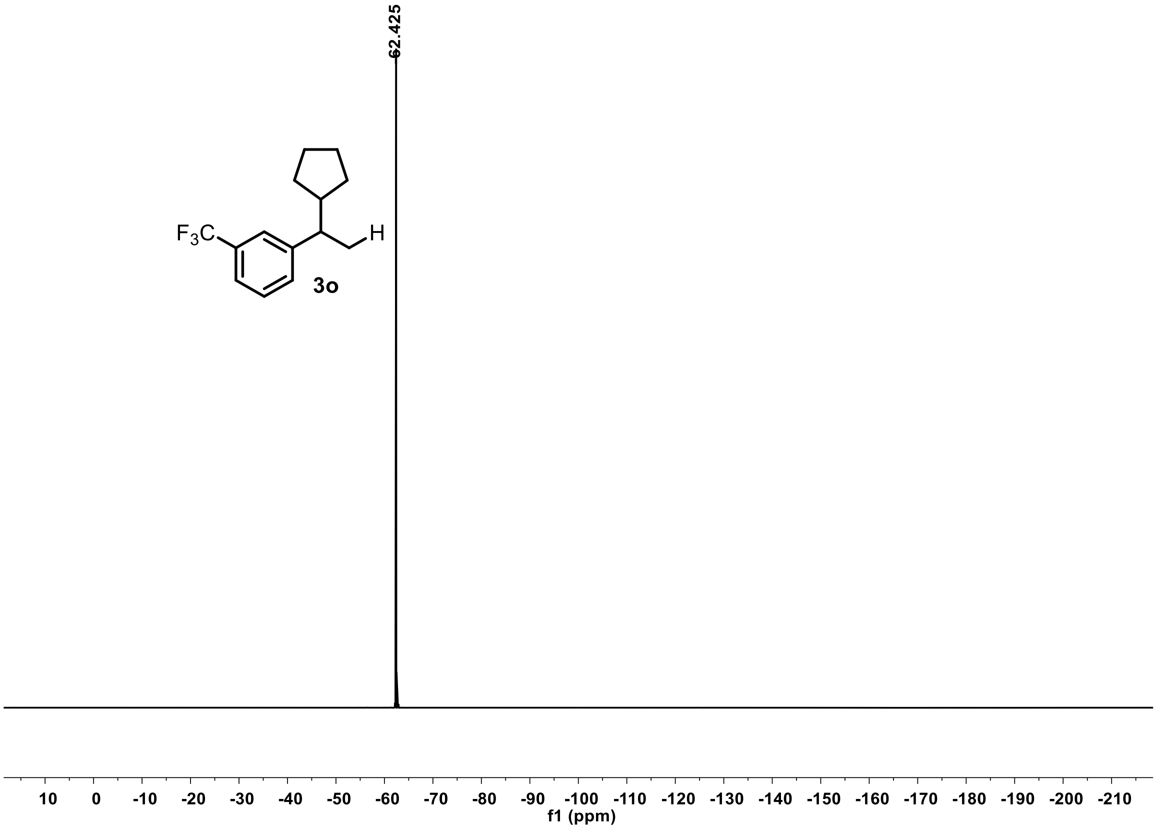
62.425
F3C
H
3o
10 0 -10 -20 -30 -40 -50 -60 -70 -80 -90 -100 -110 -120 -130 -140 -150 -160 -170 -180 -190 -200 -210
f1 (ppm)

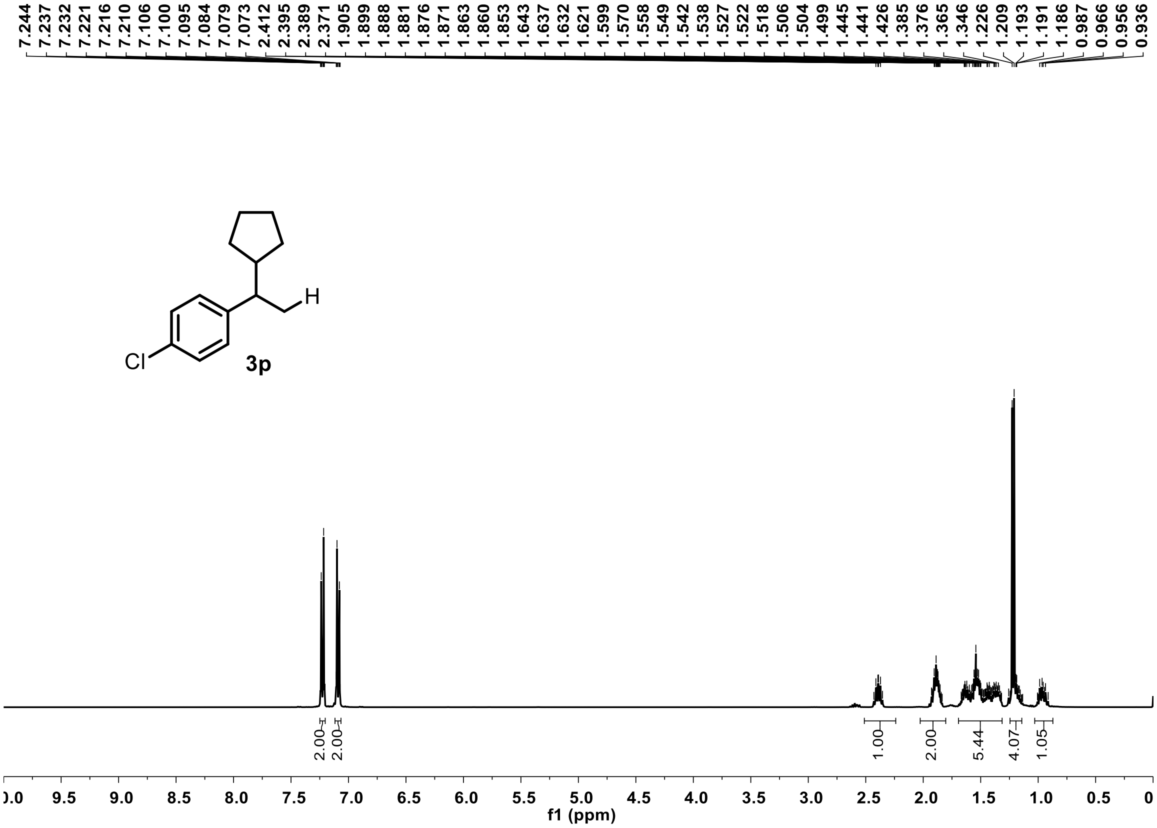
7.244 7.237 7.232 7.221 7.216 7.210 7.106 7.100 7.095 7.084 7.079 7.073 2.412 2.395 2.389 2.371 1.905 1.899 1.888 1.881 1.876 1.871 1.863 1.860 1.853 1.643 1.637 1.632 1.621 1.599 1.570 1.558 1.549 1.542 1.538 1.527 1.522 1.518 1.506 1.504 1.499 1.445 1.441 1.426 1.385 1.376 1.365 1.346 1.226 1.209 1.193 1.191 1.186 0.987 0.966 0.956 0.936
Cl
H
3p
2.00
2.00
1.00
2.00
5.44
4.07
1.05
9.5 9.0 8.5 8.0 7.5 7.0 6.5 6.0 5.5 5.0 4.5 4.0 3.5 3.0 2.5 2.0 1.5 1.0 0.5 0
f1 (ppm)

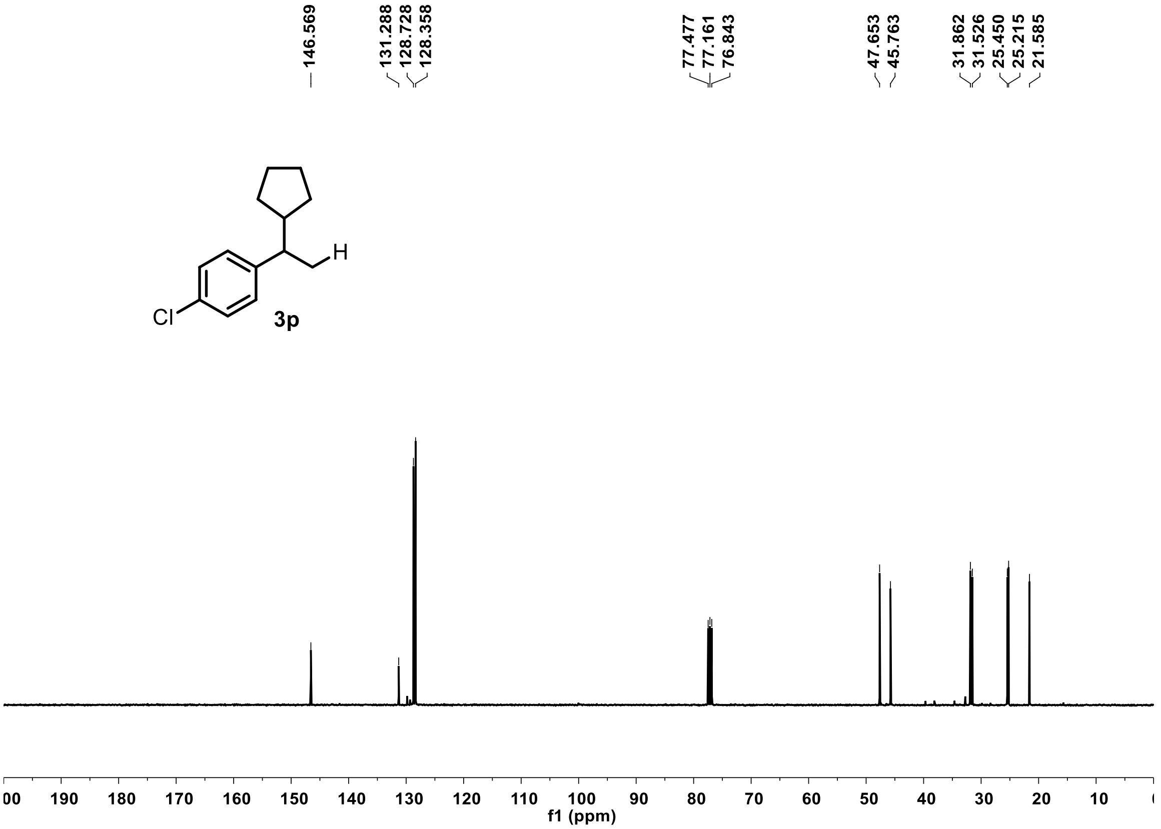
146.569
131.288
128.728
128.358
77.477
77.161
76.843
47.653
45.763
31.862
31.526
25.450
25.215
21.585
Cl
H
3p
f1 (ppm)

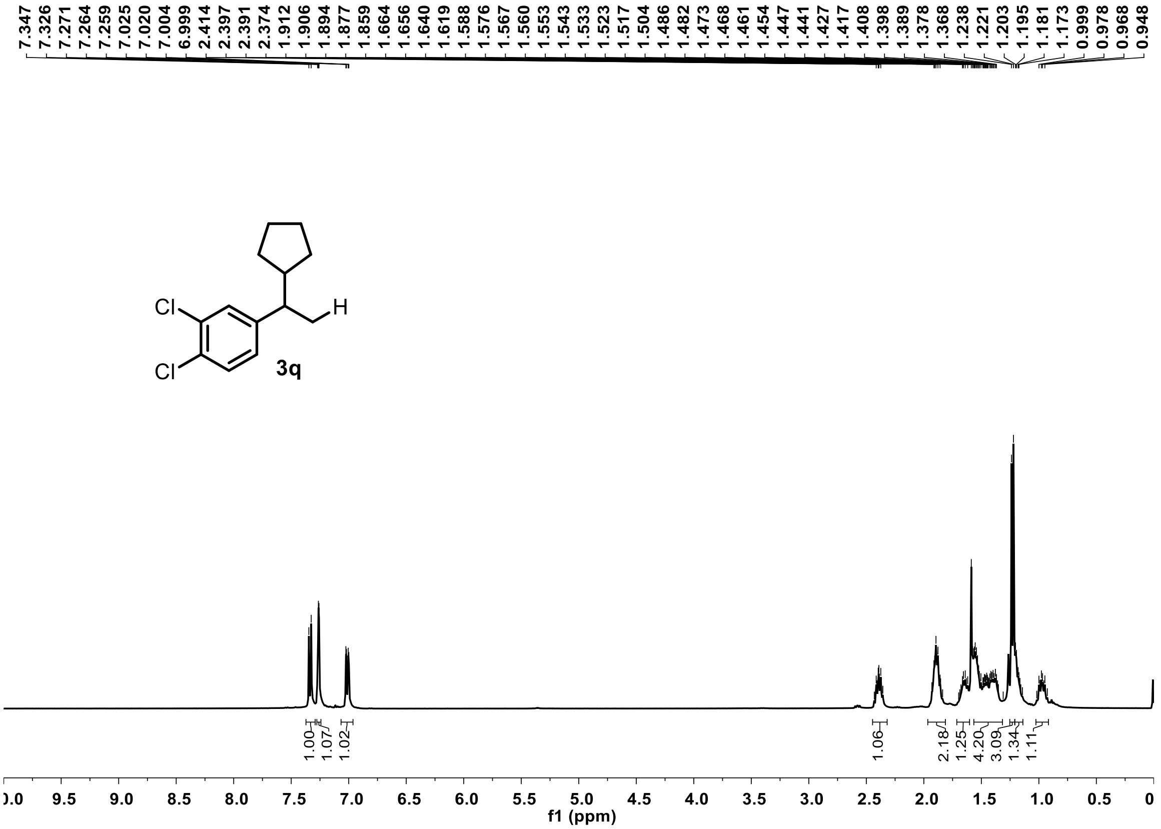
7.347
7.326
7.271
7.264
7.259
7.025
7.020
7.004
6.999
2.414
2.397
2.391
2.374
1.912
1.906
1.894
1.877
1.859
1.664
1.656
1.640
1.619
1.588
1.576
1.567
1.560
1.553
1.543
1.533
1.523
1.517
1.504
1.486
1.482
1.473
1.468
1.461
1.454
1.447
1.441
1.427
1.417
1.408
1.398
1.389
1.378
1.368
1.238
1.221
1.203
1.195
1.181
1.173
0.999
0.978
0.968
0.948
Cl
Cl
H
3q
1.00
1.07
1.02
1.06
2.18
1.25
4.20
3.09
1.34
1.11
f1 (ppm)

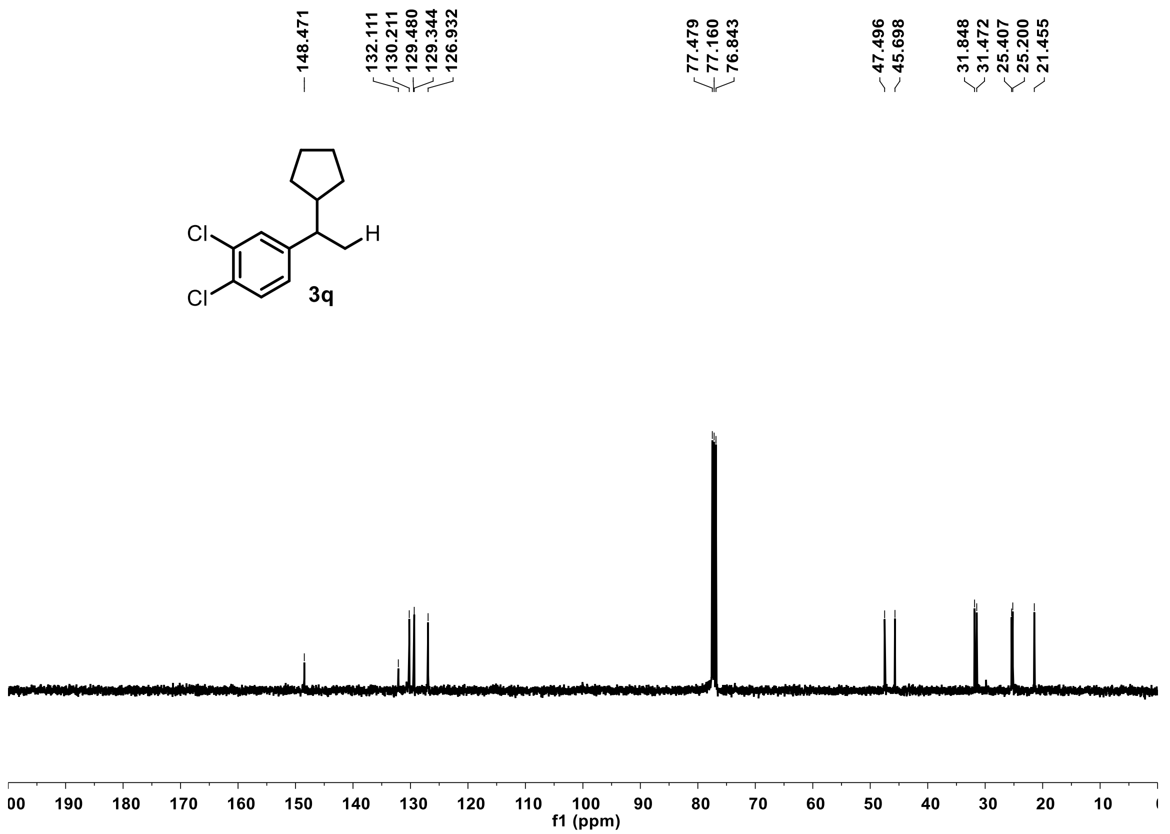
148.471
132.111
130.211
129.480
129.344
126.932
77.479
77.160
76.843
47.496
45.698
31.848
31.472
25.407
25.200
21.455
Cl
Cl
H
3q
f1 (ppm)

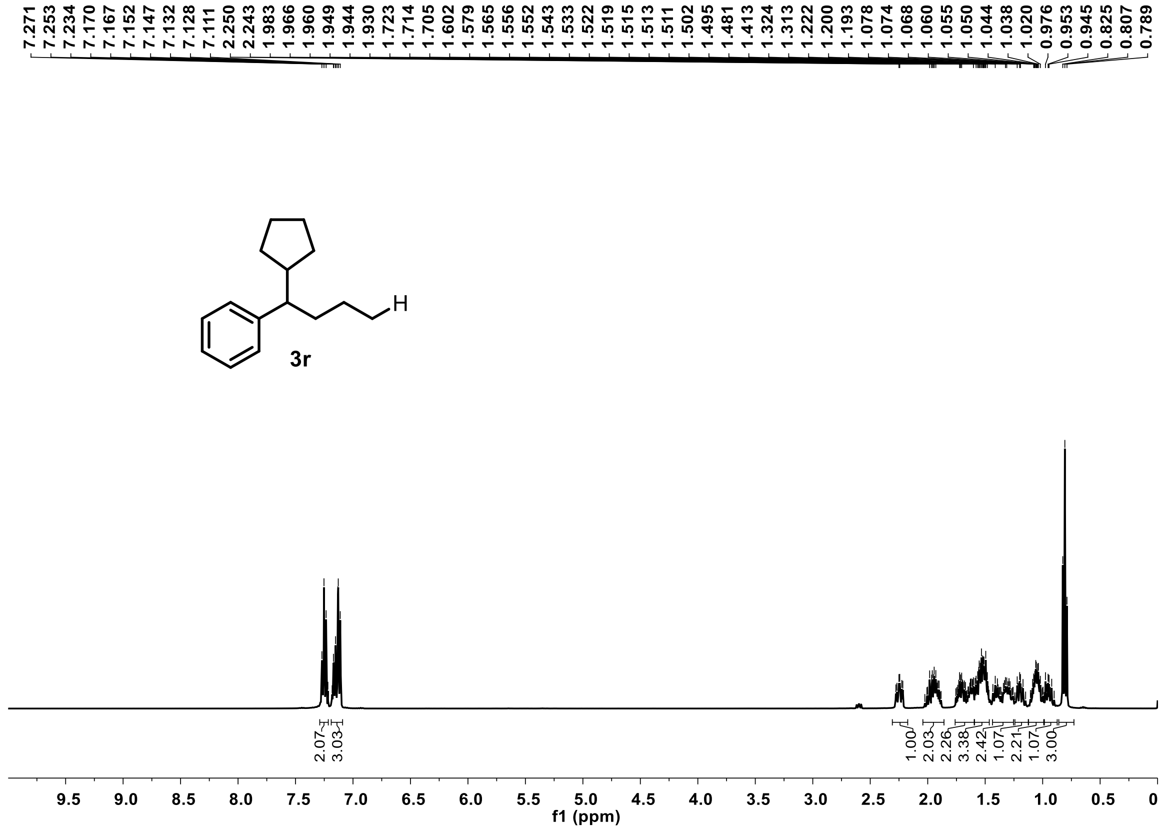
7.271
7.253
7.234
7.170
7.167
7.152
7.147
7.132
7.128
7.111
2.250
2.243
1.983
1.966
1.960
1.949
1.944
1.930
1.723
1.714
1.705
1.602
1.579
1.565
1.556
1.552
1.543
1.533
1.522
1.519
1.515
1.513
1.511
1.502
1.495
1.481
1.413
1.324
1.313
1.222
1.200
1.193
1.078
1.074
1.068
1.060
1.055
1.050
1.044
1.038
1.020
0.976
0.953
0.945
0.825
0.807
0.789
H
3r
2.07
3.03
1.00
2.03
2.26
3.38
2.42
1.07
2.21
1.07
3.00
f1 (ppm)

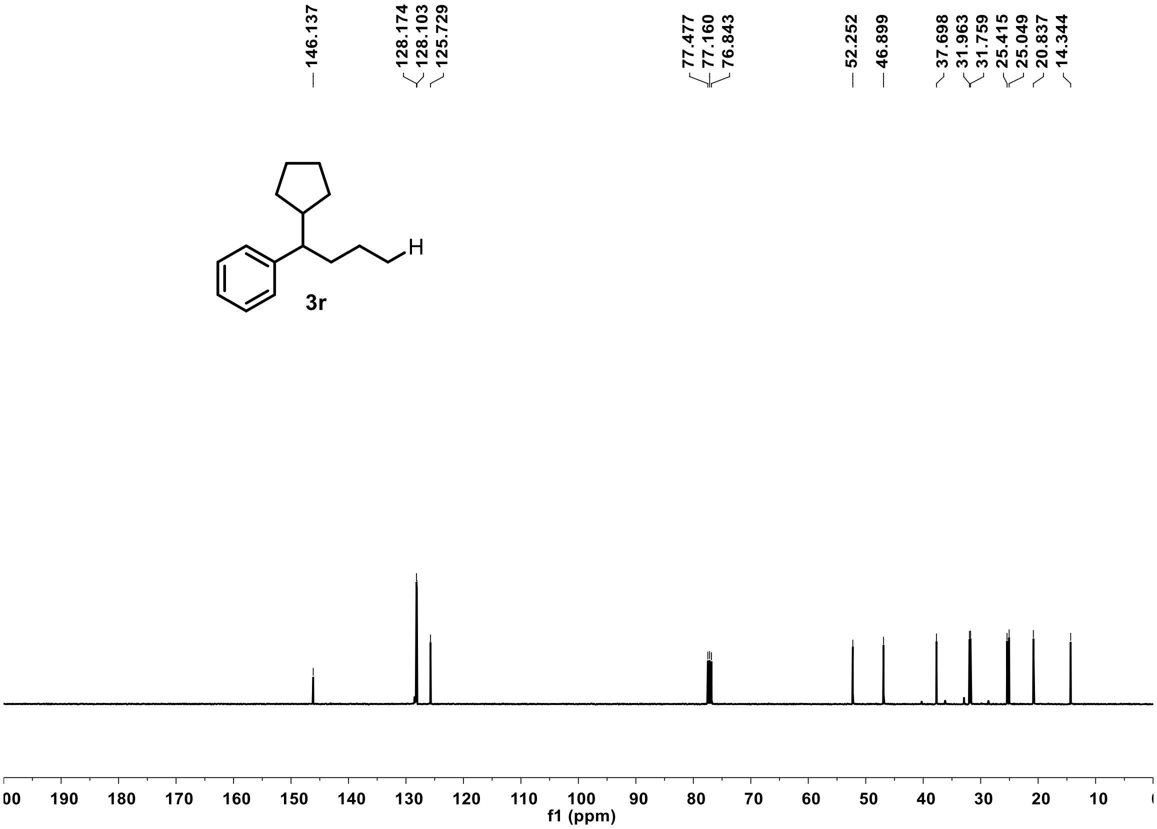
146.137
128.174
128.103
125.729
77.477
77.160
76.843
52.252
46.899
37.698
31.963
31.759
25.415
25.049
20.837
14.344
H
3r
00 190 180 170 160 150 140 130 120 110 100 90 80 70 60 50 40 30 20 10
f1 (ppm)

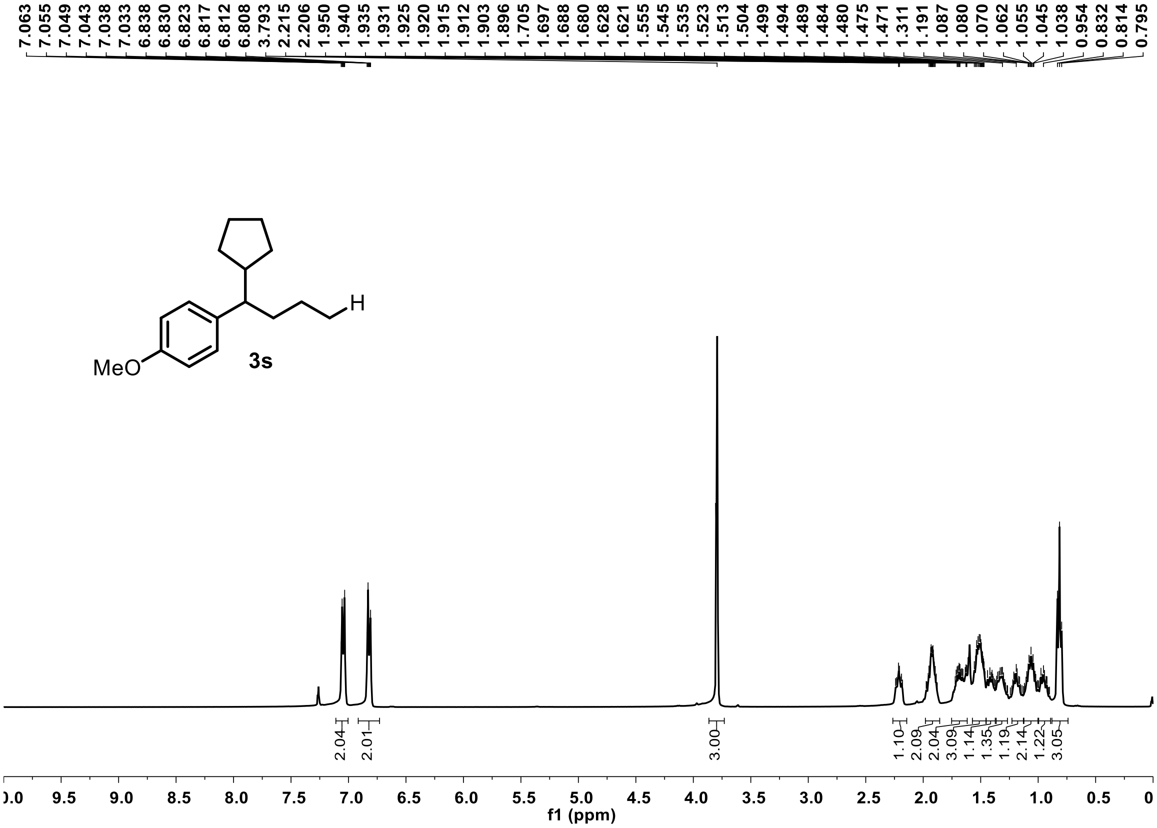
7.063
7.055
7.049
7.043
7.038
7.033
6.838
6.830
6.823
6.817
6.812
6.808
3.793
2.215
2.206
1.950
1.940
1.935
1.931
1.925
1.920
1.915
1.912
1.903
1.896
1.705
1.697
1.688
1.680
1.628
1.621
1.555
1.545
1.535
1.523
1.513
1.504
1.499
1.494
1.489
1.484
1.480
1.475
1.471
1.311
1.191
1.087
1.080
1.070
1.062
1.055
1.045
1.038
0.954
0.832
0.814
0.795
H
MeO
3s
2.04
2.01
3.00
1.10
2.09
2.04
3.09
1.14
1.35
1.19
2.14
1.22
3.05
9.5 9.0 8.5 8.0 7.5 7.0 6.5 6.0 5.5 5.0 4.5 4.0 3.5 3.0 2.5 2.0 1.5 1.0 0.5 0
f1 (ppm)

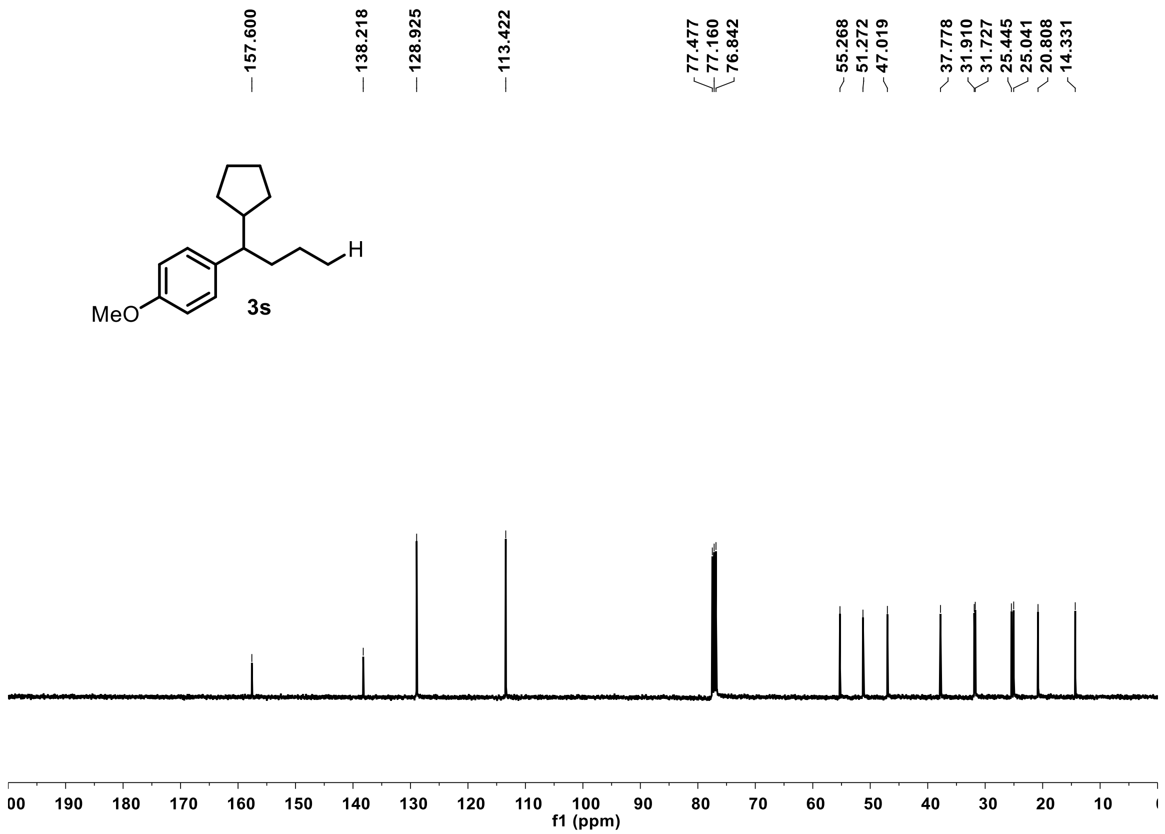
157.600
138.218
128.925
113.422
77.477
77.160
76.842
55.268
51.272
47.019
37.778
31.910
31.727
25.445
25.041
20.808
14.331
H
MeO
3s
f1 (ppm)

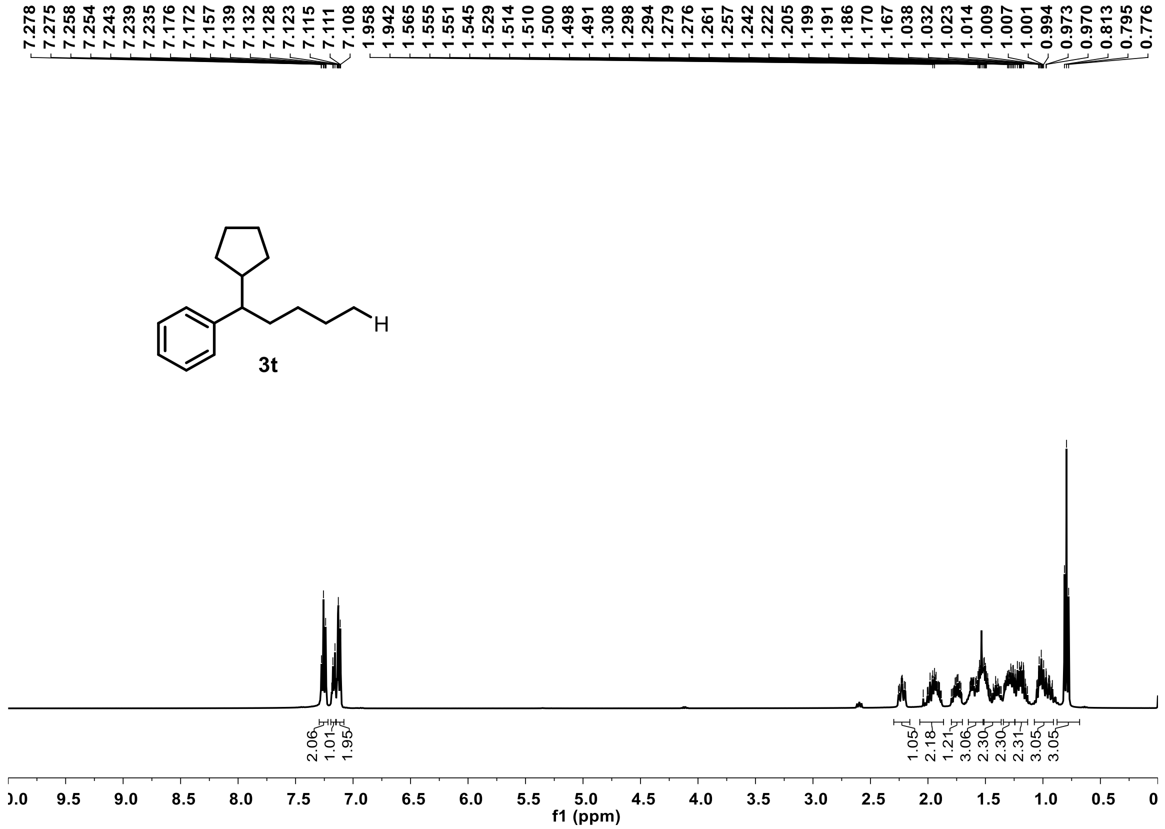
7.278
7.275
7.258
7.254
7.243
7.239
7.235
7.176
7.172
7.157
7.139
7.132
7.128
7.123
7.115
7.111
7.108
1.958
1.942
1.565
1.555
1.551
1.545
1.529
1.514
1.510
1.500
1.498
1.491
1.308
1.298
1.294
1.279
1.276
1.261
1.257
1.242
1.222
1.205
1.199
1.191
1.186
1.170
1.167
1.038
1.032
1.023
1.014
1.009
1.007
1.001
0.994
0.973
0.970
0.813
0.795
0.776
H
3t
2.06
1.01
1.95
1.05
2.18
1.21
3.06
2.30
2.30
2.31
3.05
3.05
f1 (ppm)

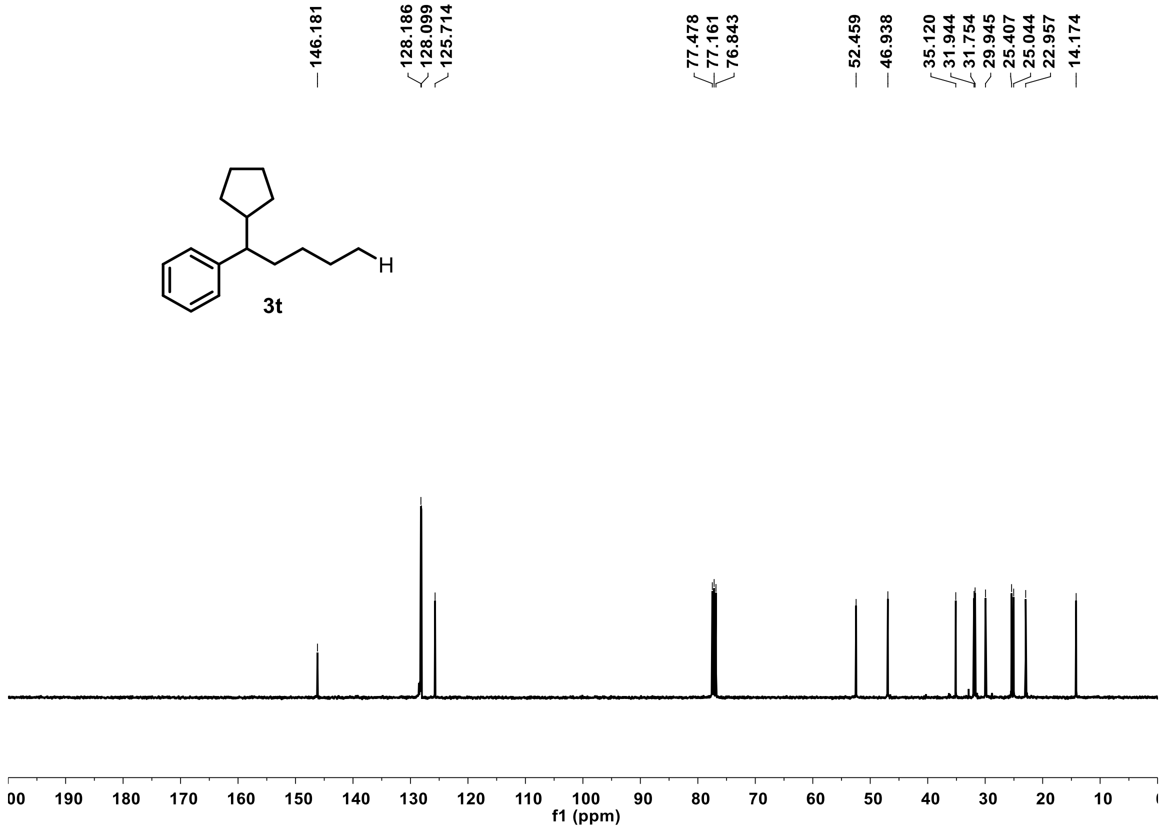
146.181
128.186
128.099
125.714
77.478
77.161
76.843
52.459
46.938
35.120
31.944
31.754
29.945
25.407
25.044
22.957
14.174
H
3t
00 190 180 170 160 150 140 130 120 110 100 90 80 70 60 50 40 30 20 10
f1 (ppm)

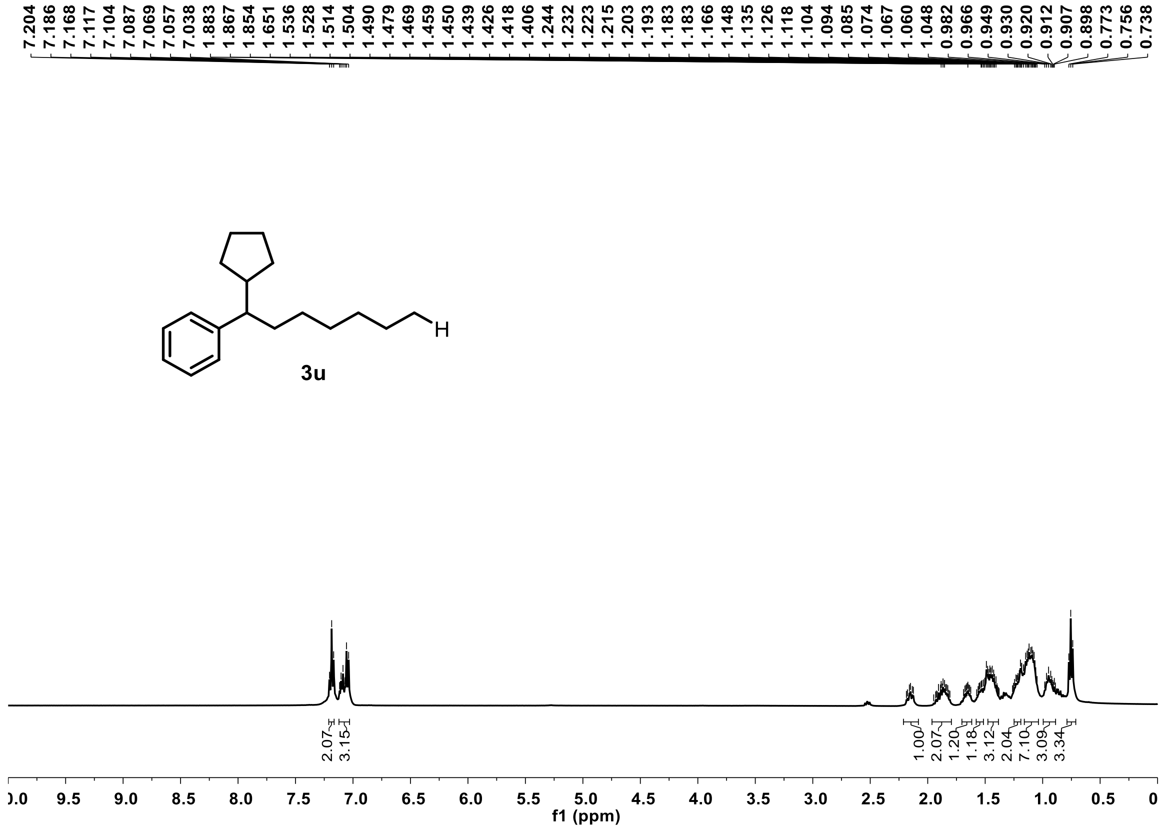
7.204
7.186
7.168
7.117
7.104
7.087
7.069
7.057
7.038
1.883
1.867
1.854
1.651
1.536
1.528
1.514
1.504
1.490
1.479
1.469
1.459
1.450
1.439
1.426
1.418
1.406
1.244
1.232
1.223
1.215
1.203
1.193
1.183
1.183
1.166
1.148
1.135
1.126
1.118
1.104
1.094
1.085
1.074
1.067
1.060
1.048
0.982
0.966
0.949
0.930
0.920
0.912
0.907
0.898
0.773
0.756
0.738
H
3u
2.07
3.15
1.00
2.07
1.20
1.18
3.12
2.04
7.10
3.09
3.34
.0 9.5 9.0 8.5 8.0 7.5 7.0 6.5 6.0 5.5 5.0 4.5 4.0 3.5 3.0 2.5 2.0 1.5 1.0 0.5 0
f1 (ppm)

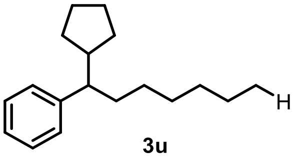
H

3u

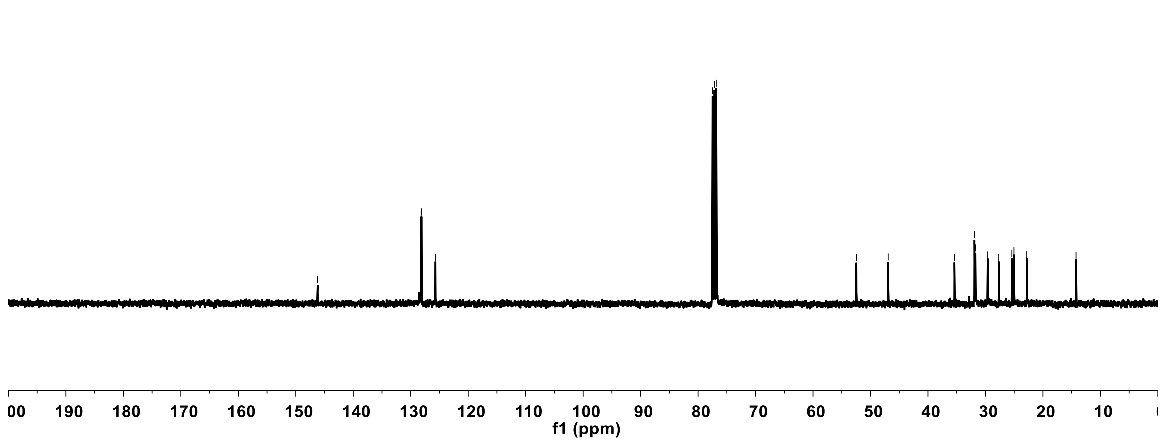
146.188
128.187
128.090
125.702
77.477
77.160
76.843
52.456
46.927
35.403
31.937
31.920
31.748
29.603
27.684
25.405
25.041
22.811
14.240
00 190 180 170 160 150 140 130 120 110 100 90 80 70 60 50 40 30 20 10
f1 (ppm)

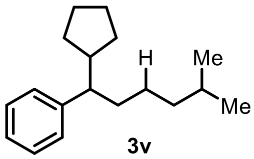
H
Me
Me

3v

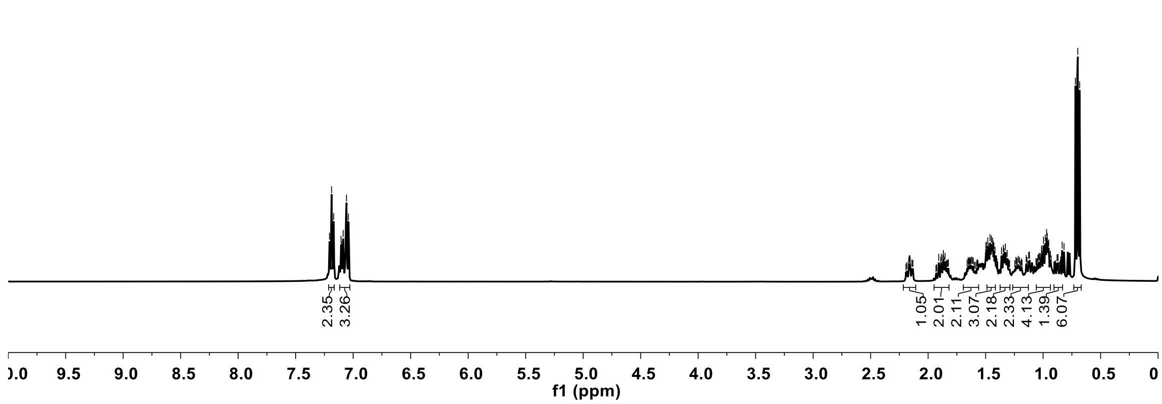
7.206
7.202
7.192
7.188
7.183
7.173
7.169
7.109
7.105
7.102
7.087
7.069
7.062
7.058
7.053
7.045
7.041
7.038
1.868
1.853
1.497
1.493
1.481
1.477
1.470
1.461
1.449
1.440
1.436
1.429
1.419
1.361
1.345
1.340
1.336
1.329
1.321
1.313
1.126
1.119
1.021
1.014
1.011
0.996
0.982
0.976
0.970
0.964
0.956
0.951
0.937
0.834
0.818
0.720
0.703
0.699
0.682
2.35
3.26
1.05
2.01
2.11
3.07
2.18
2.33
4.13
1.39
6.07
9.5 9.0 8.5 8.0 7.5 7.0 6.5 6.0 5.5 5.0 4.5 4.0 3.5 3.0 2.5 2.0 1.5 1.0 0.5 0
f1 (ppm)

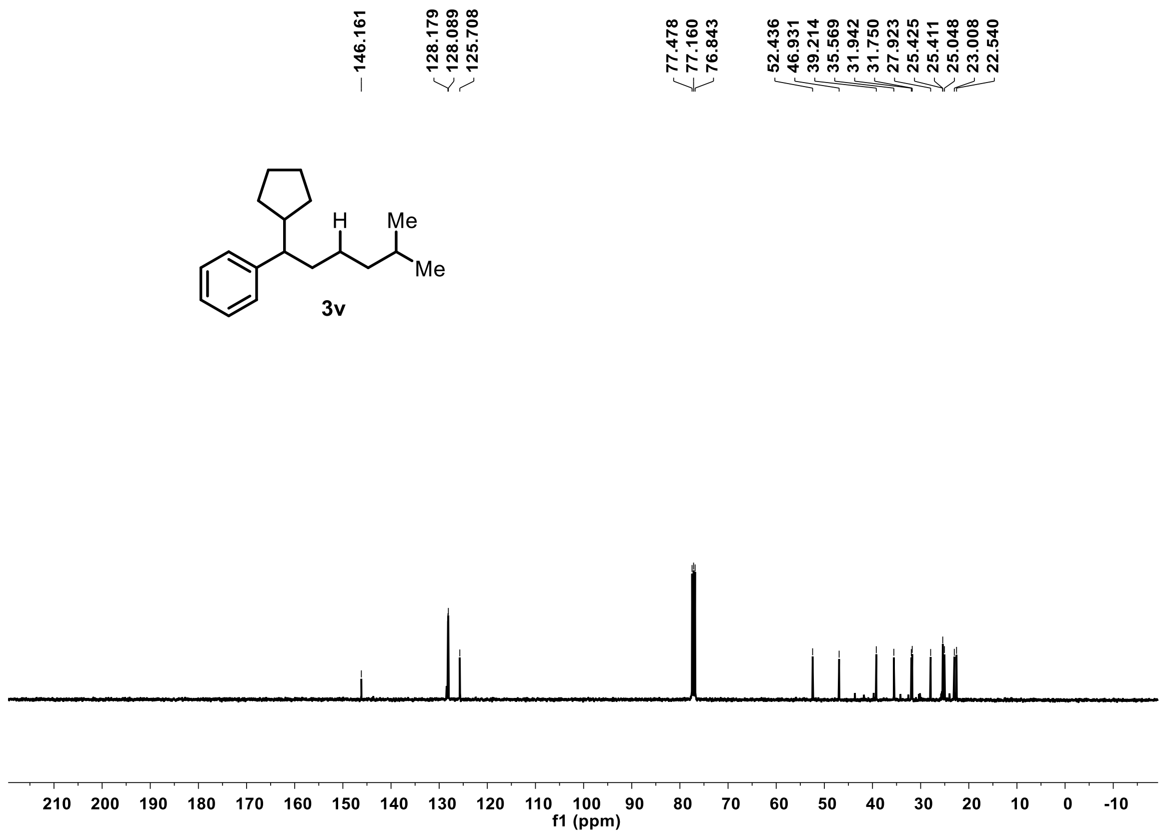
146.161
128.179
128.089
125.708
77.478
77.160
76.843
52.436
46.931
39.214
35.569
31.942
31.750
27.923
25.425
25.411
25.048
23.008
22.540
H
Me
Me
210 200 190 180 170 160 150 140 130 120 110 100 90 80 70 60 50 40 30 20 10 0 -10
f1 (ppm)

3v

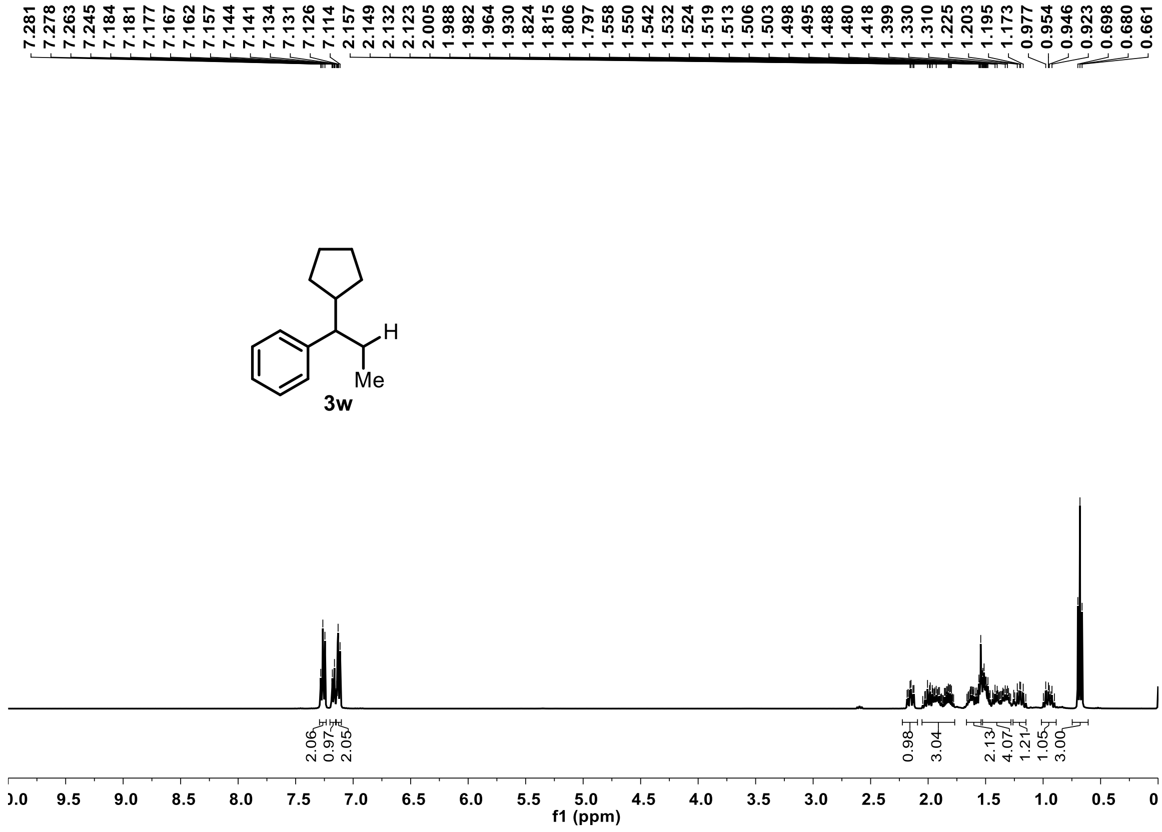
7.281
7.278
7.263
7.245
7.184
7.181
7.177
7.167
7.162
7.157
7.144
7.141
7.134
7.131
7.126
7.114
2.157
2.149
2.132
2.123
2.005
1.988
1.982
1.964
1.930
1.824
1.815
1.806
1.797
1.558
1.550
1.542
1.532
1.524
1.519
1.513
1.506
1.503
1.498
1.495
1.488
1.480
1.418
1.399
1.330
1.310
1.225
1.203
1.195
1.173
0.977
0.954
0.946
0.923
0.698
0.680
0.661
H
Me
2.06
0.97
2.05
0.98
3.04
2.13
4.07
1.21
1.05
3.00
9.5 9.0 8.5 8.0 7.5 7.0 6.5 6.0 5.5 5.0 4.5 4.0 3.5 3.0 2.5 2.0 1.5 1.0 0.5 0
f1 (ppm)

3w

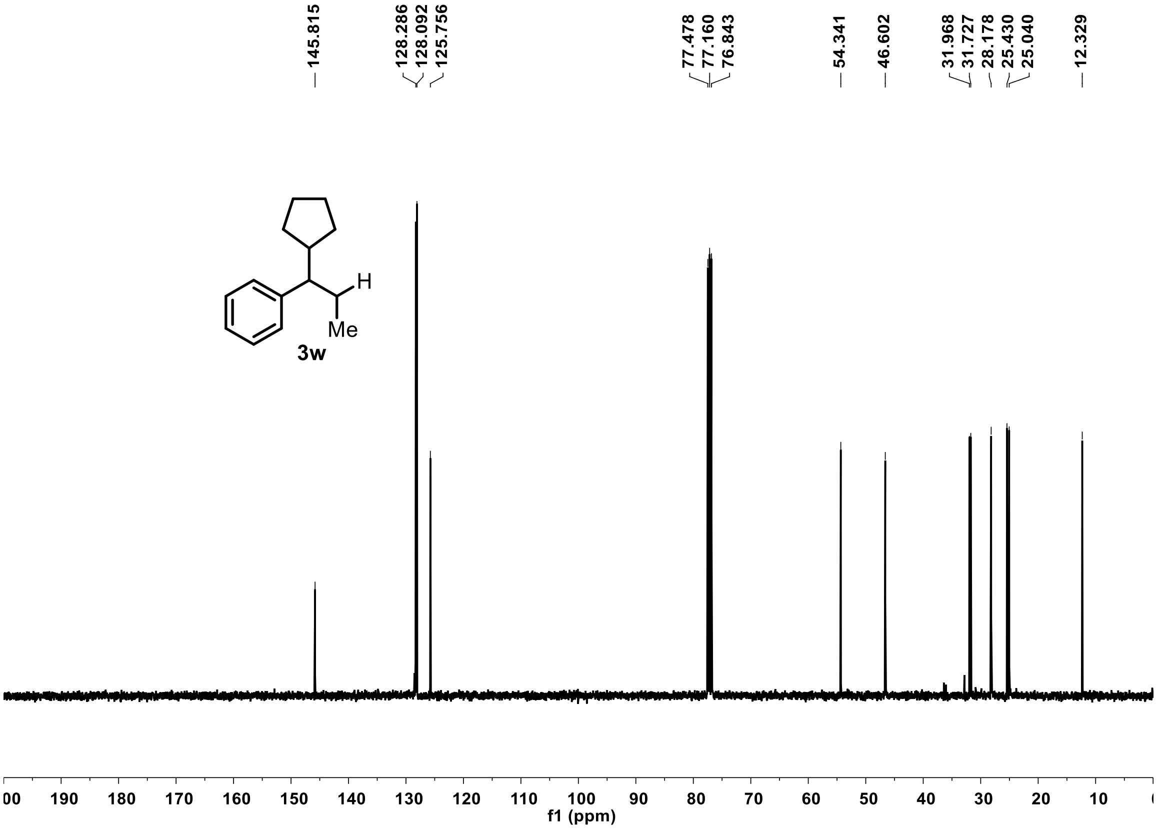
145.815
128.286
128.092
125.756
77.478
77.160
76.843
54.341
46.602
31.968
31.727
28.178
25.430
25.040
12.329
H
Me
3w
f1 (ppm)

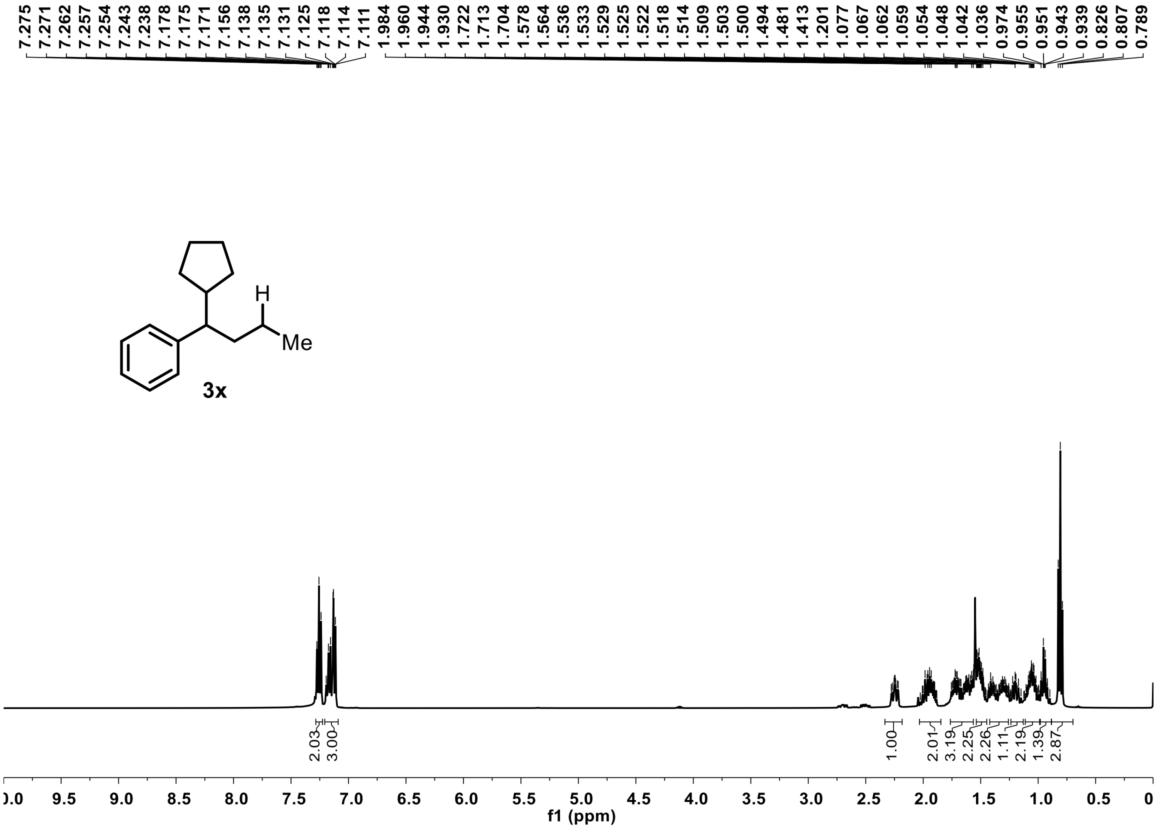
7.275
7.271
7.262
7.257
7.254
7.243
7.238
7.178
7.175
7.171
7.156
7.138
7.135
7.131
7.125
7.118
7.114
7.111
1.984
1.960
1.944
1.930
1.722
1.713
1.704
1.578
1.564
1.536
1.533
1.529
1.525
1.522
1.518
1.514
1.509
1.503
1.500
1.494
1.481
1.413
1.201
1.077
1.067
1.062
1.059
1.054
1.048
1.042
1.036
0.974
0.955
0.951
0.943
0.939
0.826
0.807
0.789
H
Me
3x
2.03
3.00
1.00
2.01
3.19
2.25
2.26
1.11
2.19
1.39
2.87
f1 (ppm)

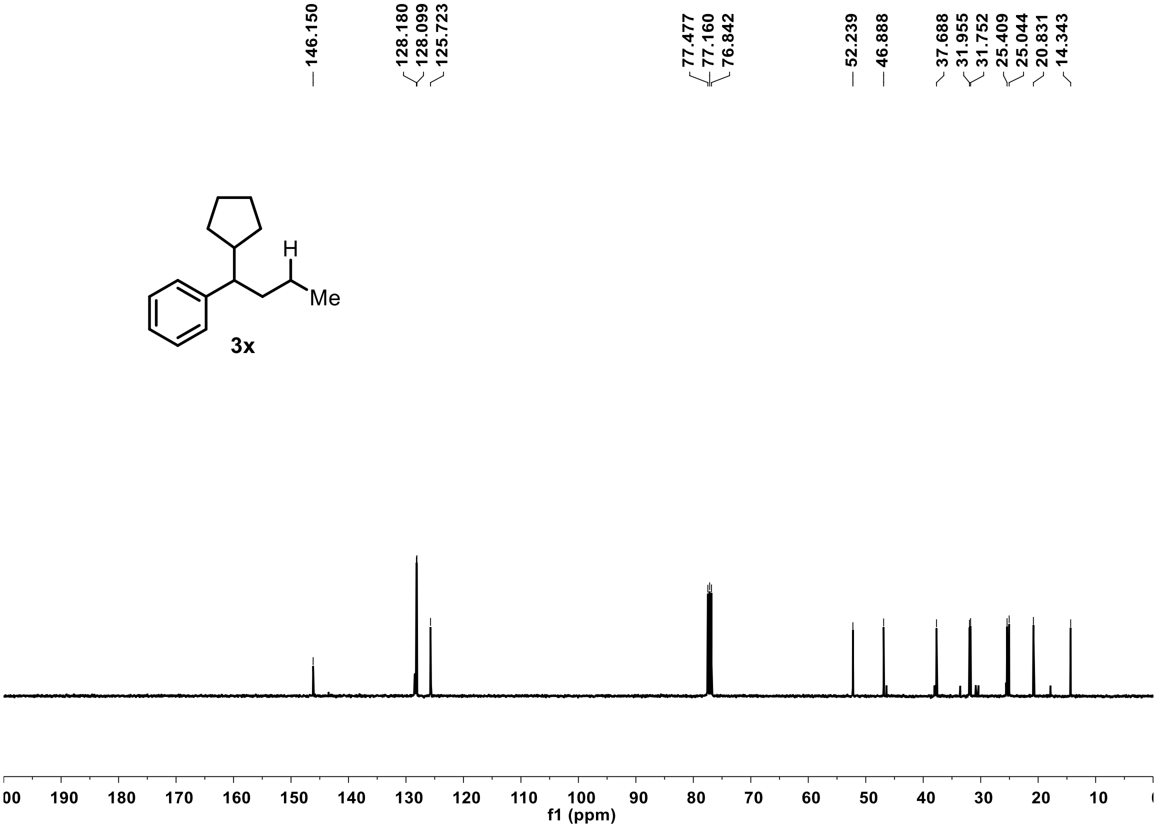
146.150
128.180
128.099
125.723
77.477
77.160
76.842
52.239
46.888
37.688
31.955
31.752
25.409
25.044
20.831
14.343
H
Me
3x
00 190 180 170 160 150 140 130 120 110 100 90 80 70 60 50 40 30 20 10
f1 (ppm)

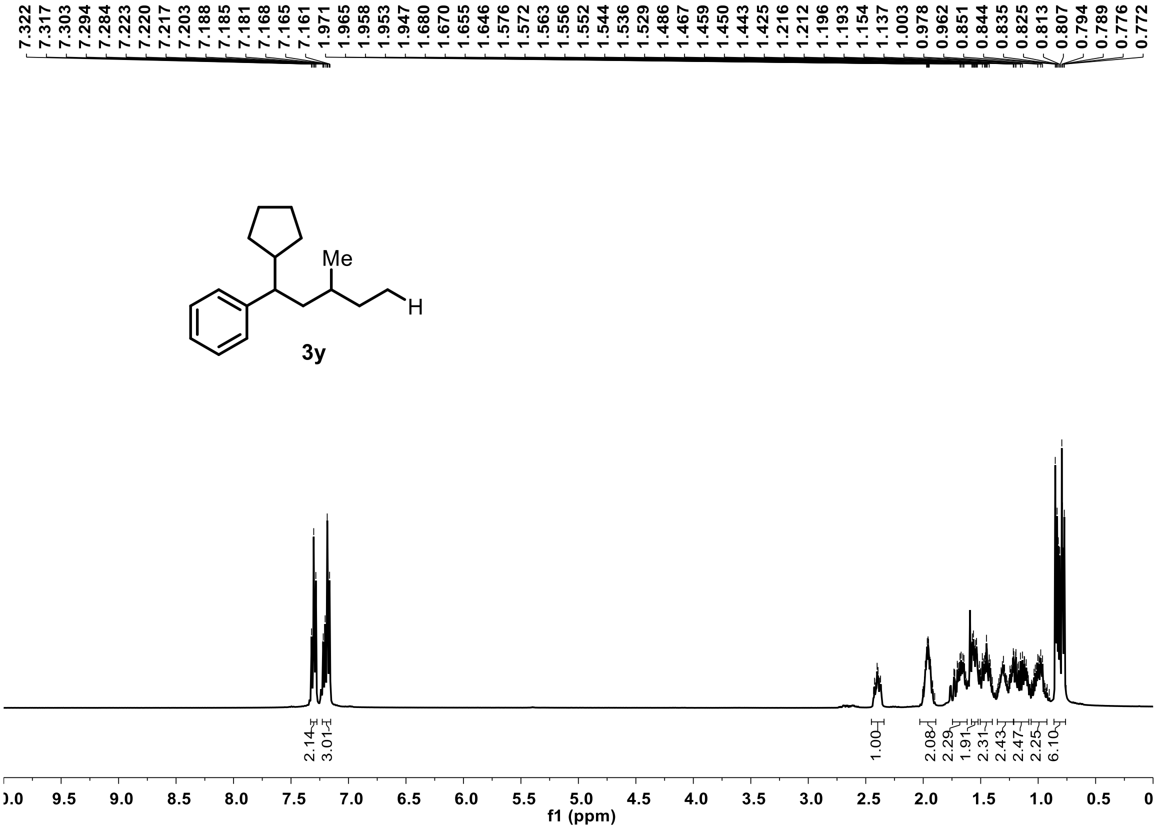
7.322
7.317
7.303
7.294
7.284
7.223
7.220
7.217
7.203
7.188
7.185
7.181
7.168
7.165
7.161
1.971
1.965
1.958
1.953
1.947
1.680
1.670
1.655
1.646
1.576
1.572
1.563
1.556
1.552
1.544
1.536
1.529
1.486
1.467
1.459
1.450
1.443
1.425
1.216
1.212
1.196
1.193
1.154
1.137
1.003
0.978
0.962
0.851
0.844
0.835
0.825
0.813
0.807
0.794
0.789
0.776
0.772
Me
H
3y
2.14
3.01
1.00
2.08
2.29
1.91
2.31
2.43
2.47
2.25
6.10
9.5 9.0 8.5 8.0 7.5 7.0 6.5 6.0 5.5 5.0 4.5 4.0 3.5 3.0 2.5 2.0 1.5 1.0 0.5 0
f1 (ppm)

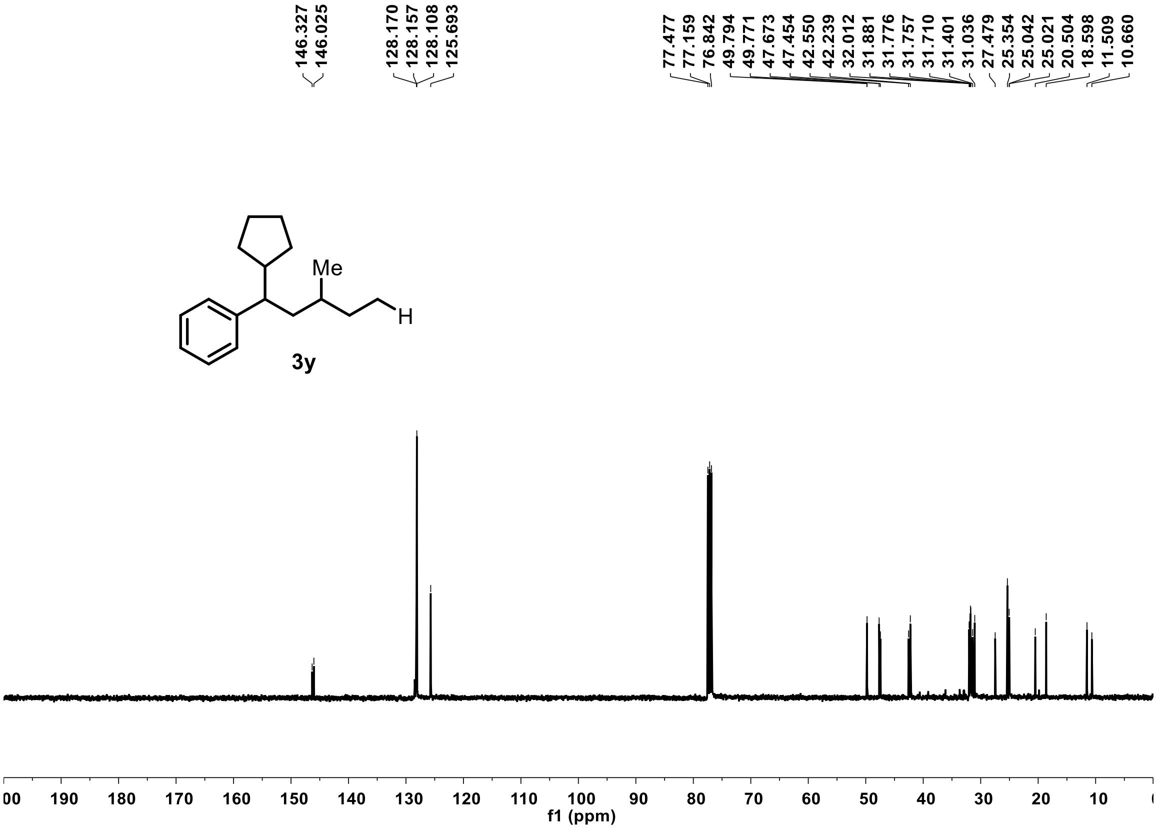
146.327
146.025
128.170
128.157
128.108
125.693
77.477
77.159
76.842
49.794
49.771
47.673
47.454
42.550
42.239
32.012
31.881
31.776
31.757
31.710
31.401
31.036
27.479
25.354
25.042
25.021
20.504
18.598
11.509
10.660
Me
H
3y
00 190 180 170 160 150 140 130 120 110 100 90 80 70 60 50 40 30 20 10
f1 (ppm)

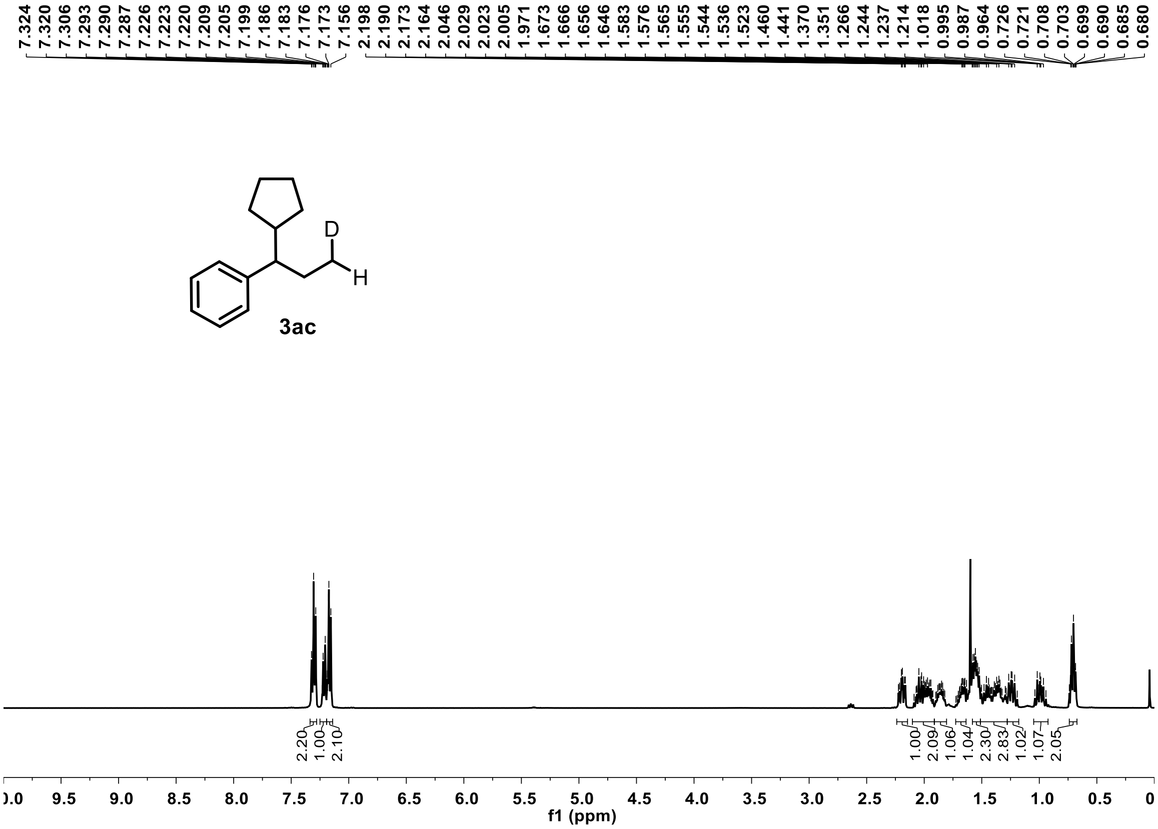
7.324
7.320
7.306
7.293
7.290
7.287
7.226
7.223
7.220
7.209
7.205
7.199
7.186
7.183
7.176
7.173
7.156
2.198
2.190
2.173
2.164
2.046
2.029
2.023
2.005
1.971
1.673
1.666
1.656
1.646
1.583
1.576
1.565
1.555
1.544
1.536
1.523
1.460
1.441
1.370
1.351
1.266
1.244
1.237
1.214
1.018
0.995
0.987
0.964
0.726
0.721
0.708
0.703
0.699
0.690
0.685
0.680
D
H
3ac
2.20
1.00
2.10
1.00
2.09
1.06
1.04
2.30
2.83
1.02
1.07
2.05
10.0 9.5 9.0 8.5 8.0 7.5 7.0 6.5 6.0 5.5 5.0 4.5 4.0 3.5 3.0 2.5 2.0 1.5 1.0 0.5 0
f1 (ppm)

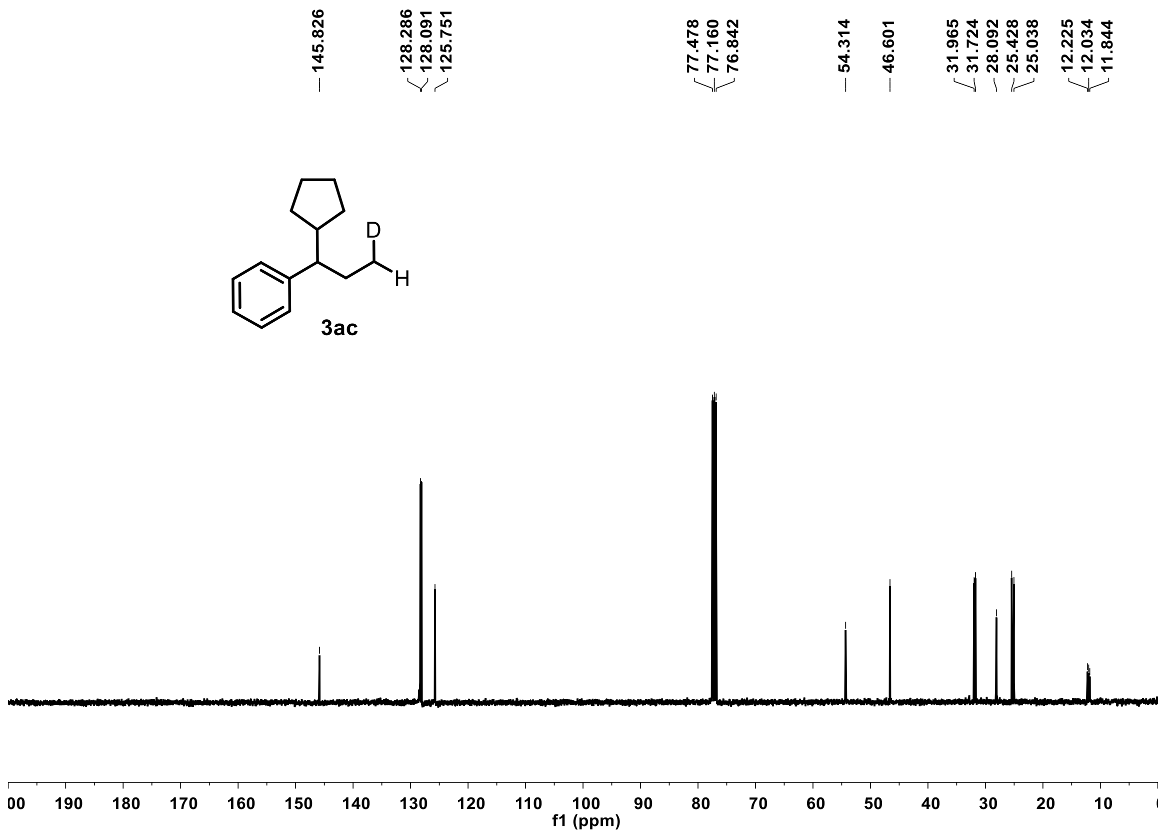
145.826
128.286
128.091
125.751
77.478
77.160
76.842
54.314
46.601
31.965
31.724
28.092
25.428
25.038
12.225
12.034
11.844
D
H
3ac
f1 (ppm)

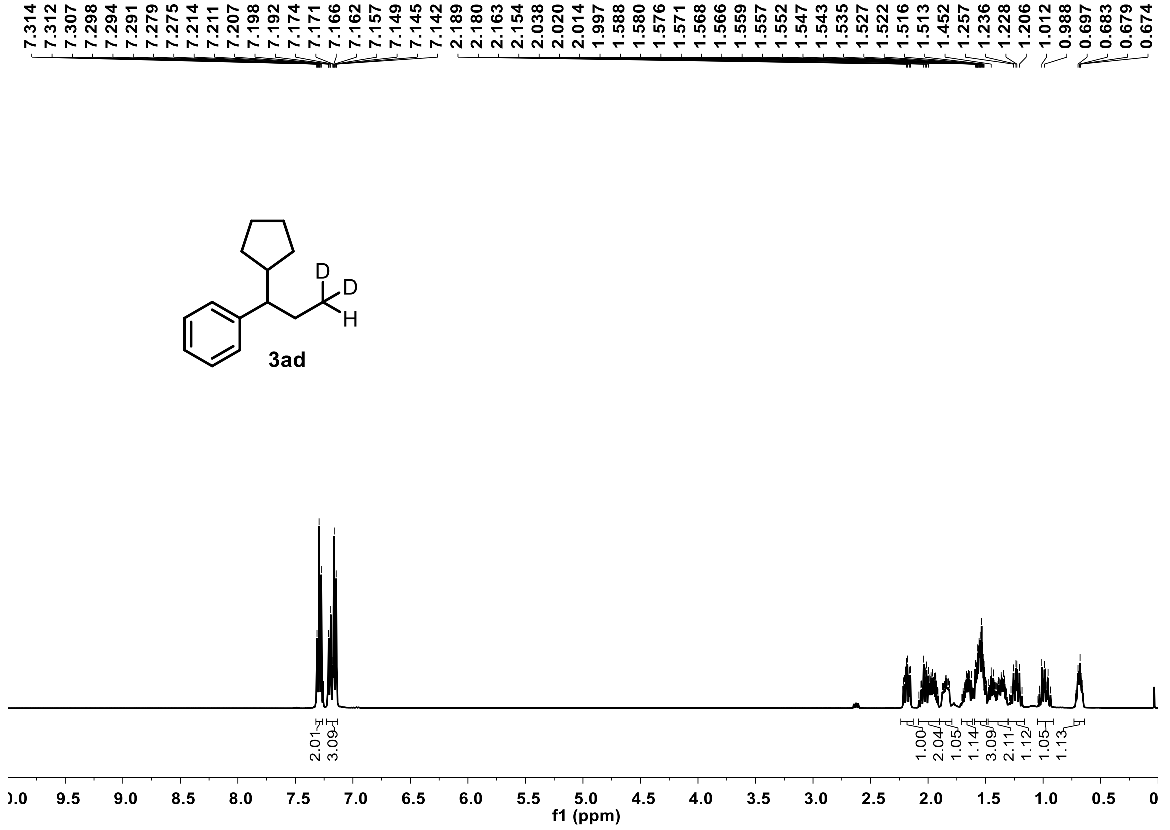
7.314
7.312
7.307
7.298
7.294
7.291
7.279
7.275
7.214
7.211
7.207
7.198
7.192
7.174
7.171
7.166
7.162
7.157
7.149
7.145
7.142
2.189
2.180
2.163
2.154
2.038
2.020
2.014
1.997
1.588
1.580
1.576
1.571
1.568
1.566
1.559
1.557
1.552
1.547
1.543
1.535
1.527
1.522
1.516
1.513
1.452
1.257
1.236
1.228
1.206
1.012
0.988
0.697
0.683
0.679
0.674
D
D
H
3ad
2.01
3.09
1.00
2.04
1.05
1.14
3.09
2.11
1.12
1.05
1.13
f1 (ppm)

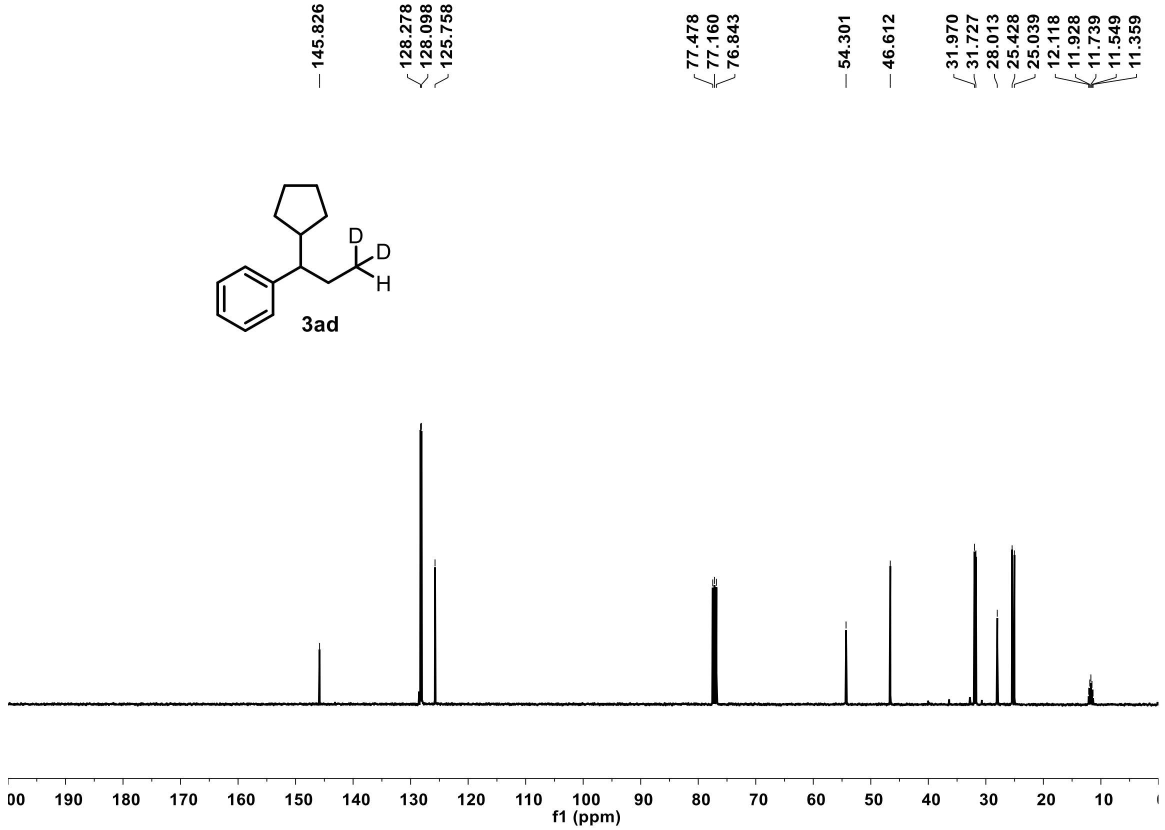
145.826
128.278
128.098
125.758
77.478
77.160
76.843
54.301
46.612
31.970
31.727
28.013
25.428
25.039
12.118
11.928
11.739
11.549
11.359
3ad
f1 (ppm)

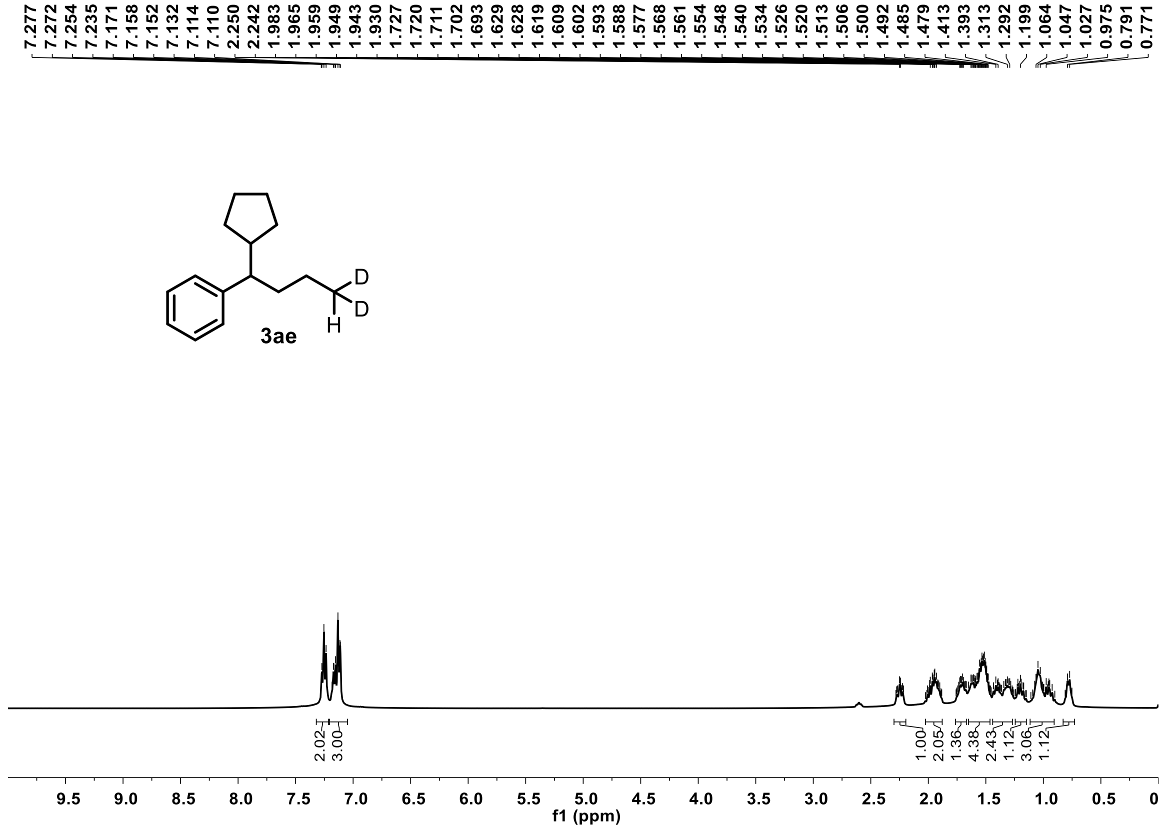
7.277
7.272
7.254
7.235
7.171
7.158
7.152
7.132
7.114
7.110
2.250
2.242
1.983
1.965
1.959
1.949
1.943
1.930
1.727
1.720
1.711
1.702
1.693
1.628
1.619
1.609
1.602
1.593
1.588
1.577
1.568
1.561
1.554
1.548
1.540
1.534
1.526
1.520
1.513
1.506
1.500
1.492
1.485
1.479
1.413
1.393
1.313
1.292
1.199
1.064
1.047
1.027
0.975
0.791
0.771
3ae
2.02
3.00
1.00
2.05
1.36
4.38
2.43
1.12
3.06
1.12
f1 (ppm)

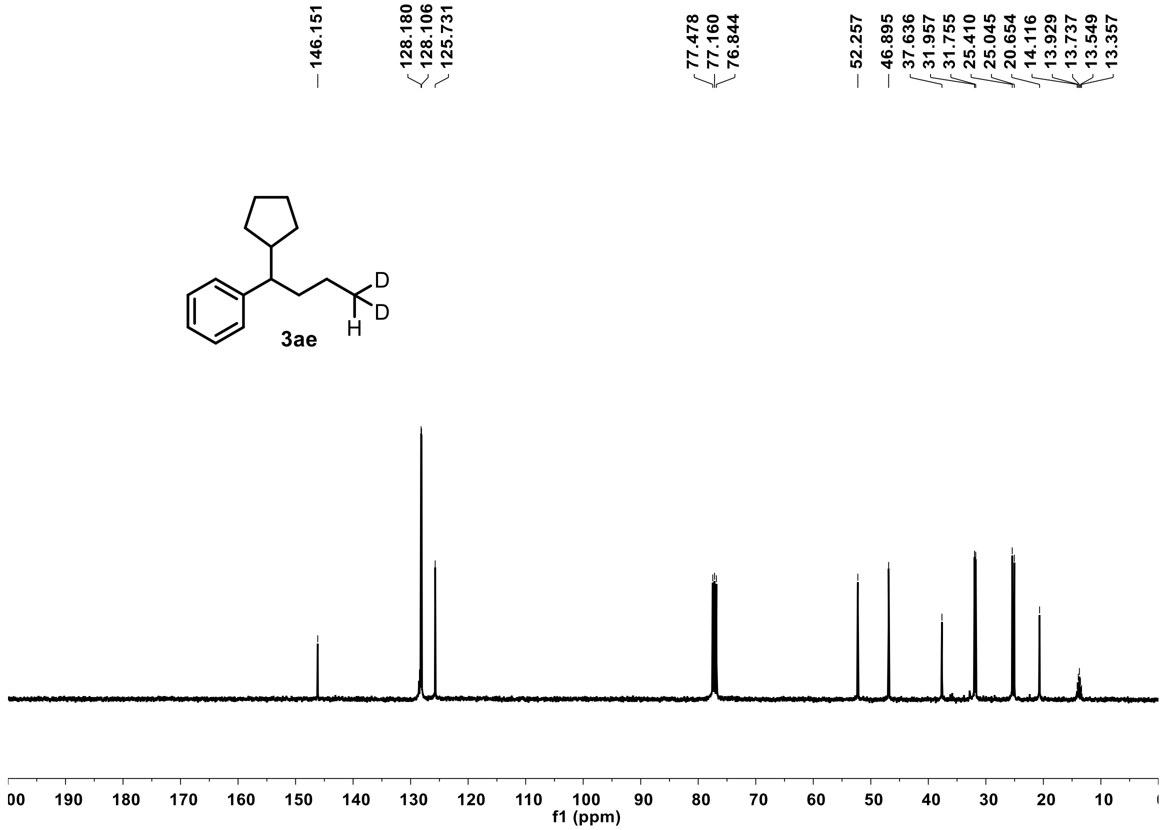
146.151
128.180
128.106
125.731
77.478
77.160
76.844
52.257
46.895
37.636
31.957
31.755
25.410
25.045
20.654
14.116
13.929
13.737
13.549
13.357
D
D
H
3ae
f1 (ppm)

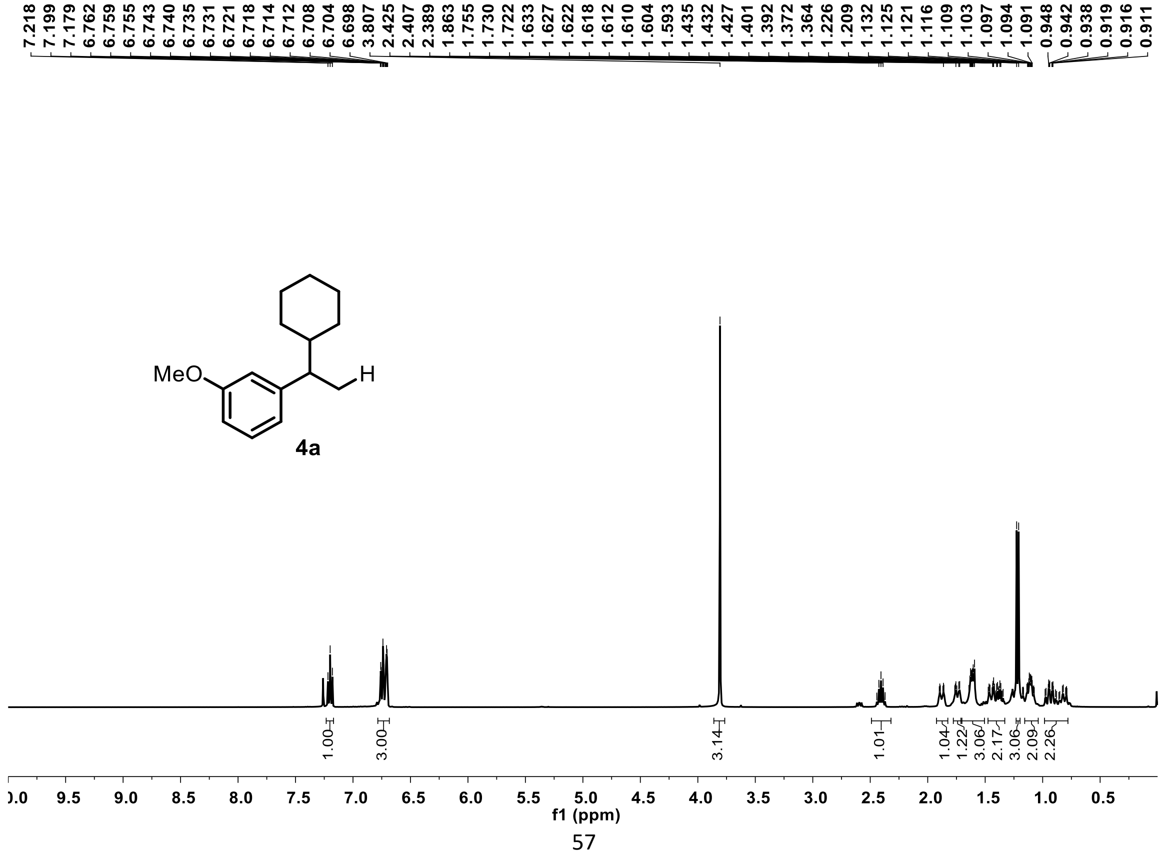
7.218
7.199
7.179
6.762
6.759
6.755
6.743
6.740
6.735
6.731
6.721
6.718
6.714
6.712
6.708
6.704
6.698
3.807
2.425
2.407
2.389
1.863
1.755
1.730
1.722
1.633
1.627
1.622
1.618
1.612
1.610
1.604
1.593
1.435
1.432
1.427
1.401
1.392
1.372
1.364
1.226
1.209
1.132
1.125
1.121
1.116
1.109
1.103
1.097
1.094
1.091
0.948
0.942
0.938
0.919
0.916
0.911
MeO
H
4a
1.00
3.00
3.14
1.01
1.04
1.22
3.06
2.17
3.06
2.09
2.26
f1 (ppm)

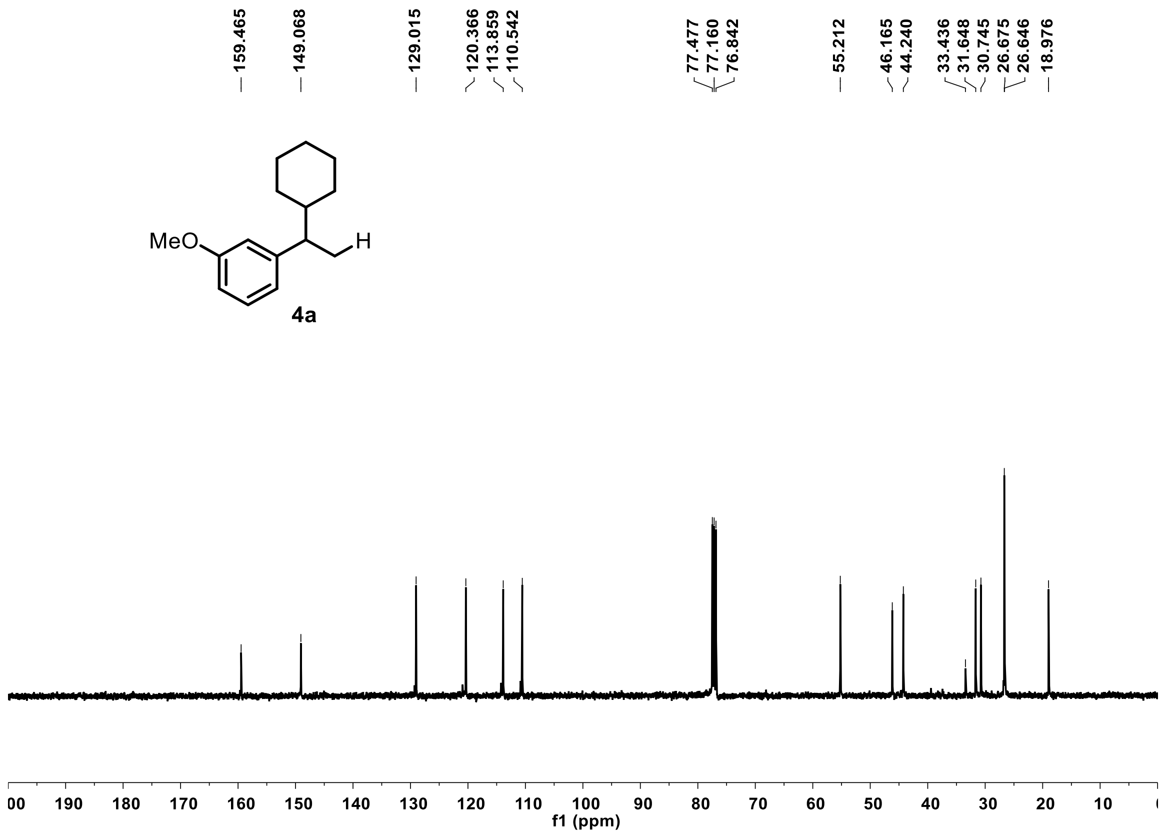

159.465
149.068
129.015
120.366
113.859
110.542
77.477
77.160
76.842
55.212
46.165
44.240
33.436
31.648
30.745
26.675
26.646
18.976
MeO
H
f1 (ppm)

4a

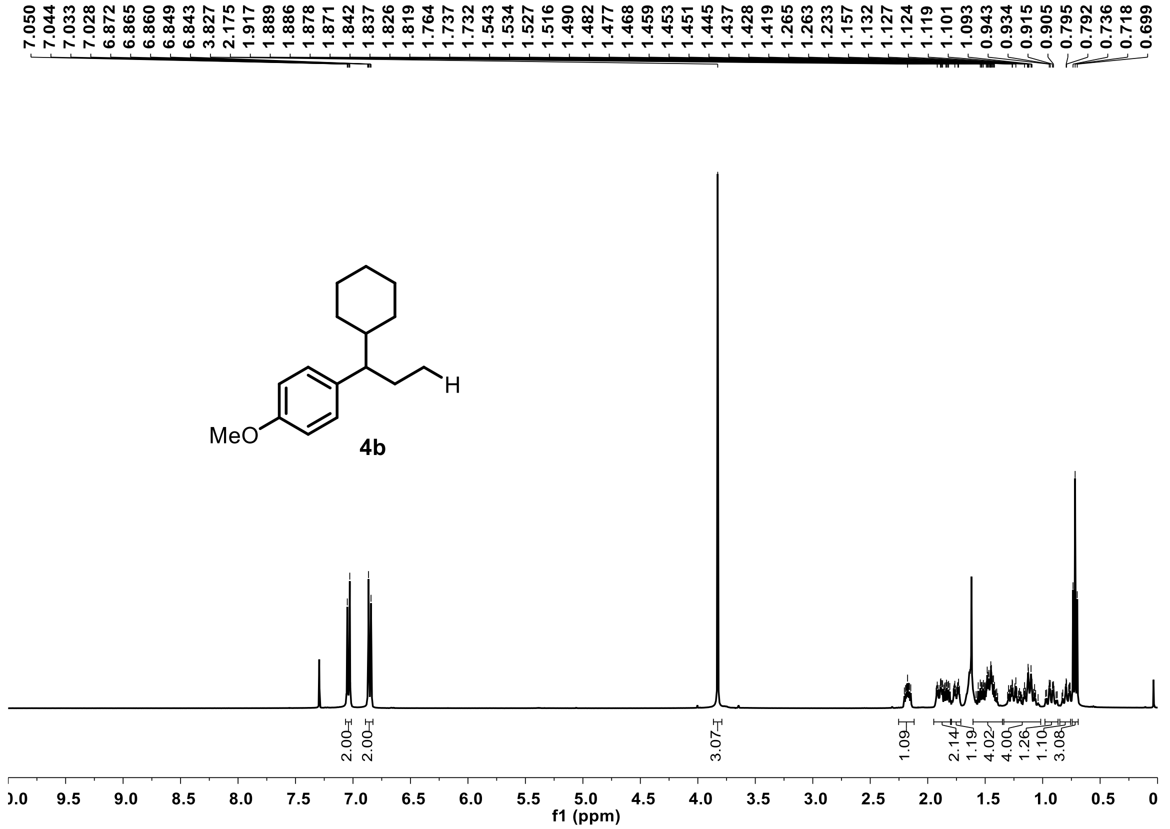

7.050 7.044 7.033 7.028 6.872 6.865 6.860 6.849 6.843 3.827 2.175 1.917 1.889 1.886 1.878 1.871 1.842 1.837 1.826 1.819 1.764 1.737 1.732 1.543 1.534 1.527 1.516 1.490 1.482 1.477 1.468 1.459 1.453 1.451 1.445 1.437 1.428 1.419 1.265 1.263 1.233 1.157 1.132 1.127 1.124 1.119 1.101 1.093 0.943 0.934 0.915 0.905 0.795 0.792 0.736 0.718 0.699
MeO
H
2.00 2.00 3.07 1.09 2.14 1.19 4.02 4.00 1.26 1.10 3.08
f1 (ppm)

4b

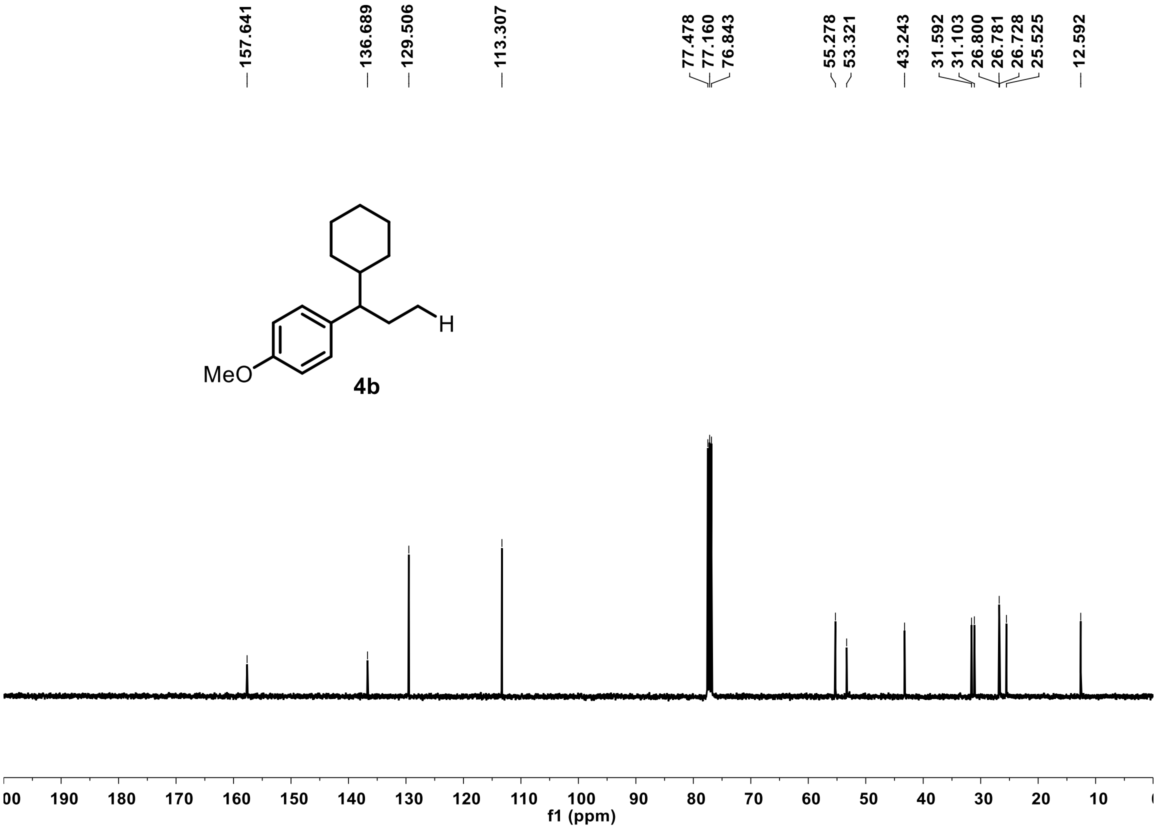
157.641
136.689
129.506
113.307
77.478
77.160
76.843
55.278
53.321
43.243
31.592
31.103
26.800
26.781
26.728
25.525
12.592
MeO
H
4b
f1 (ppm)

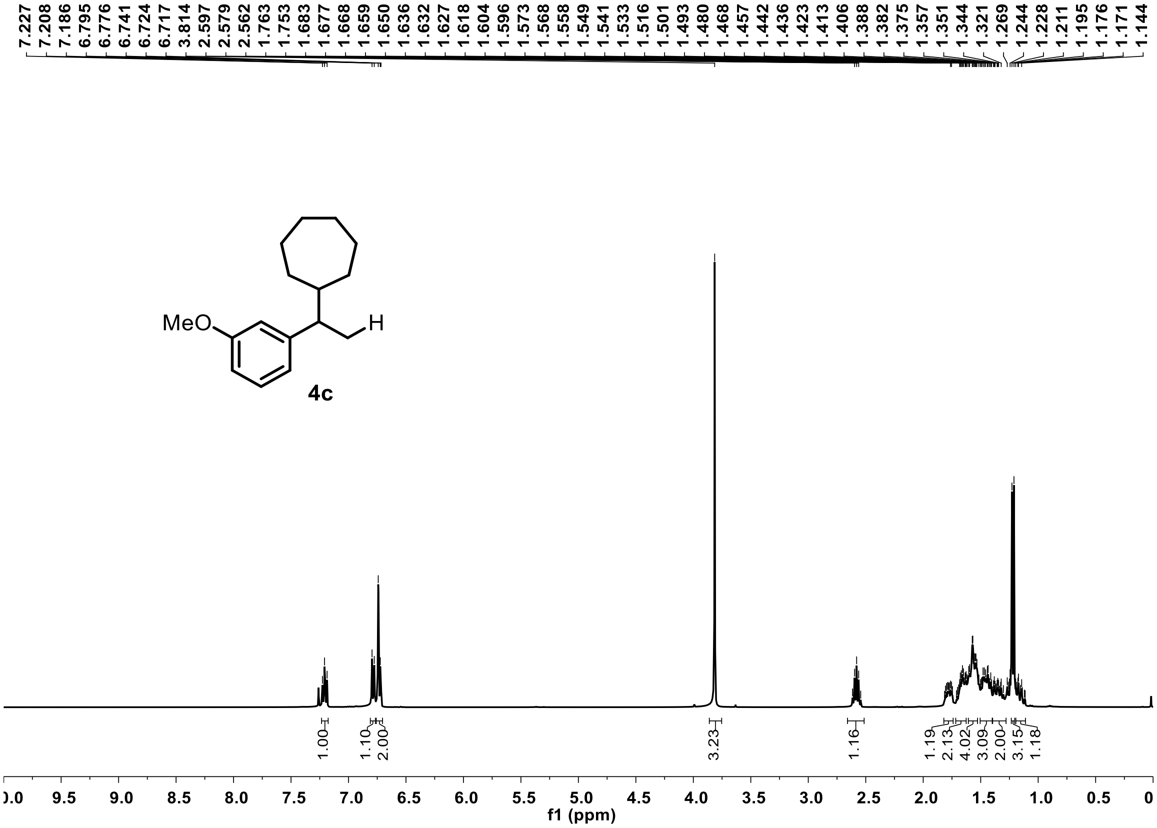
7.227
7.208
7.186
6.795
6.776
6.741
6.724
6.717
3.814
2.597
2.579
2.562
1.763
1.753
1.683
1.677
1.668
1.659
1.650
1.636
1.632
1.627
1.618
1.604
1.596
1.573
1.568
1.558
1.549
1.541
1.533
1.516
1.501
1.493
1.480
1.468
1.457
1.442
1.436
1.423
1.413
1.406
1.388
1.382
1.375
1.357
1.351
1.344
1.321
1.269
1.244
1.228
1.211
1.195
1.176
1.171
1.144
MeO
H
4c
1.00
1.10
2.00
3.23
1.16
1.19
2.13
4.02
3.09
2.00
3.15
1.18
f1 (ppm)

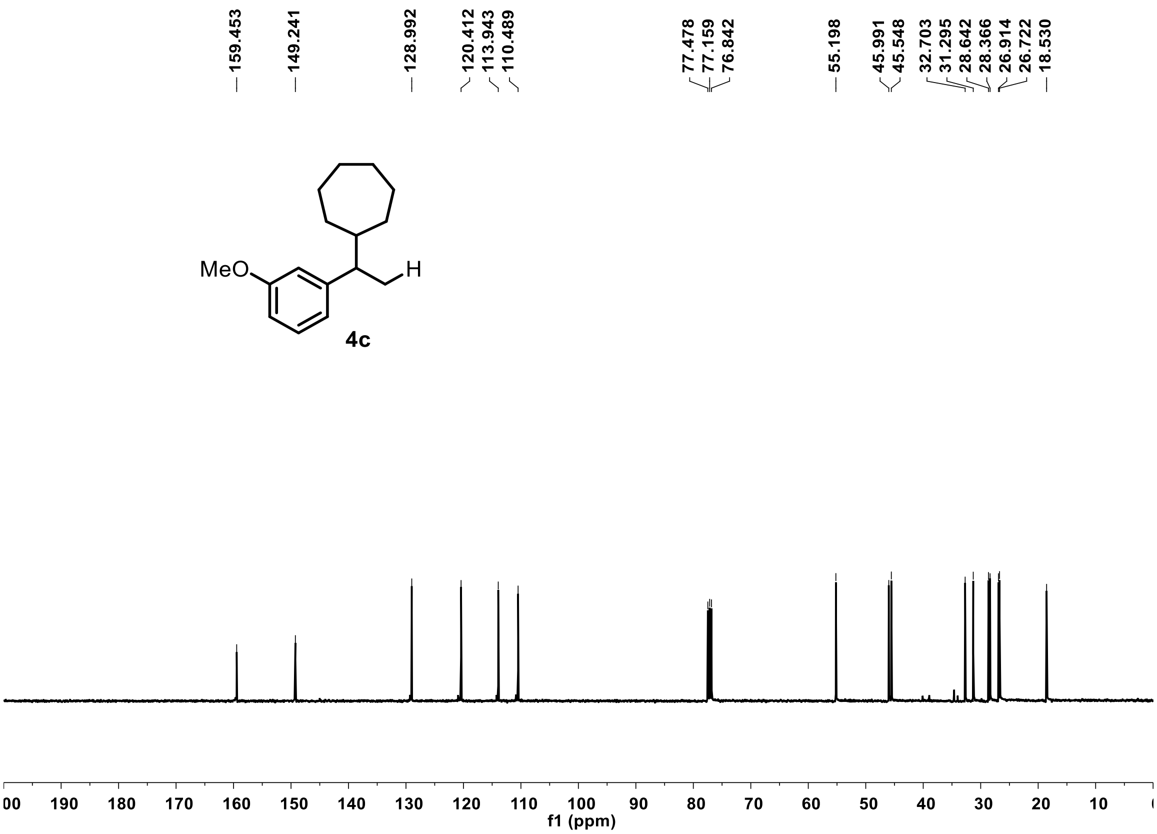
159.453
149.241
128.992
120.412
113.943
110.489
77.478
77.159
76.842
55.198
45.991
45.548
32.703
31.295
28.642
28.366
26.914
26.722
18.530
MeO
H
00 190 180 170 160 150 140 130 120 110 100 90 80 70 60 50 40 30 20 10
f1 (ppm)

4c

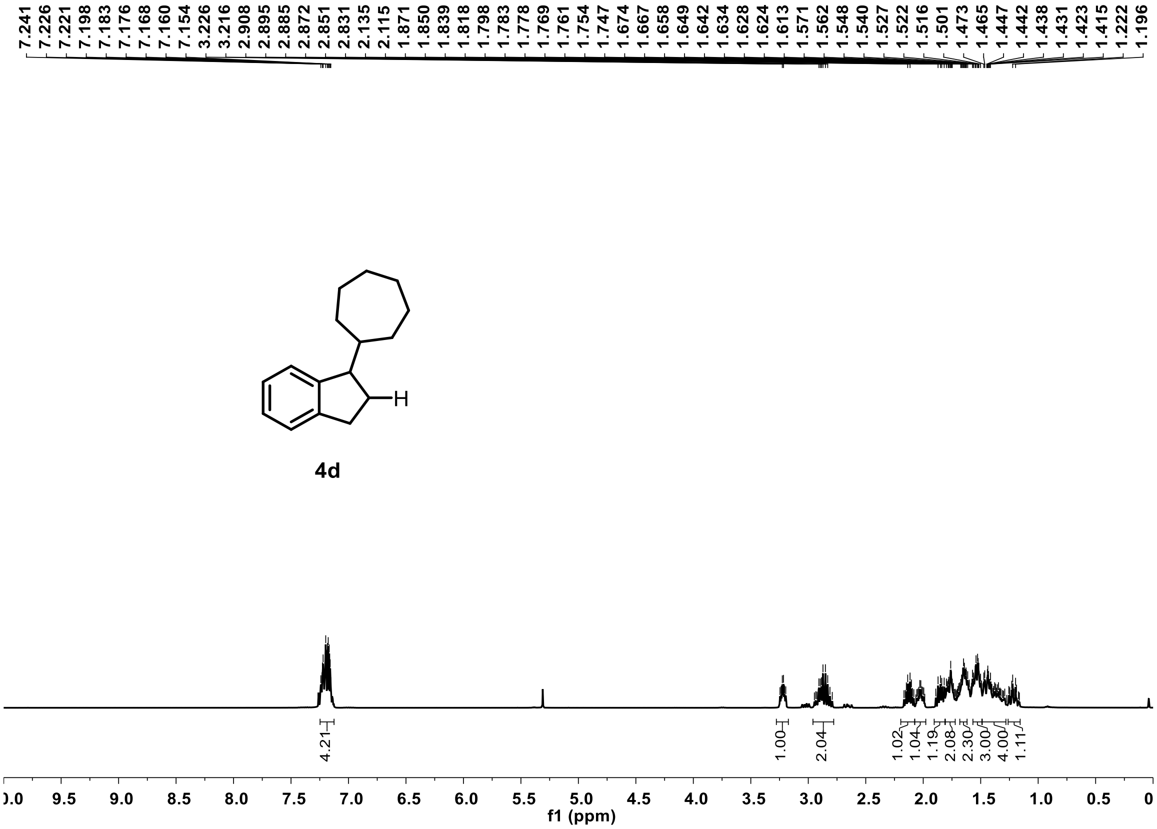
7.241
7.226
7.221
7.198
7.183
7.176
7.168
7.160
7.154
3.226
3.216
2.908
2.895
2.885
2.872
2.851
2.831
2.135
2.115
1.871
1.850
1.839
1.818
1.798
1.783
1.778
1.769
1.761
1.754
1.747
1.674
1.667
1.658
1.649
1.642
1.634
1.628
1.624
1.613
1.571
1.562
1.548
1.540
1.527
1.522
1.516
1.501
1.473
1.465
1.447
1.442
1.438
1.431
1.423
1.415
1.222
1.196
H
4.21
1.00
2.04
1.02
1.04
1.19
2.08
2.30
3.00
4.00
1.11
9.5 9.0 8.5 8.0 7.5 7.0 6.5 6.0 5.5 5.0 4.5 4.0 3.5 3.0 2.5 2.0 1.5 1.0 0.5 0
f1 (ppm)

4d

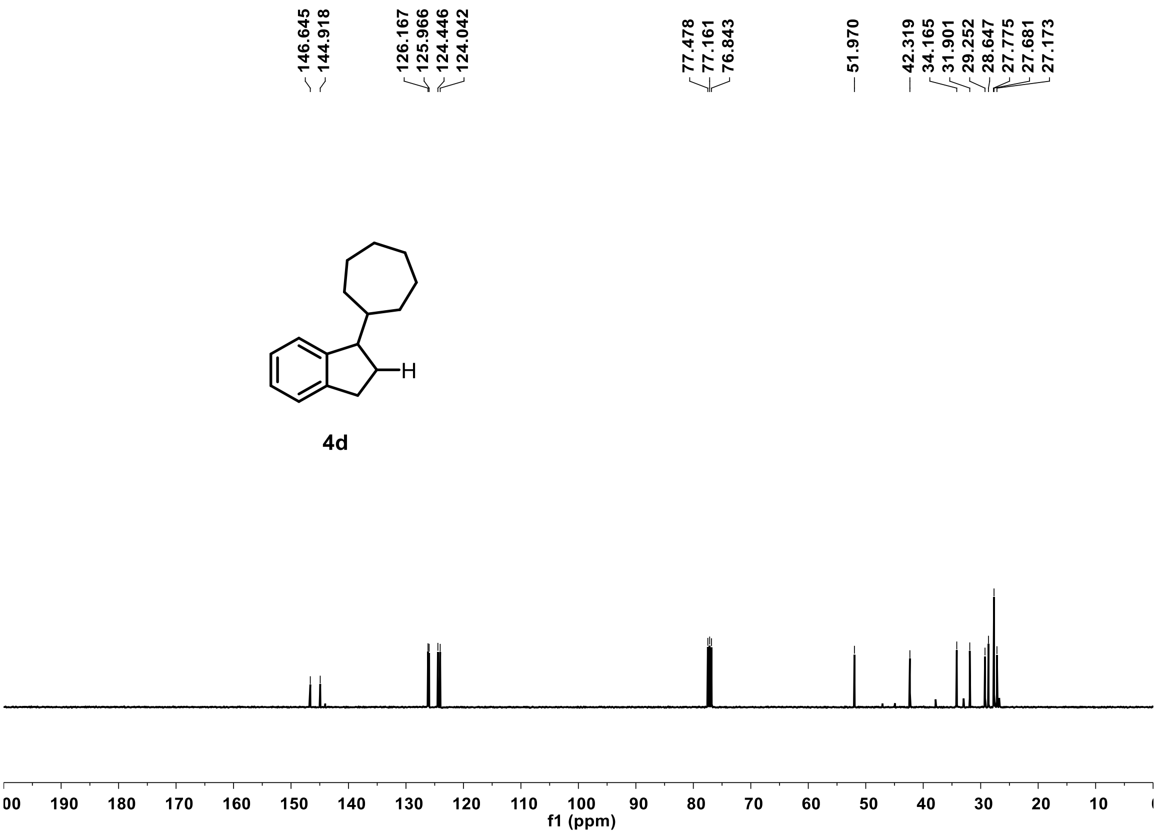
146.645
144.918
126.167
125.966
124.446
124.042
77.478
77.161
76.843
51.970
42.319
34.165
31.901
29.252
28.647
27.775
27.681
27.173
H
4d
00 190 180 170 160 150 140 130 120 110 100 90 80 70 60 50 40 30 20 10
f1 (ppm)

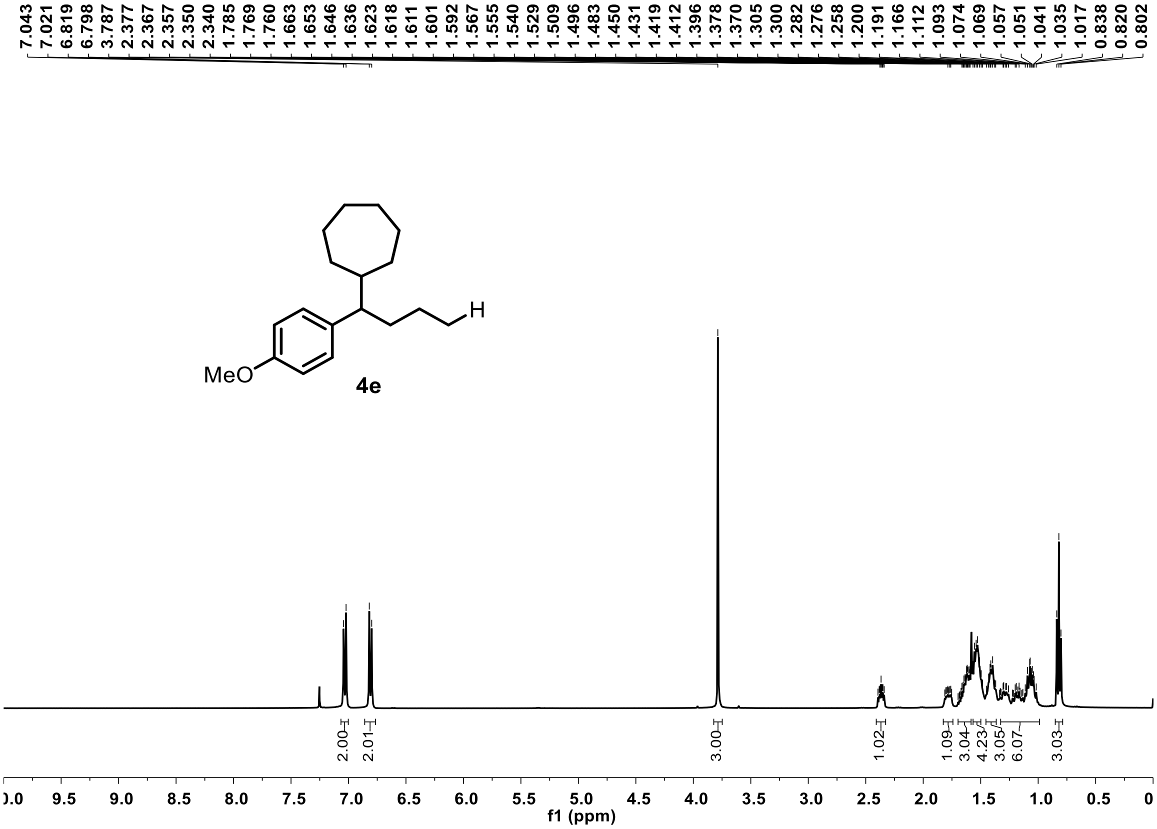
7.043
7.021
6.819
6.798
3.787
2.377
2.367
2.357
2.350
2.340
1.785
1.769
1.760
1.663
1.653
1.646
1.636
1.623
1.618
1.611
1.601
1.592
1.567
1.555
1.540
1.529
1.509
1.496
1.483
1.450
1.431
1.419
1.412
1.396
1.378
1.370
1.305
1.300
1.282
1.276
1.258
1.200
1.191
1.166
1.112
1.093
1.074
1.069
1.057
1.051
1.041
1.035
1.017
0.838
0.820
0.802
H
MeO
4e
2.00
2.01
3.00
1.02
1.09
3.04
4.23
3.05
6.07
3.03
0.0 9.5 9.0 8.5 8.0 7.5 7.0 6.5 6.0 5.5 5.0 4.5 4.0 3.5 3.0 2.5 2.0 1.5 1.0 0.5 0
f1 (ppm)

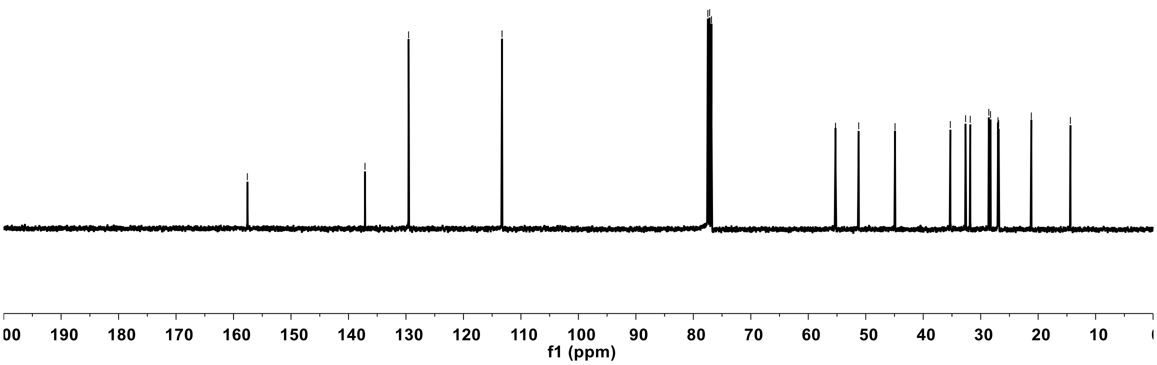
157.600
137.130
129.537
113.283
77.479
77.161
76.843
55.260
51.228
44.904
35.284
32.627
31.815
28.589
28.291
26.983
26.864
21.195
14.398
f1 (ppm)

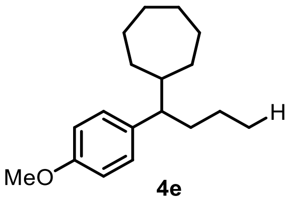
MeO
H

4e

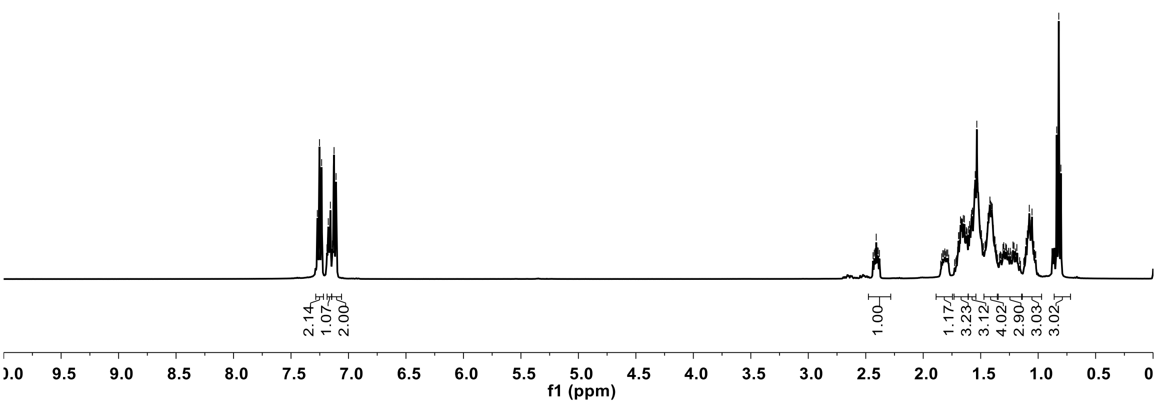
7.271
7.267
7.252
7.234
7.176
7.172
7.158
7.129
7.125
7.108
2.409
1.693
1.683
1.676
1.670
1.666
1.659
1.651
1.642
1.634
1.625
1.619
1.605
1.596
1.592
1.582
1.579
1.571
1.562
1.556
1.547
1.534
1.523
1.512
1.504
1.493
1.448
1.441
1.436
1.430
1.425
1.420
1.413
1.405
1.399
1.390
1.382
1.376
1.095
1.087
1.077
1.061
1.054
0.839
0.821
0.802
2.14
1.07
2.00
1.00
1.17
3.23
3.12
4.02
2.90
3.03
3.02
f1 (ppm)

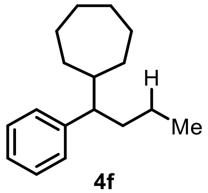
H
Me

4f

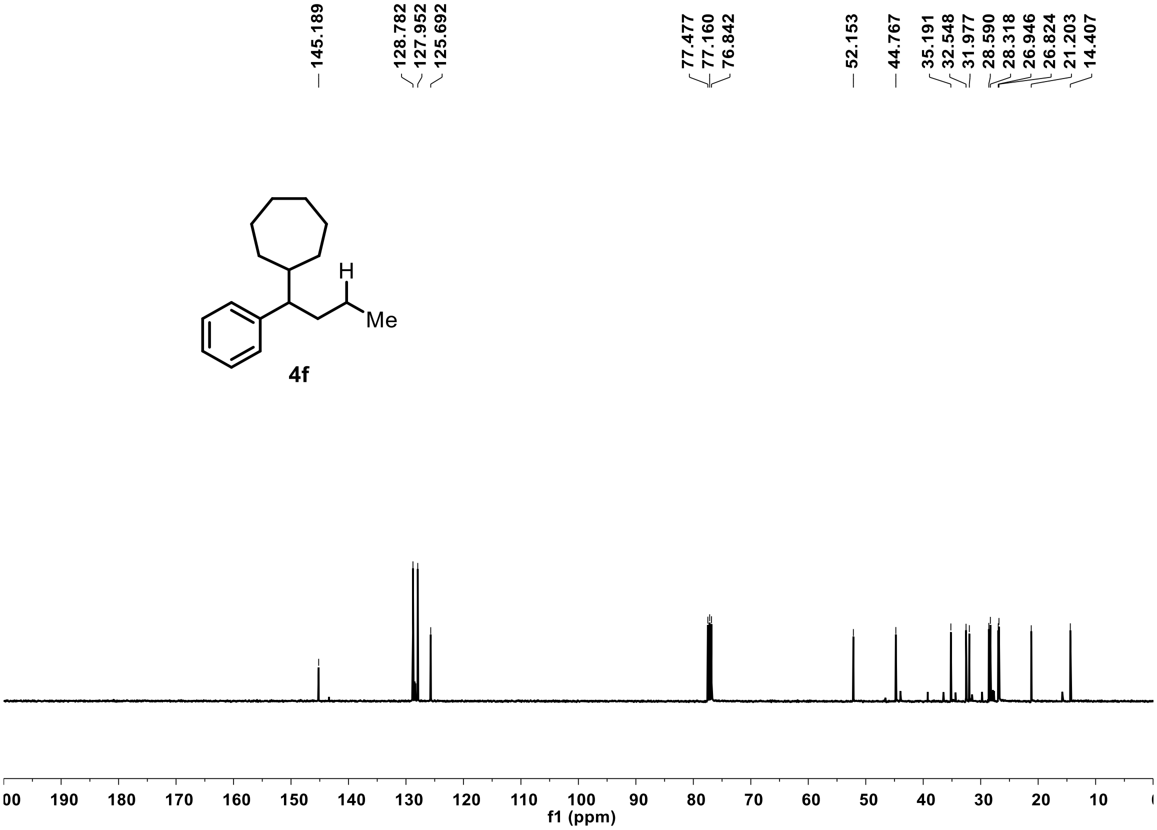
145.189
128.782
127.952
125.692
77.477
77.160
76.842
52.153
44.767
35.191
32.548
31.977
28.590
28.318
26.946
26.824
21.203
14.407
H
Me
4f
f1 (ppm)

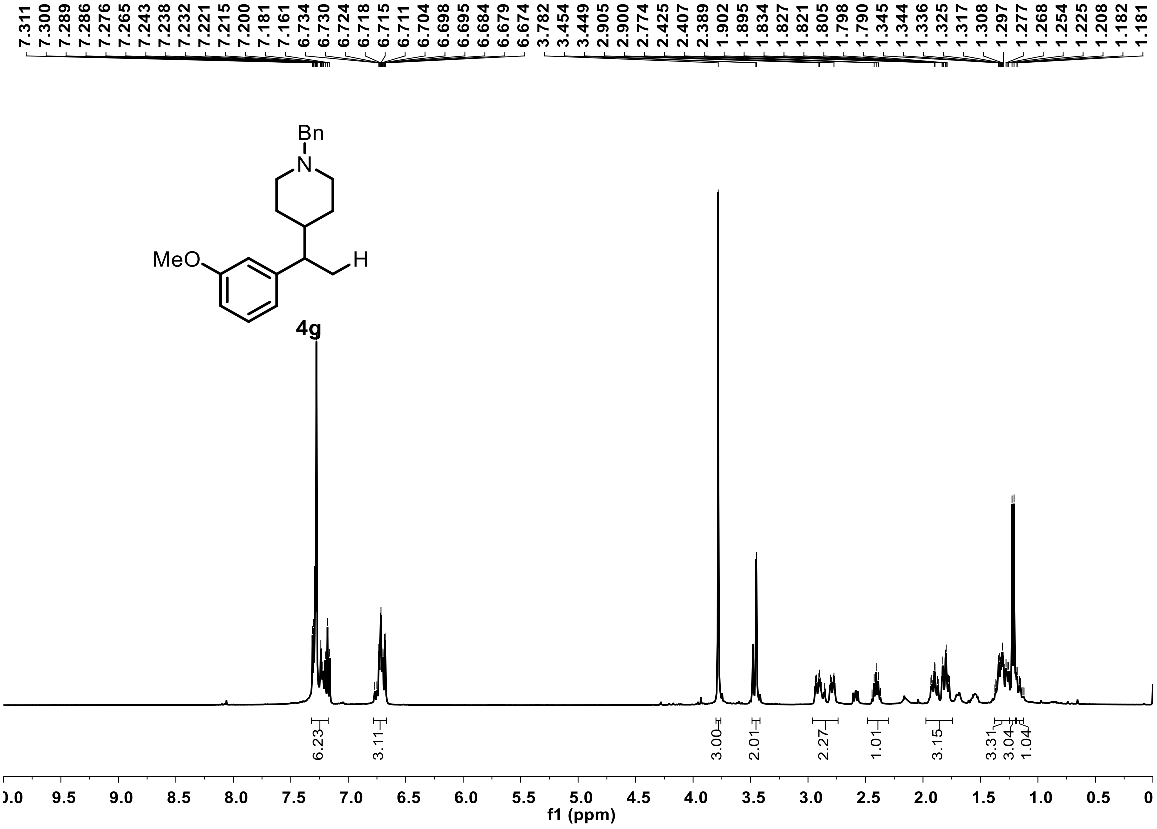
7.311 7.300 7.289 7.286 7.276 7.265 7.243 7.238 7.232 7.221 7.215 7.200 7.181 7.161 6.734 6.730 6.724 6.718 6.715 6.711 6.704 6.698 6.695 6.684 6.679 6.674 3.782 3.454 3.449 2.905 2.900 2.774 2.425 2.407 2.389 1.902 1.895 1.834 1.827 1.821 1.805 1.798 1.790 1.345 1.344 1.336 1.325 1.317 1.308 1.297 1.277 1.268 1.254 1.225 1.208 1.182 1.181
Bn
N
MeO
H
4g
6.23
3.11
3.00
2.01
2.27
1.01
3.15
3.31
3.04
1.04
f1 (ppm)

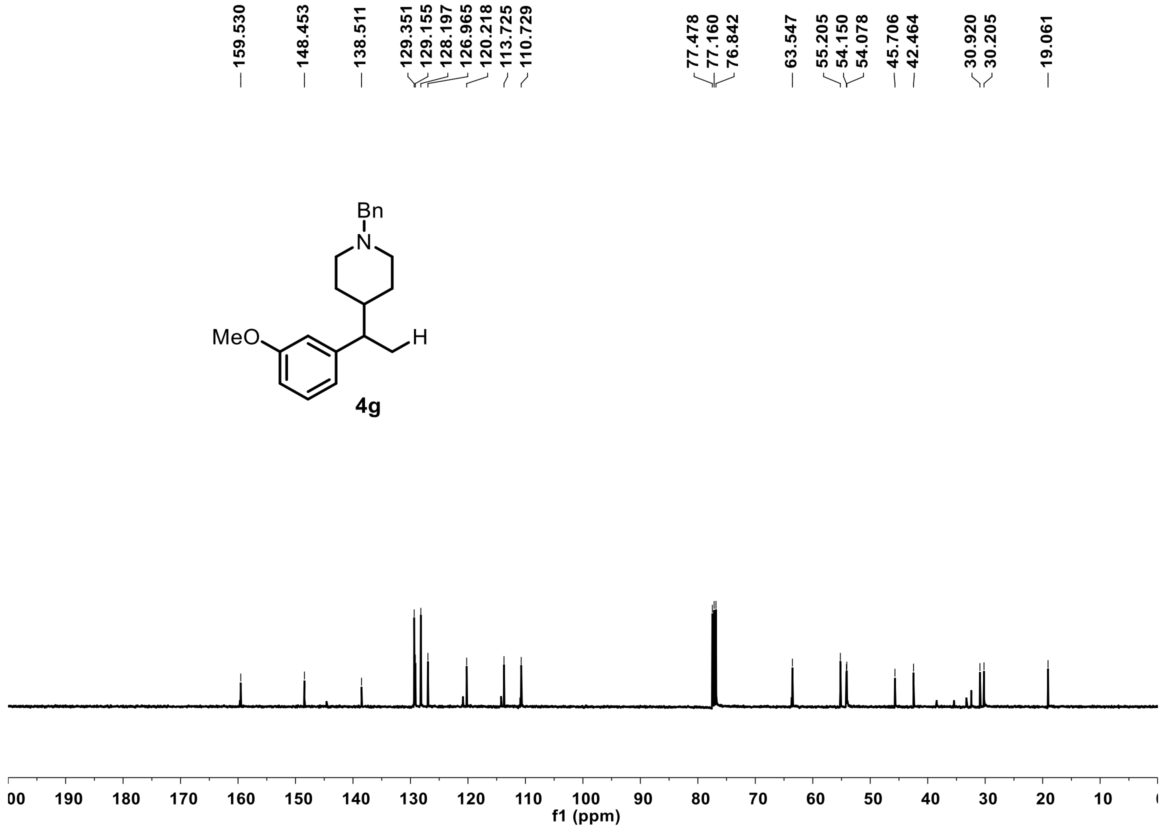
159.530
148.453
138.511
129.351
129.155
128.197
126.965
120.218
113.725
110.729
77.478
77.160
76.842
63.547
55.205
54.150
54.078
45.706
42.464
30.920
30.205
19.061
Bn
N
MeO
H
4g
f1 (ppm)

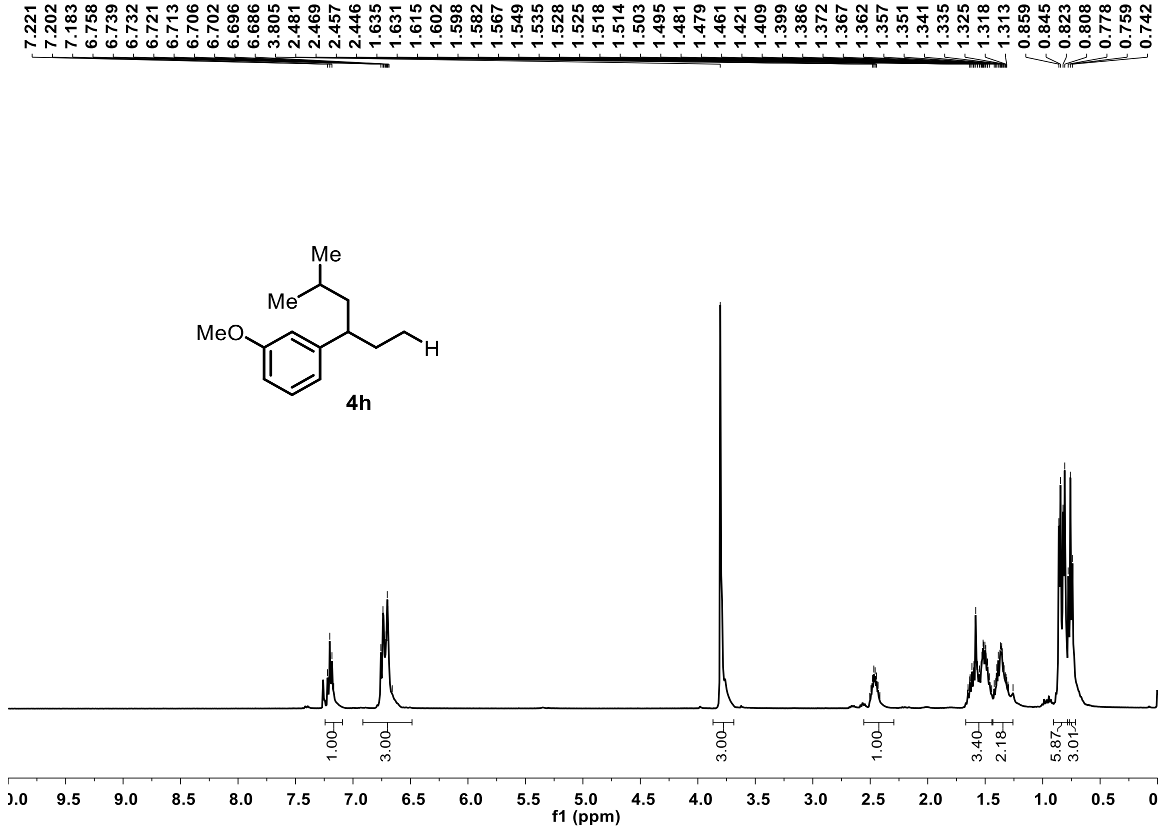
7.221
7.202
7.183
6.758
6.739
6.732
6.721
6.713
6.706
6.702
6.696
6.686
3.805
2.481
2.469
2.457
2.446
1.635
1.631
1.615
1.602
1.598
1.582
1.567
1.549
1.535
1.528
1.525
1.518
1.514
1.503
1.495
1.481
1.479
1.461
1.421
1.409
1.399
1.386
1.372
1.367
1.362
1.357
1.351
1.341
1.335
1.325
1.318
1.313
0.859
0.845
0.823
0.808
0.778
0.759
0.742
Me
Me
MeO
H
4h
1.00
3.00
3.00
1.00
3.40
2.18
5.87
3.01
f1 (ppm)

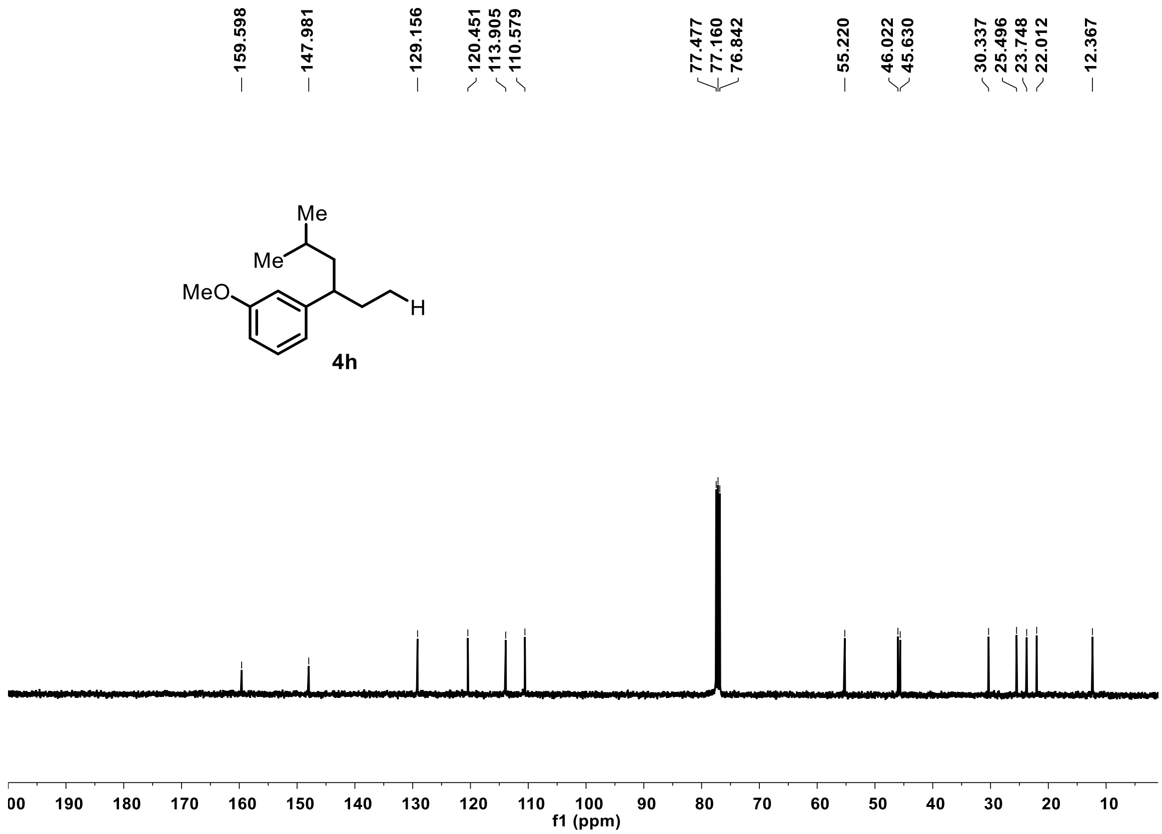
159.598
147.981
129.156
120.451
113.905
110.579
77.477
77.160
76.842
55.220
46.022
45.630
30.337
25.496
23.748
22.012
12.367
Me
Me
MeO
H
4h
00 190 180 170 160 150 140 130 120 110 100 90 80 70 60 50 40 30 20 10
f1 (ppm)

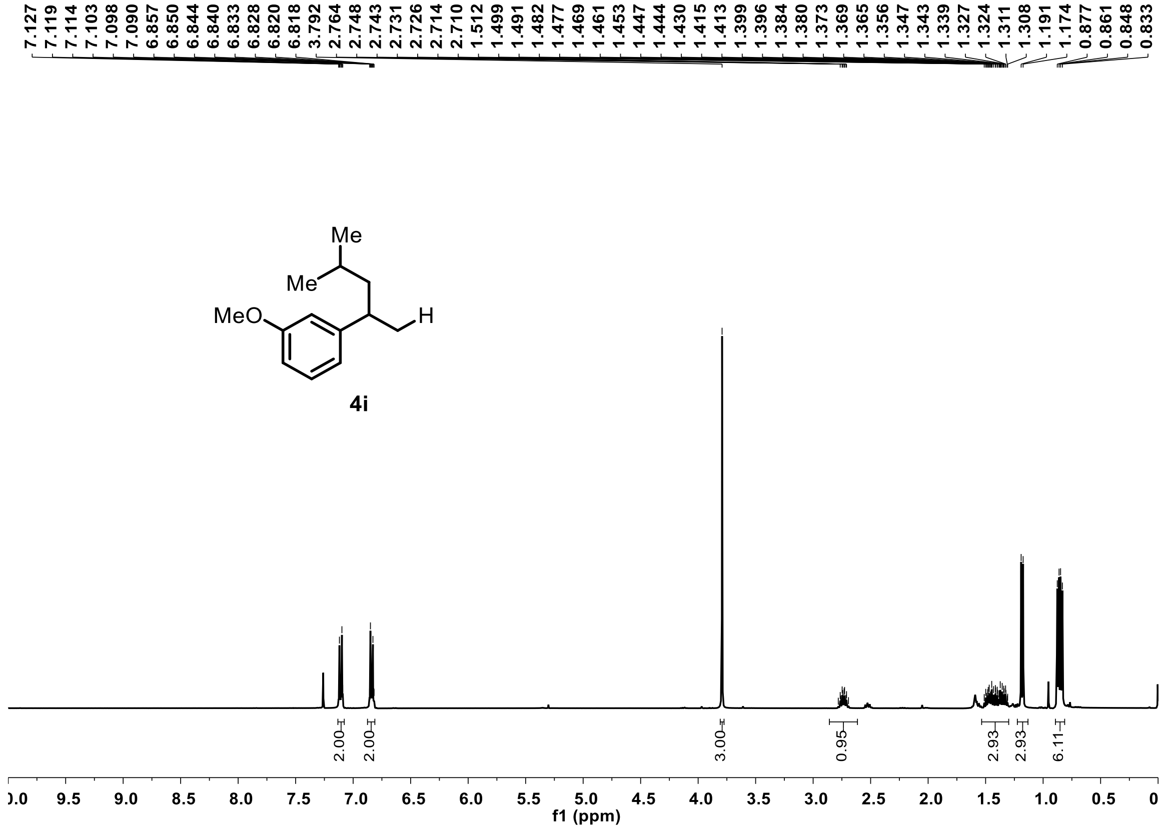
7.127
7.119
7.114
7.103
7.098
7.090
6.857
6.850
6.844
6.840
6.833
6.828
6.820
6.818
3.792
2.764
2.748
2.743
2.731
2.726
2.714
2.710
1.512
1.499
1.491
1.482
1.477
1.469
1.461
1.453
1.447
1.444
1.430
1.415
1.413
1.399
1.396
1.384
1.380
1.373
1.369
1.365
1.356
1.347
1.343
1.339
1.327
1.324
1.311
1.308
1.191
1.174
0.877
0.861
0.848
0.833
Me
Me
MeO
H
4i
2.00
2.00
3.00
0.95
2.93
2.93
6.11
).0 9.5 9.0 8.5 8.0 7.5 7.0 6.5 6.0 5.5 5.0 4.5 4.0 3.5 3.0 2.5 2.0 1.5 1.0 0.5 0
f1 (ppm)

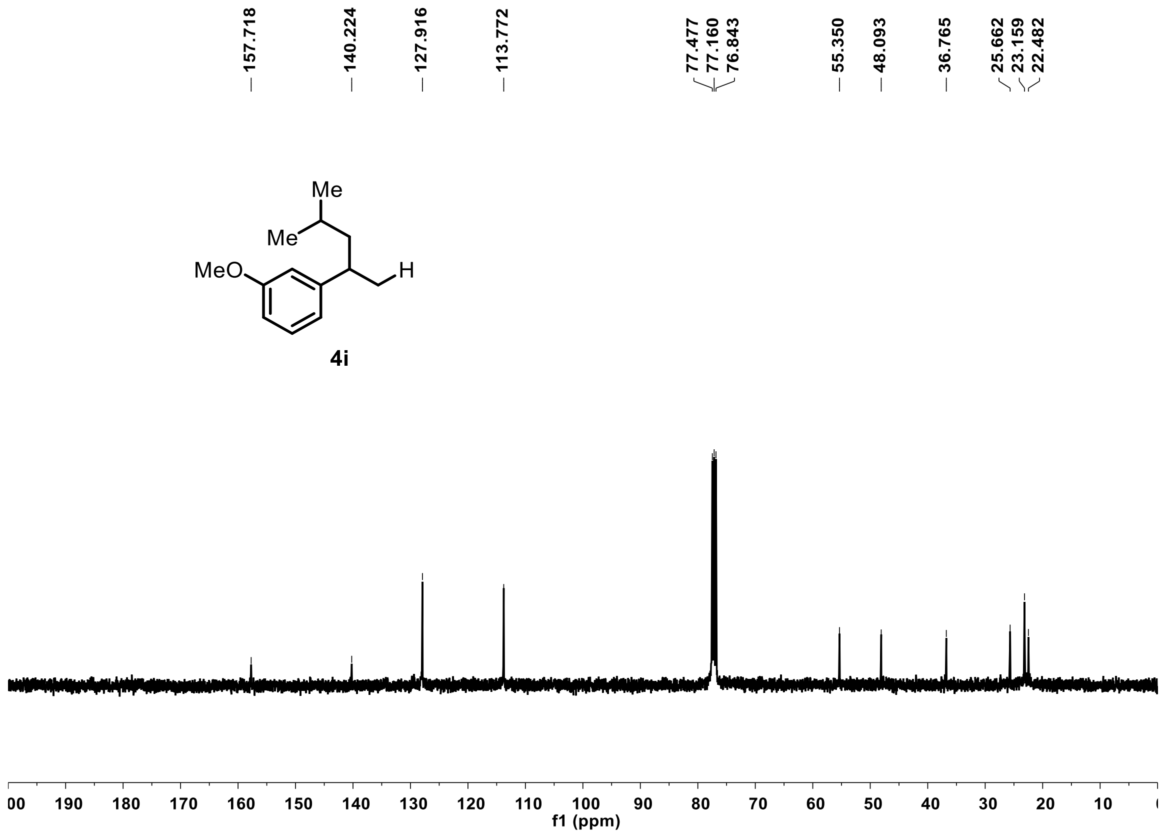

157.718
140.224
127.916
113.772
77.477
77.160
76.843
55.350
48.093
36.765
25.662
23.159
22.482
Me
Me
MeO
H
4i
00 190 180 170 160 150 140 130 120 110 100 90 80 70 60 50 40 30 20 10
f1 (ppm)

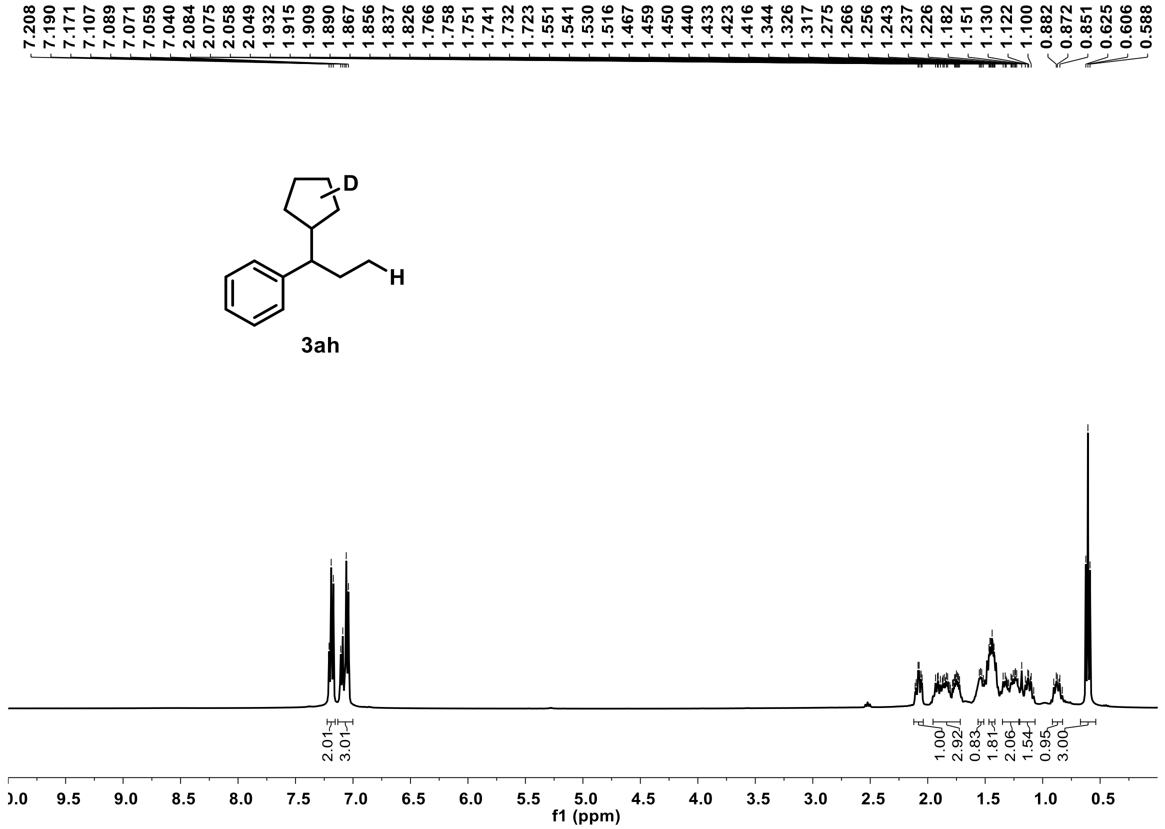

7.208
7.190
7.171
7.107
7.089
7.071
7.059
7.040
2.084
2.075
2.058
2.049
1.932
1.915
1.909
1.890
1.867
1.856
1.837
1.826
1.766
1.758
1.751
1.741
1.732
1.723
1.551
1.541
1.530
1.516
1.467
1.459
1.450
1.440
1.433
1.423
1.416
1.344
1.326
1.317
1.275
1.266
1.256
1.243
1.237
1.226
1.182
1.151
1.130
1.122
1.100
0.882
0.872
0.851
0.625
0.606
0.588
D
H
3ah
2.01
3.01
1.00
2.92
0.83
1.81
2.06
1.54
0.95
3.00
9.5 9.0 8.5 8.0 7.5 7.0 6.5 6.0 5.5 5.0 4.5 4.0 3.5 3.0 2.5 2.0 1.5 1.0 0.5
f1 (ppm)

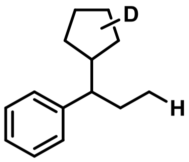
D
H

3ah

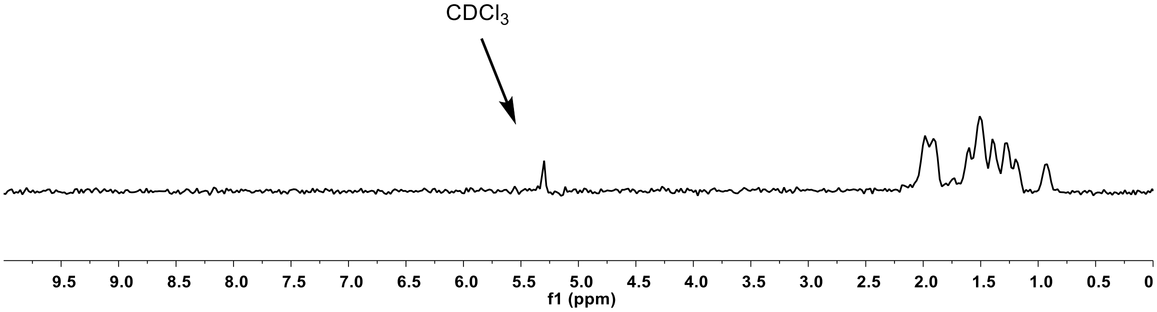
CDCl3
9.5 9.0 8.5 8.0 7.5 7.0 6.5 6.0 5.5 5.0 4.5 4.0 3.5 3.0 2.5 2.0 1.5 1.0 0.5 0
f1 (ppm)

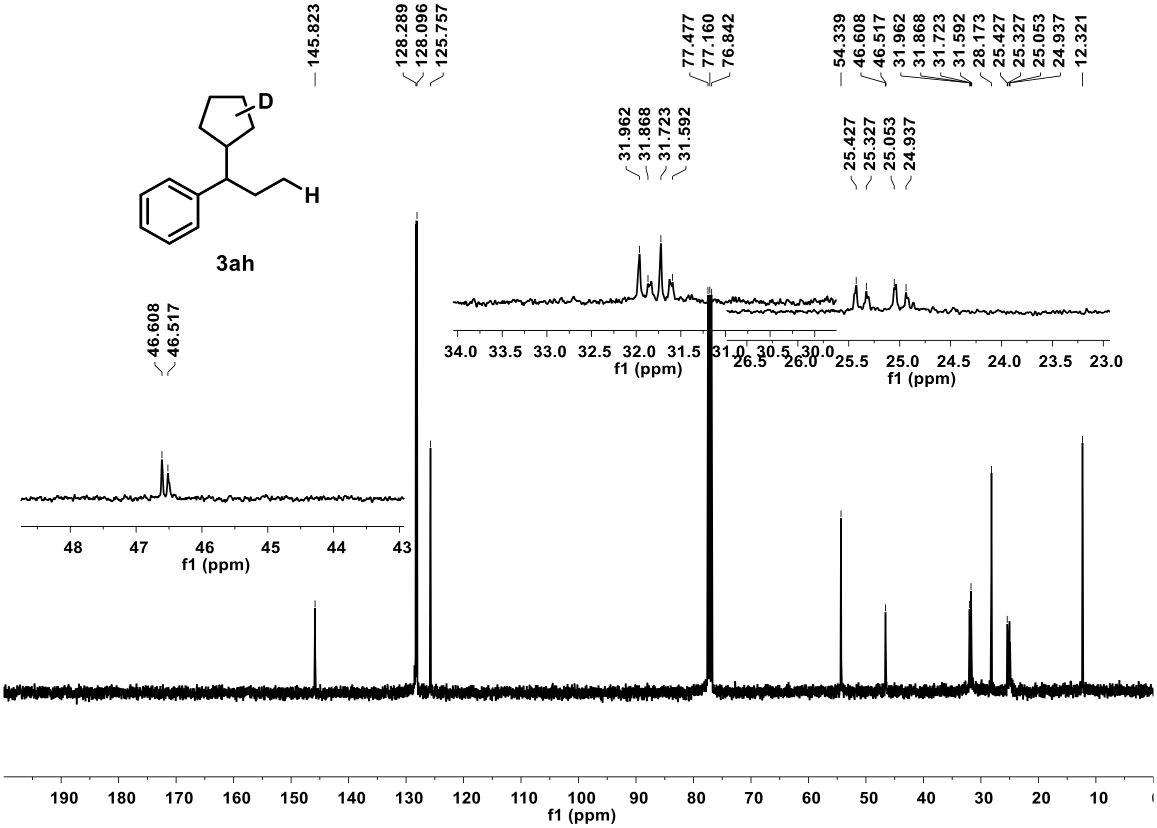
145.823
128.289
128.096
125.757
77.477
77.160
76.842
54.339
46.608
46.517
31.962
31.868
31.723
31.592
28.173
25.427
25.327
25.053
24.937
12.321
3ah
D
H
34.0 33.5 33.0 32.5 32.0 31.5 31.0 30.5 30.0
26.5 26.0 25.5 25.0 24.5 24.0 23.5 23.0
48 47 46 45 44 43
190 180 170 160 150 140 130 120 110 100 90 80 70 60 50 40 30 20 10
f1 (ppm)
